# Echocardiographic risk stratification in light chain and transthyretin amyloidosis: a meta-analysis

**DOI:** 10.1093/ehjopen/oeaf078

**Published:** 2025-08-22

**Authors:** David Koeckerling, Rohin K Reddy, Christian Eichhorn, Volker Braun, Yousif Ahmad, James P Howard, Fabian aus dem Siepen, Benjamin Meder, Norbert Frey, Derliz Mereles

**Affiliations:** Department of Cardiology, Angiology and Pulmonology, Heidelberg University Hospital, Im Neuenheimer Feld 410, 69120 Heidelberg, Germany; National Heart and Lung Institute, Imperial College London, Dovehouse Street, London SW36LY, UK; Nuffield Department of Population Health, University of Oxford, Old Road Campus, Oxford OX37LF, UK; Harvard T.H. Chan School of Public Health, Huntington Avenue, Boston, MA 02115, USA; Medical Faculty Mannheim, University of Heidelberg, Ludolf-Krehl-Strasse, 68167 Mannheim, Germany; Division of Cardiology, University of California, Parnassus Avenue, San Francisco, CA 94143, USA; National Heart and Lung Institute, Imperial College London, Dovehouse Street, London SW36LY, UK; Department of Cardiology, Angiology and Pulmonology, Heidelberg University Hospital, Im Neuenheimer Feld 410, 69120 Heidelberg, Germany; Department of Cardiology, Angiology and Pulmonology, Heidelberg University Hospital, Im Neuenheimer Feld 410, 69120 Heidelberg, Germany; Department of Cardiology, Angiology and Pulmonology, Heidelberg University Hospital, Im Neuenheimer Feld 410, 69120 Heidelberg, Germany; Department of Cardiology, Angiology and Pulmonology, Heidelberg University Hospital, Im Neuenheimer Feld 410, 69120 Heidelberg, Germany

**Keywords:** Amyloidosis, Cardiomyopathy, Transthyretin, Light chain, Echocardiography

## Abstract

**Aims:**

The role of echocardiography in amyloidosis prognostication remains undefined in international guidelines. This meta-analysis aims to evaluate associations between echocardiography-derived measurements and clinical outcomes in light chain (AL) and transthyretin (ATTR) amyloidosis.

**Methods and results:**

MEDLINE, Embase, Cochrane Library, and Google Scholar were systematically searched through July 2024 for studies reporting associations between echocardiographic variables [left ventricular global longitudinal strain (LV-GLS), LV ejection fraction (LVEF), tricuspid annular plane systolic excursion (TAPSE), interventricular septum diameter (IVSd), LV mass index (LVMi) and *E*/*e*′ ratios] and adverse events in AL or ATTR amyloidosis. Prespecified demographic items and clinical outcomes were extracted by two blinded, independent reviewers. The prespecified primary outcome was all-cause mortality. Random-effect models were applied to pool hazard ratios (HR). 94 studies comprising 16158 patients (*n* = 4788 AL, *n* = 8241 ATTR, *n* = 3129 mixed aetiologies) were included. Median follow-up was 22.3 (IQR, 16.9–31.4) months. Higher all-cause mortality risk (HR, 1.10: 95%CI, 1.08–1.12; *P* < 0.001) was observed per 1% LV-GLS decrement, consistent across AL and ATTR subgroups. Lower all-cause mortality risk was seen with increasing LVEF (per 1%) and TAPSE (per 1 mm) in the overall population (HR_LVEF_, 0.98; 95%CI, 0.98–0.98; *P* < 0.001; and HR_TAPSE_, 0.94; 95%CI, 0.93–0.95; *P* < 0.001) and in AL and ATTR subgroups. Higher *E*/*e*′ ratios (per 1 unit) were associated with all-cause mortality (HR, 1.02; 95%CI, 1.02–1.03; *P* < 0.001), consistent across AL and ATTR subtypes. No reliable associations between structural parameters (IVSd, LVMi) and clinical outcomes were found.

**Conclusion:**

Echocardiographic measures of biventricular deformation, systolic and diastolic function, were consistently associated with mortality in amyloidosis, while structural parameters were not. Echocardiography may have an important role in the initial risk stratification of cardiac amyloidosis.

## Graphical Abstract

**Graphical Abstract:**
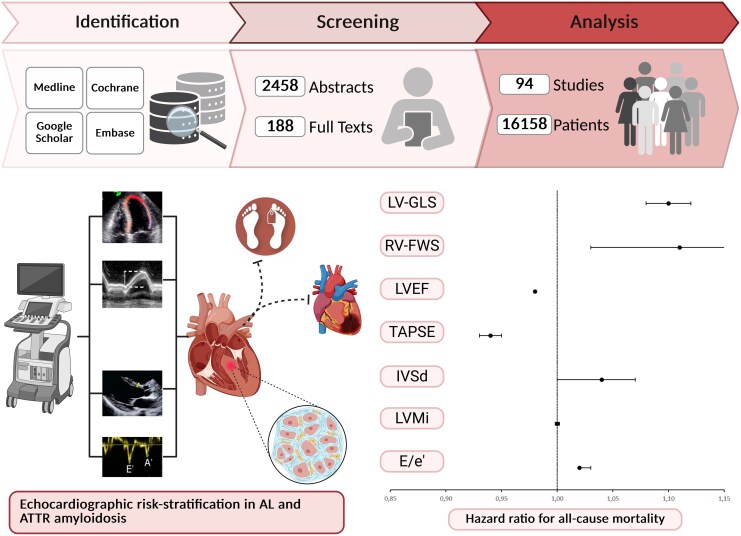

## Introduction

Systemic amyloidosis is characterized by extracellular aggregation of insoluble protein fibrils within various tissues. Cardiac involvement portends a poor prognosis, as accumulation of amyloidogenic proteins within the myocardium precipitates cellular injury, impairs compliance and affects conduction, resulting in an infiltrative cardiomyopathy with substantial mortality rates. Most cases of amyloidosis originate either from excessive secretion of abnormal immunoglobulin light chains by clonal proliferation of plasma cells, termed light chain (AL) amyloidosis, or from dissociation and misfolding of transthyretin, a transporter protein for thyroxine and retinol-binding protein 4 complex, termed transthyretin (ATTR) amyloidosis.^1,2^

If untreated, cardiac amyloidosis progresses rapidly with median survival ranging from 6–12 months for AL and 31–87 months for ATTR amyloidosis, depending on disease stage and presence of genetic variants.^3–5^ Over the last decade, significant strides were made in amyloidosis management with the emergence of immunotherapeutic regimens^6^ and the discovery of transthyretin stabilizers.^7,8^ Given the speed of disease progression and availability of life-saving treatment modalities, refined prognostication for AL and ATTR amyloidosis is essential in guiding surveillance strategies and therapeutic decision-making for clinicians.^9^

While traditional risk stratification approaches are primarily based on cardiac-specific biomarkers, such algorithms were derived prior to the advent of effective disease-modifying therapy and remain prone to influence by volume status, renal function, and concomitant cardiovascular pathologies. The association between magnetic resonance imaging parameters and cardiovascular outcomes in non-ischemic heart failure has recently been tested.^10^ As a widely accessible, inexpensive, and radiation-free imaging modality, echocardiography is uniquely positioned for enhancing risk assessment in amyloidosis. Nonetheless, the prognostic role of cardiac imaging remains undefined in current international guidelines.^1,11–13^ To this purpose, the present analysis aims to summarize the value of echocardiographic measures of myocardial deformation, biventricular systolic function, diastolic function and structural alterations in predicting clinical outcomes for patients with confirmed AL- and ATTR amyloidosis. The choice of echocardiographic parameters was based on pathophysiological relevance, clinical validity and availability, and representation within the literature.

## Methods

This meta-analysis was reported in line with the Meta-analysis Of Observational Studies in Epidemiology (MOOSE) guidance^14^ and was registered on the International Prospective Register of Systematic Reviews (PROSPERO), CRD42024562553.

### Search strategy

The electronic databases MEDLINE, Embase, Cochrane Library, and Google Scholar were searched by a clinical librarian (VB) from inception through July 2024. Conference abstracts were excluded. No language restrictions were applied. The search syntax was designed using keywords and MeSH headings around the concepts of (i) amyloidosis, (ii) echocardiographic parameters of interest, and (iii) adverse outcomes. The full search strategy is displayed in Supplementary material online, Appendix  *S1*. References of included studies were manually searched for additional relevant records. After the deletion of duplicates, abstract screening was performed by two independent, blinded reviewers (DK, RKR). Disputes were resolved by consensus.

### Study selection

Non-randomized diagnostic studies fulfilling the following criteria were deemed eligible: (i) the study population consisted of patients with confirmed AL or ATTR amyloidosis; (ii) at least one of the following echocardiographic parameters was investigated: Left ventricular global longitudinal strain (LV-GLS), right ventricular free wall strain (RV-FWS), LV ejection fraction (LVEF), tricuspid annular plane systolic excursion (TAPSE), LV mass index (LVMi), interventricular septum diameter (IVSd), or the *E/e*′ ratio; and iii) the report provided sufficient information on associations between echocardiographic variables and clinical outcomes for incorporation into quantitative synthesis. Potential overlap between study cohorts was explored for each echocardiographic parameter, endpoint, and subtype of amyloidosis using primary study centers and recruitment periods as overlap indicators. In case of relevant suspected overlap, only data from the study report with the largest study size, highest level of statistical adjustment for potential confounders, and lowest risk-of-bias score were incorporated into quantitative synthesis.

### Data extraction and risk-of-bias assessment

Review of full-text publications, data extraction, and risk-of-bias assessment were conducted by two independent investigators (DK, RKR); inter-reviewer discrepancies were resolved by consensus or discussion with senior investigators. Data extraction was performed at the study level using pre-piloted forms with respect to study characteristics (publication year, recruitment period, study size, amyloidosis type, number of centers, study design, inclusion criteria, outcomes, echo machines and strain software used, follow-up duration), patient characteristics [age, sex, body mass index, hypertension, hyperlipidaemia, diabetes mellitus, smoking history, atrial fibrillation, coronary artery disease, estimated glomerular filtration rate (eGFR), N-terminal pro-B-type natriuretic peptide (NT-proBNP), LVEF, IVSd, LVMi], and outcome data. Risk-of-bias assessment was conducted using the Quality in Prognosis Studies (QUIPS) tool.^15^

### Outcomes

The prespecified primary outcome was all-cause mortality, chosen due to its resistance to misclassification. Prespecified secondary outcomes were cardiovascular mortality and major adverse cardiac events (MACE). All-cause mortality was defined as death from any cause. Few studies considered the composite endpoint of all-cause mortality or heart transplantation; these were included in the analysis for all-cause mortality due to small numbers of heart transplantation events. Cardiovascular mortality was defined as death due to heart failure, arrhythmia, ischaemia, or cardiogenic shock. MACE was considered as any other combination of all-cause mortality, cardiovascular mortality, heart transplantation, arrhythmia, myocardial infarction, and heart failure hospitalization.

### Statistical analysis

All primary and subgroup analyses were prespecified. Hazard ratios (HR) and corresponding 95% confidence intervals (CI), the most appropriate measures for synthesizing time-to-event data, were selected as primary summary metrics.^16^ Where study reports provided multiple statistical models for a given association, estimates with the highest levels of adjustment for other echocardiographic covariates were extracted. Quantitative analysis was based on the most frequently reported units and increments (per 1% decrement in LV-GLS, RV-FWS; per 1% increase in LVEF; per 1 mm increase in TAPSE, IVSd; per 1 g/m^2^ increase in LVMi; per 1 unit increase in *E/e*′ ratios). Due to heterogeneous reporting of strain parameters (LV-GLS, RV-FWS), presented HRs reflect 1% decrements in their absolute value (i.e. moving towards 0%). Random-effect models were fitted with restricted maximum likelihood estimation, with CIs derived using the method of Hartung, Knapp, Sidik, and Jonkman (HKSJ), if at least three studies were available for quantitative synthesis.^17^ Statistical heterogeneity was quantified using the *I*^2^ statistic, describing the proportion of variability due to heterogeneity rather than chance. *P*-values for statistical heterogeneity were calculated using χ^2^ tests. Strict thresholds for interpretation are not recommended, but in general, an I^2^ statistic ≥50% and a χ^2^ test *P*-value <0.10 are considered to represent considerable heterogeneity.

Subgroup analysis was performed according to amyloidosis type, and according to strain software used for deformation-based parameters. Exploring potential sources of heterogeneity, prespecified mixed-effects meta-regression including covariates as a fixed effect was performed for the endpoint of all-cause mortality. Potential modification of observed associations through baseline age, LVEF, eGFR, NT-proBNP, proportion of male participants, and statistical adjustment was investigated. Meta-regression for patient age and sex was conducted separately for AL and ATTR populations due to inherent differences in patient demographics. Sensitivity analyses were performed by excluding studies reporting the composite endpoint of all-cause mortality and heart transplantation, and studies with the highest likelihood of residual cohort overlap. In additional non-prespecified sensitivity analyses, studies published from 2009 to 2016 were excluded to delineate the potential impact of variations in population characteristics and therapeutic regimes between historical and contemporary cohorts. Lastly, due to potential relationships between myocardial deformation, diastolic function, and ventricular mass, sensitivity analyses only including studies that reported LV-GLS, *E/e*′ ratios and LVMi concurrently, were undertaken. Small-study effects were assessed visually using funnel plots and statistically using linear regression. Meta-regression, subgroup analysis, and small-study effects assessment were performed, if at least 10 studies were available.^16^ All analyses were conducted in R, version 4.3.1, using the ‘meta’ package.

## Results

Following the screening of 2458 abstracts and review of 188 reports, 94 studies comprising 16158 patients were included.^18–111^ The study selection process is depicted in Supplementary material online, *Figure S1*, and study and patient characteristics are summarized in *Table 1*. The majority of studies were conducted in Europe (55%) and featured retrospective (76%), single-center (86%) designs. The median duration of follow-up was 22.3 months (IQR, 16.9–31.4 months). Compared to AL amyloidosis (*n* = 4788), patients with ATTR amyloidosis (*n* = 8241) were older (median age, 77.4 vs. 62.5 years), more commonly male (median male proportion 87.5% vs. 61.0%), more frequently demonstrated cardiovascular comorbidities (*Table 1*), and displayed lower glomerular filtration rates (median eGFR, 59.9 vs. 65.4 mL/min), ejection fractions (median LVEF, 48.3% vs. 58.0%) and NT-proBNP levels (median, 3432 vs. 5664 pg/mL or ng/L). Details regarding disease-modifying therapies, including chemo- and immunotherapeutic regimes for AL amyloidosis and transthyretin stabilizers for ATTR amyloidosis, are summarized in Supplementary material online, *Table S1*. Risk-of-bias assessment is reported in Supplementary material online, *Table S2*. Most studies exhibited moderate risks of bias (55%), followed by low (26%) and high risks for bias (19%). An overview of pooled hazard ratios, statistical heterogeneity, and small-study effects is provided in Supplementary material online, *Table S3*.

**Table 1:** Study and patient characteristics, stratified by type of amyloidosis

Study characteristics	Overall population	AL population	ATTR Population	Mixed population
Number of studies (number of patients)	94 (16158)	44 (4788)	28 (8241)	28 (3129)
Study size, *n* (%)				
*n* < 100	52 (52)	26 (59)	7 (25)	19 (68)
*n* ≥ 100, <500	44 (44)	17 (39)	18 (64)	9 (32)
*n* ≥ 500	4 (4)	1 (2)	3 (11)	0 (0)
Study design, *n* (%)				
Prospective	23 (24)	10 (23)	7 (25)	7 (25)
Retrospective	71 (76)	34 (77)	21 (75)	21 (75)
Single center	81 (86)	42 (95)	25 (89)	19 (68)
Multi-center	13 (14)	2 (5)	3 (11)	9 (32)
Study location, *n* (%)				
United States	25 (27)	11 (25)	7 (25)	8 (29)
Europe	52 (55)	18 (41)	18 (64)	20 (71)
Asia	21 (22)	15 (34)	4 (14)	2 (7)
Duration of follow-up in months, median (IQR)	22.3 (16.9–31.4)	21.4 (15.3–32.6)	22.9 (18.1–32.0)	22.3 (17.1–28.0)
Type of echo machine, *n* (%)				
Vivid, GE	44 (47)	24 (55)	11 (39)	10 (36)
iE33 or EPIQ, Philips	17 (18)	11 (25)	5 (18)	4 (14)
Other	7 (7)	3 (7)	2 (7)	2 (7)
Patient characteristics				
Age in years, median (IQR)	67.5 (61.6–74.6)	62.5 (60.0–66.3)	77.4 (74.9–78.8)	68.7 (66.0–71.3)
Proportion of males, median (IQR)	68.5 (61.0–79.0)	61.0 (54.9–65.0)	87.5 (80.3–89.7)	72.0 (69.0–75.0)
Body mass index in kg/m^2^, median (IQR)	25.0 (24.0–26.0)	24.0 (22.7–25.6)	25.7 (25.1–26.0)	25.3 (25.0–26.0)
Diabetes mellitus, median (IQR)	13.5 (9.2–17.0)	9.5 (6.0–11.1)	17.0 (15.7–22.5)	14.1 (11.9–17.3)
Hypertension, median (IQR)	40.9 (27.3–58.2)	30.6 (22.7–36.9)	56.8 (50.3–64.4)	42.0 (29.5–60.3)
Hyperlipidaemia, median (IQR)	40.9 (25.8–43.9)	31.8 (21.0–41.0)	39.3 (33.3–48.4)	37.0 (26.8–41.5)
Coronary artery disease, median (IQR)	16.9 (11.6–22.2)	11.6 (7.0–14.0)	26.0 (20.3–29.9)	16.8 (13.5–21.0)
Atrial fibrillation, median (IQR)	29.1 (15.3–45.5)	14.0 (9.5–18.0)	45.5 (34.6–55.3)	29.0 (23.5–44.4)
Laboratory parameters				
NT-proBNP in pg/mL or ng/L, median (IQR)	3960 (3316–5357)	5664 (3779–7024)	3432 (3197–4115)	3906 (3385–4575)
eGFR in mL/min, median (IQR)	62.0 (58.0–68.9)	65.4 (60.5–70.1)	59.9 (56.4–64.5)	62.0 (58.2–67.9)
Echocardiographic parameters				
LVEF in %, median (IQR)	53.0 (49.0–57.3)	58.0 (53.0–59.0)	48.3 (47.5–52.1)	52.0 (50.2–55.1)
IVS Diameter in mm, median (IQR)	16.0 (14.7–17.0)	14.8 (13.4–16.0)	16.9 (15.8–18.0)	16.3 (15.5–17.0)
LVM Index in g/m^2^, median (IQR)	143.0 (130.0–159.2)	132.0 (119.0–143.0)	159.0 (149.8–170.1)	152.1 (136.6–163.3)

### Deformation-based parameters

**Figure 1:**
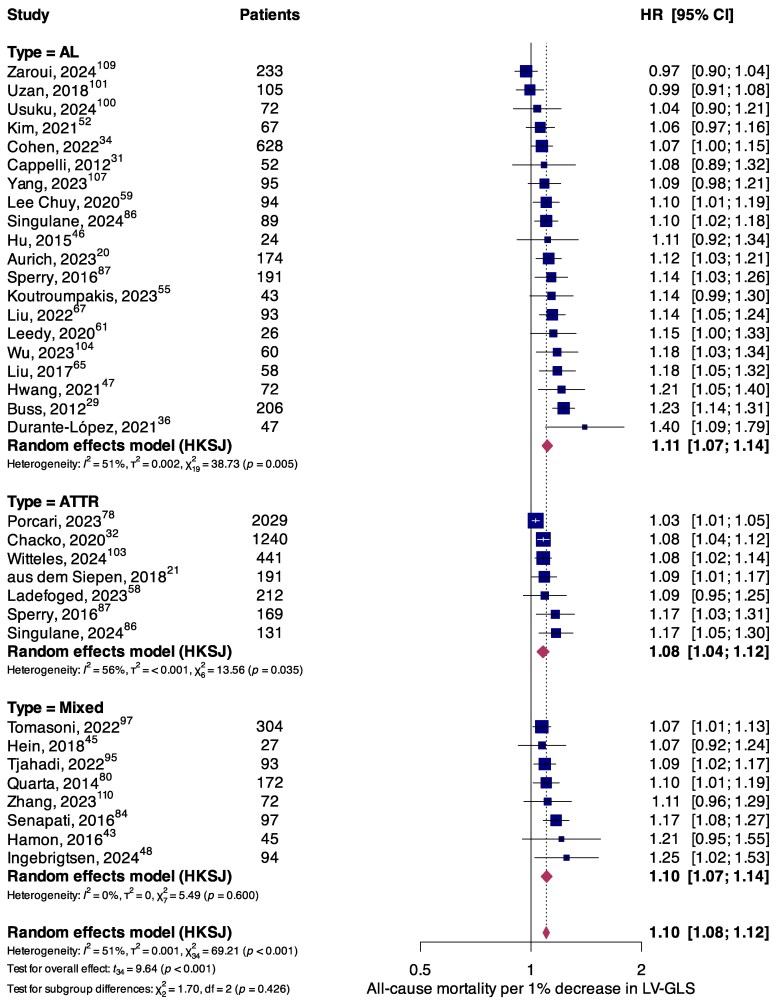
Forest plot summarizing the association between all-cause mortality and LV-GLS per 1% decrement, stratified according to amyloidosis aetiology.

The association between LV-GLS (per 1% decrement in absolute value) and all-cause mortality was explored by 35 (*n* = 7746) studies. The most commonly used software platform for LV-GLS assessment was EchoPAC PC, GE Healthcare, USA (43%), followed by TomTec Imaging Systems, Germany (21%). Significantly higher risks of all-cause mortality were observed per 1% decrease in LV-GLS for the overall population (HR, 1.10; 95%CI, 1.08–1.12; *P* < 0.001), consistent across AL (HR, 1.11; 95%CI, 1.07–1.14; *P* < 0.001) and ATTR subgroups (HR, 1.08; 95%CI, 1.04–1.12; *P* = 0.003) (*Figure 1*). When stratifying populations according to strain software used, results were directionally concordant with primary findings and retained statistical significance across software platforms (see Supplementary material online, *Figure S2*). However, there was evidence for a greater hazard of mortality per 1% LV-GLS decrement as assessed by TomTec Imaging (HR, 1.13; 95%CI, 1.09–1.17) compared to EchoPAC software (HR, 1.09; 95%CI, 1.06–1.12; *P*_interaction_ = 0.01). Findings for secondary endpoints of cardiovascular mortality (see Supplementary material online, *Figure S3*) and MACE (see Supplementary material online, *Figure S4*), and for RV-FWS (see Supplementary material online, *Figure S5* for all-cause mortality; *Figure S6* for MACE) are presented in the Supplementary material.

### Systolic function (LVEF and TAPSE)

**Figure 2:**
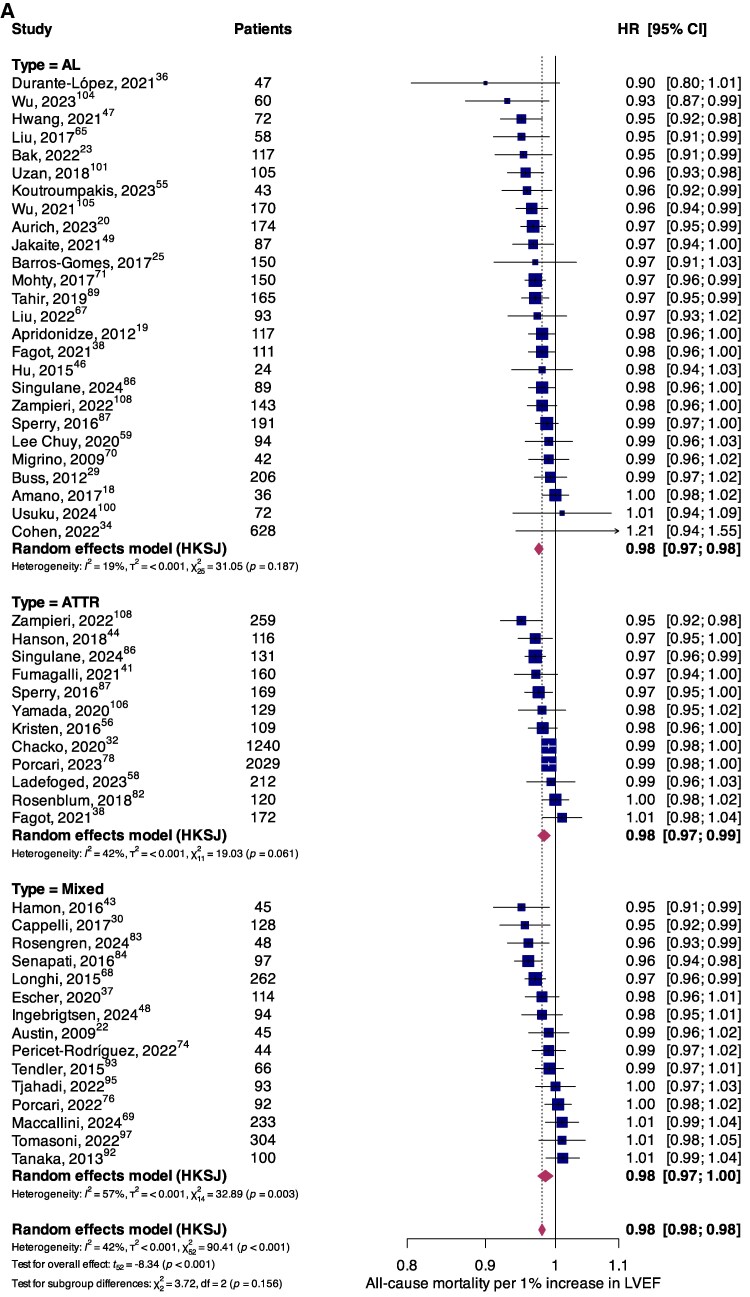

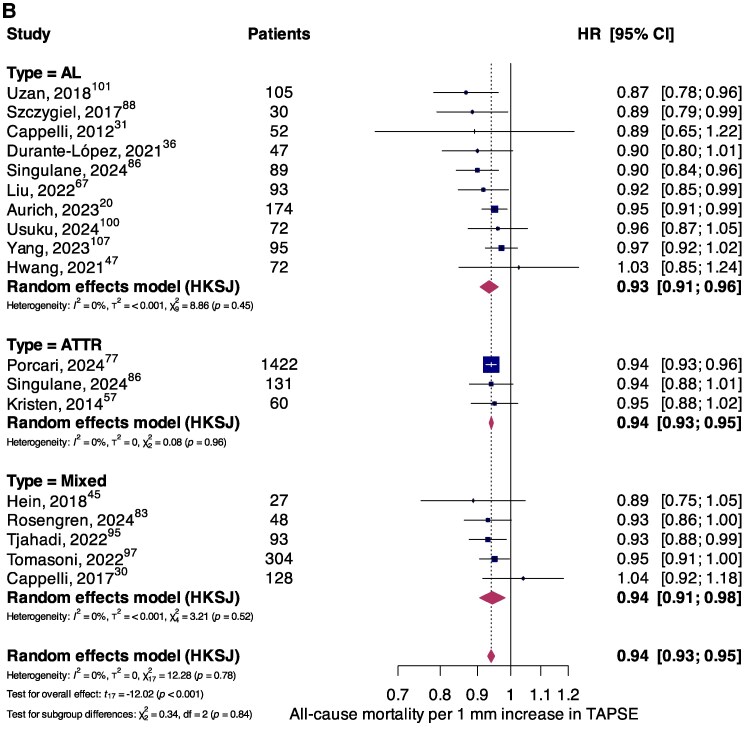
Forest plot summarizing the association between all-cause mortality and (*A*) LVEF per 1% increase, and (*B*) TAPSE per 1 mm increase, stratified according to amyloidosis aetiology.

The association between systolic functional parameters and mortality was reported by 53 (*n* = 9885) and 18 (*n* = 3042) studies for LVEF and TAPSE, respectively. Higher LVEF (per 1% increase) was associated with significantly lower all-cause mortality in the overall population (HR, 0.98; 95%CI, 0.98–0.98; *P* < 0.001), and in AL (HR, 0.98; 95%CI, 0.97–0.98; *P* < 0.001) and ATTR (HR, 0.98; 95%CI, 0.97–0.99; *P* = 0.002) subgroups (*Figure 2A*). Similarly, significantly lower all-cause mortality risk was observed per 1 mm increase in TAPSE in the overall population (HR, 0.94; 95%CI, 0.93–0.95; *P* < 0.001) with comparable findings among AL (HR, 0.93; 95%CI, 0.91–0.96; *P* < 0.001) and ATTR (HR, 0.94; 95%CI, 0.93–0.95; *P* < 0.001) subgroups (*Figure 2B*). Results for secondary endpoints of cardiovascular mortality (see Supplementary material online, *Figure S7* for LVEF; *Figure S9* for TAPSE) and MACE (see Supplementary material online, *Figure S8* for LVEF; *Figure S10* for TAPSE) are available in the Supplementary material.

### Structural parameters (IVSd and LVMi)

**Figure 3:**
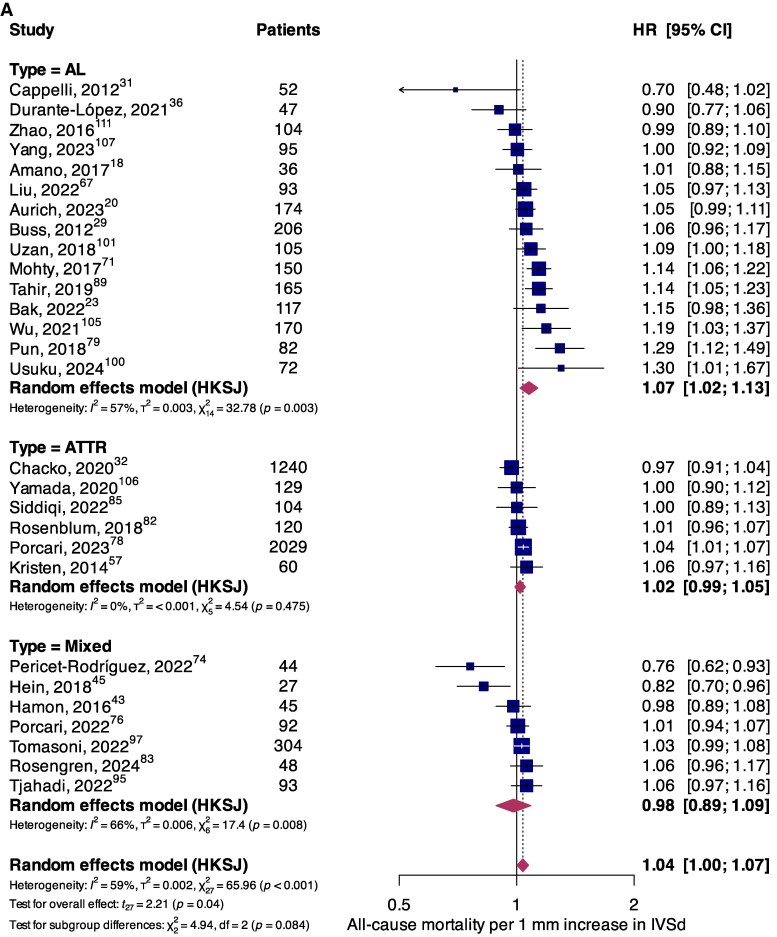

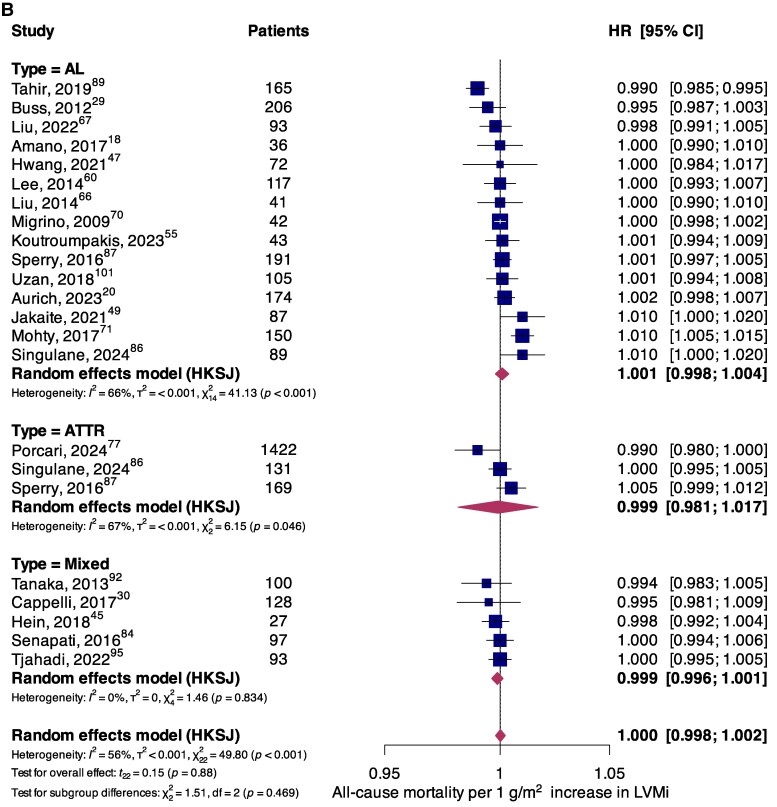
Forest plot summarizing the association between all-cause mortality and (*A*) IVSd per 1 mm increase, and (*B*) LVMi per 1 g/m^2^ increase, stratified according to amyloidosis aetiology.

The association between structural parameters and mortality was reported in 28 (*n* = 6003) and 23 (*n* = 3778) studies for IVSd and LVMi, respectively. In the overall population, a weak association between all-cause mortality and increasing IVSd (per 1 mm) was observed (HR, 1.04; 95%CI, 1.00–1.07; *P* = 0.04) (*Figure 3A*). In subgroup analysis, significantly higher risks of all-cause mortality per 1 mm increase in IVSd were identified in AL (HR, 1.07; 95%CI, 1.02–1.13; *P* = 0.01), but not in ATTR (HR, 1.02; 95%CI, 0.99–1.05; *P* = 0.15) and mixed cohorts (HR, 0.98; 95%CI, 0.89–1.09; *P* = 0.66). Similarly, no significant association between all-cause mortality and LVMi (per 1 g/m^2^ increase) was found for the overall population (HR, 1.000; 95%CI, 0.998–1.002; *P* = 0.88) or for AL (HR, 1.00; 95%CI, 1.00–1.00; *P* = 0.54) and ATTR subgroups (HR, 1.00; 95%CI, 0.98–1.02; *P* = 0.85) (*Figure 3B*). Finally, no associations between structural parameters and secondary endpoints of cardiovascular mortality (see Supplementary material online, *Figure S11* for IVSd; *Figure S13* for LVMi) and MACE (see Supplementary material online, *Figure S12* for IVSd; *Figure S14* for LVMi) were observed.

### Diastolic function (*E*/*e*′ ratio)

**Figure 4:**
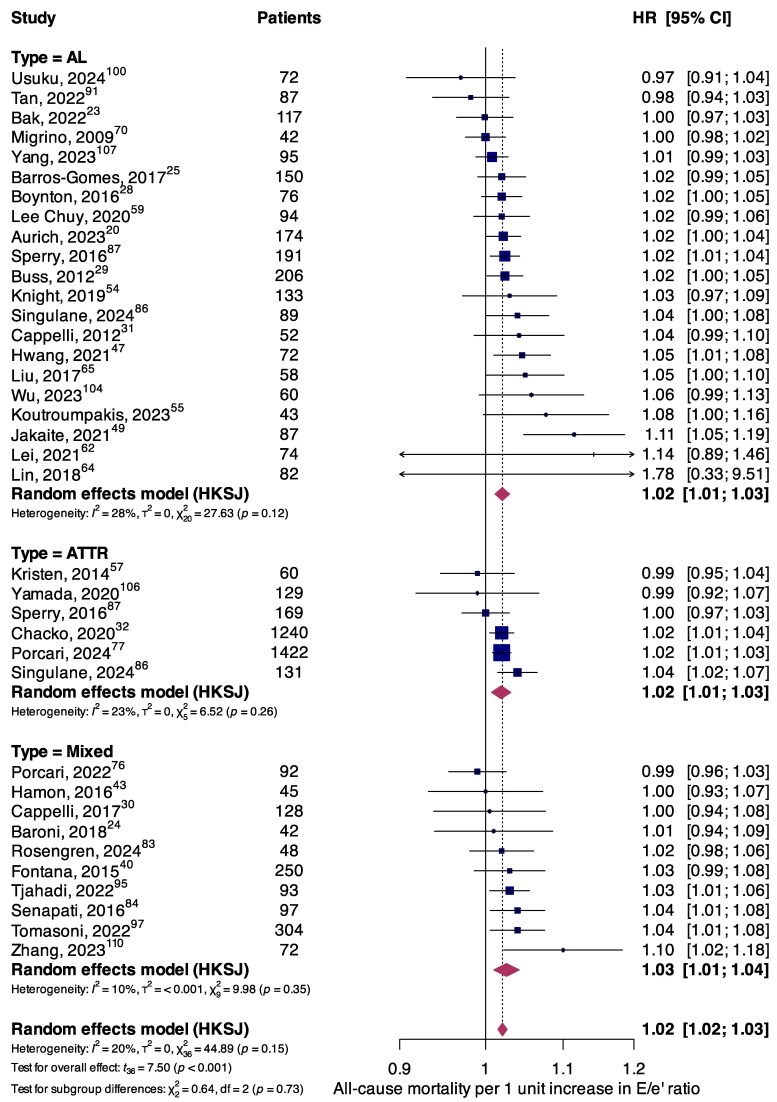
Forest plot summarizing the association between all-cause mortality and the *E*/*e*′ ratio per 1 unit increase, stratified according to amyloidosis aetiology.

Thirty-seven (*n* = 6376) studies investigated the association between mortality and the *E*/*e*′ ratio. Significantly higher all-cause mortality risk was seen per 1 unit increase in *E*/*e*′ ratios in the overall population (HR, 1.02; 95%CI, 1.02–1.03; *P* < 0.001), in AL (HR, 1.02; 95%CI, 1.01–1.03; *P* < 0.001) and in ATTR (HR, 1.02; 95%CI, 1.01–1.03; *P* = 0.009) cohorts (*Figure 4*). Findings regarding secondary endpoints of cardiovascular mortality (see Supplementary material online, *Figure S15*) and MACE (see Supplementary material online, *Figure S16*) are available in the Supplementary material.

### Meta-regression and sensitivity analyses

Meta-regression analysis did not identify consistent sources of heterogeneity, with associations remaining largely similar across baseline age, sex, eGFR, NT-proBNP, and LVEF (see Supplementary material online, *Table S4* for *P*-values; Supplementary material online, Appendix  *S2* for bubble plots). Statistical adjustment for covariates and baseline age (in AL cohorts) modified the association between LV-GLS and all-cause mortality; increasing age (per 1 year) and statistical adjustment were associated with a 0.31% (*P* = 0.01) and a 4.6% (*P* = 0.03) reduction in the HR, respectively. Sensitivity analyses excluding studies reporting the composite endpoint of all-cause mortality and heart transplantation were directionally concordant with primary analyses (see Supplementary material online, Appendix  *S3*). Exclusion of studies by Porcari et al. (2023–2024),^77,78^ due to potential residual cohort overlap with Chacko et al (2020),^32^ also produced findings consistent with main analyses (see Supplementary material online, Appendix  *S4*). Lastly, results generated by the exclusion of historical cohorts (see Supplementary material online, Appendix  *S5*) and sub-analysis of studies with concurrent assessment of LV-GLS, LVMi, and E/e′ ratios (see Supplementary material online, Appendix  *S6*) were concordant with primary analyses.

## Discussion

In this meta-analysis summarizing the value of echocardiographic parameters in risk-stratifying patients with AL and ATTR amyloidosis, the main findings were as follows: (i) measures of myocardial deformation (LV-GLS), systolic function (LVEF, TAPSE) and diastolic function (*E*/*e*′ ratio) were consistently associated with mortality in the overall population and across AL and ATTR subgroups, and (ii) no reliable associations were identified between clinical outcomes and structural parameters (IVSd and LVMi).

This meta-analysis is the first to quantitatively synthesize the contribution of multiparametric echocardiographic assessment to prognostication in AL and ATTR amyloidosis. Meta-regression results demonstrated the consistency of observed associations across baseline NT-proBNP, indicating the incremental utility of echocardiographic risk stratification over traditional cardiac biomarker-based algorithms. Despite prior reports on the independent prognostic value of several echocardiographic measurements in amyloidosis, translation into clinical practice has been hampered by focus on singular parameters, varying inclusion of echocardiographic covariates into statistical models and limited external validity due to single-center, small-scale study designs with heterogenous patient cohorts. Accordingly, guidance on risk prediction remains sparse in international consensus documents, while the role of cardiac imaging in general, and echocardiography in particular, is almost entirely undefined.^13^

Cardiac amyloidosis has traditionally been considered a predominantly restrictive cardiomyopathy with a diastolic dysfunction phenotype, yet the present analysis suggests a more complex interplay of systolic, diastolic, and deformation-based characteristics within the natural course of the disease and its prognosis. While LVEF is a conventional marker of cardiac function in heart failure and was persistently associated with clinical outcomes in the current analysis, biventricular ejection fractions tend to remain preserved until advanced disease stages, reflecting the later deterioration of radial function due to the predominant involvement of subendocardial longitudinal fibres.^54,112^ Conversely, diastolic and longitudinal systolic function, as measured by deformation-based variables, become impaired in the beginning stages of amyloid cardiomyopathy, making them promising early prognostic markers in the context of our findings. Although the predictive utility of LV-GLS measurement was retained across different software platforms (see Supplementary material online, *Figure S2*), the larger effect sizes observed with TomTec Imaging over EchoPAC software (*P*_interaction_ = 0.01) may relate to methodological variations in speckle-tracking. Speckle-tracking at the endocardial border (TomTec Imaging) may have prognostic advantages over full-wall strain measurement (EchoPAC) in amyloidosis as transmural dysfunction occurs late in the disease course.

Overall, individual associations between systolic functional parameters and clinical outcomes demonstrated clear statistical significance, yet effect sizes were moderate compared to other cardiac pathologies, such as acute coronary syndrome (ACS).^113^ Despite profound dysfunction of longitudinal fibres, ejection fraction tends to deteriorate only mildly in amyloidosis until late in the disease course. Consequently, a unit decrease in LVEF may not represent the same extent of myocardial impairment as in ACS, where LVEF more directly reflects contractile reserve. Similarly, deterioration in strain parameters is gradual with progressive amyloid deposition, while segmental wall motion abnormalities or focal reductions in strain following myocardial infarction may sharply increase risk and thereby confer greater effect sizes. Given the diffuse nature of amyloid deposition, impaired relaxation and right ventricular infiltration represent further major disturbances to cardiac performance, meaning that LV-GLS and LVEF individually do not fully capture all changes in cardiac haemodynamics that are relevant to overall prognosis. The complex interplay of myocardial deformation, and biventricular systolic and diastolic function in the progression of cardiac amyloidosis illustrates that multiparametric echocardiographic assessment may enhance prognostication to a greater extent than focus on any singular parameter.

In contrast to functional parameters, echocardiographic variables quantifying structural alterations appear to have limited value in risk stratification. Interventricular septal thickness and myocardial mass represent surrogate measures for the burden of interstitial amyloid fibril accumulation. However, mechanical tissue disruption by extracellular deposition of mature fibrils does not adequately explain the full extent of end-organ damage seen in amyloid cardiomyopathy, and a growing body of evidence indicates a central role for circulating, prefibrillar amyloidogenic proteins in mediating direct cardiotoxicity.^114,115^ In AL amyloidosis, circulating light chains directly affect cardiomyocyte contractility and relaxation through the generation of oxidative stress, activation of apoptotic pathways, and impairment of intracellular calcium handling.^116,117^ In preclinical models of ATTR amyloidosis, small transthyretin monomers and oligomeric intermediates, but not large aggregates and mature fibrils, induced cytotoxicity through interaction with membrane proteins, induction of apoptosis, and formation of superoxide radicals.^118,119^ The ability of imaging-based biomarkers to quantify the cardiotoxic impact of non-fibrillar amyloidogenic proteins may therefore be an essential determinant of their prognostic utility in AL and ATTR amyloidosis and may explain the differences between structural and functional parameters observed in the present study.

With the continuous advancement of imaging technologies, novel echocardiographic markers are undergoing evaluation of diagnostic and prognostic performance in cardiac amyloidosis. The scope and purpose of this meta-analysis focuses on established, commonly assessed functional and structural parameters available to cardiologists in a variety of clinical care settings. Left atrial strain assessment has recently demonstrated prognostic potential in several studies^61,67,100,120–125^ with atrial function increasingly being recognized as a key component of overall cardiac performance in various cardiovascular pathologies. While the paucity of available data and diversity of atrial strain assessment (reservoir strain, booster strain, conduit strain, and atrial stiffness) currently hamper meaningful inclusion into a study-level pooled analysis, future studies may wish to consider atrial strain assessment when evaluating the echocardiographic risk stratification of amyloidosis.

### Limitations

This meta-analysis is limited by underlying study quality due to the abundance of retrospective designs with small patient populations, short-to-midterm follow-up durations, and moderate-to-high risks of bias. Although most studies were conducted at single centers, amyloidosis care is commonly centralized within tertiary institutions and can therefore be considered representative of regional or even national patient populations. Inter-study heterogeneity exists regarding endpoint definitions, types of echo machines and strain software used, availability of disease-modifying therapy, and statistical adjustment for covariates. Accordingly, we employed random-effect models to account for inter-study variance and conducted meta-regression to explore potential modifying variables. Application of the HKSJ method is more conservative than traditional approaches and increases confidence in the reliability of results, particularly in the presence of smaller samples and between-study heterogeneity. Incorporation of baseline troponin as a potential effect modifier was not feasible due to varying assays, units, and troponin types among included studies. Similarly, the inclusion of disease-modifying therapies into meta-regression models was precluded by limited reporting in primary studies, diversity of therapeutic regimes, and absence of individual participant data. While potential cohort overlap was systematically evaluated and addressed, residual overlap cannot be excluded due to the absence of individual participant data. The paucity of data prohibited quantitative synthesis for arrhythmic outcomes and stratification of ATTR populations into hereditary and non-hereditary aetiologies, outlining additional targets for future research. Finally, small-study effects, including publication bias, may have resulted in the overestimation of observed associations between deformation-based parameters (LV-GLS, RV-FWS) and clinical outcomes (see Supplementary material online, *Table S3* for Egger test *P*-values; Supplementary material online, Appendix  *S7* for funnel plots).

## Conclusions

In this meta-analysis, several echocardiographic parameters related to myocardial deformation, biventricular systolic function, and diastolic function were consistently associated with adverse outcomes across AL and ATTR amyloidosis. Conversely, echocardiographic assessment of structural alterations, such as septal thickness and left ventricular mass, does not appear to substantially enhance risk prediction. Given the increasing availability of disease-modifying agents for AL and ATTR cardiomyopathy, echocardiography can facilitate resource allocation and selection of patients most likely to benefit from such therapies.^126^ While international guidelines lack consensus on the value of cardiac imaging in amyloidosis prognostication, our findings suggest that the incorporation of echocardiographic biomarkers into the initial risk assessment for AL and ATTR amyloidosis may be warranted.

## Supplementary Material

oeaf078_Supplementary_Data

## Data Availability

The data underlying this article will be shared on reasonable request to the corresponding author.

## Lead author biography

Dr. David Koeckerling is an academic clinical fellow in cardiology at the University Hospital Heidelberg, Germany. He completed his medical degree at Imperial College London, followed by the Academic Foundation Programme at the University of Oxford in 2021. In 2023, he earned his MD from the University of Bern, Switzerland. As an early career researcher, he has a keen interest in evidence synthesis and clinical research in cardiovascular medicine. His work focuses on improving patient outcomes through data-driven, evidence-based practices.
