# Supplementary material for: Echocardiographic risk stratification in light chain and transthyretin amyloidosis: a meta-analysis
Source: Eur Heart J Open. 2025 Aug 22;5(4):oeaf078. doi: 10.1093/ehjopen/oeaf078 (PMC12370300; doi:10.1093/ehjopen/oeaf078)

**Supplementary Material**

**Table S1:** Disease-modifying therapies used in included studies

**Table S2**: Risk-of-bias assessment at the study level using the QUIPS tool

**Table S3**: Summary of pooled hazard ratios and associated 95% confidence intervals, statistical heterogeneity and small-study effect assessment for individual echocardiographic parameters and clinical outcomes.

**Table S4**: Results of random-effects meta-regression assessing the strength of observed associations between echocardiographic measures and all-cause mortality adjusting for baseline age, LVEF, eGFR, NT-proBNP, proportion of male participants and statistical adjustment (unadjusted vs adjusted analysis)

**__________________________________________________________________________**

**Figure S1**: PRISMA flow-chart outlining the study selection process

**Figure S2**: Random-effects meta-analysis using the HKSJ method summarizing the association between all-cause mortality and LV-GLS, per 1% decrement, with subgroup analysis according to strain software used (EchoPAC, TomTec Imaging, others)

**Figure S3**: Random-effects meta-analysis using the HKSJ method summarizing the association between cardiovascular mortality and LV-GLS, per 1% decrement.

**Figure S4**: Random-effects meta-analysis using the HKSJ method summarizing the association between MACE and LV-GLS, per 1% decrement, including subgroup analysis for AL amyloidosis, ATTR amyloidosis and cohorts with mixed etiologies.

**Figure S5**: Random-effects meta-analysis using the HKSJ method summarizing the association between all-cause mortality RV-FWS per 1% decrement, with subgroup analysis for AL amyloidosis, ATTR amyloidosis and cohorts with mixed etiologies

**Figure S6**: Random-effects meta-analysis using the HKSJ method summarizing the association between MACE and RV-FWS, per 1% decrement.

**Figure S7**: Random-effects meta-analysis using the HKSJ method summarizing the association between cardiovascular mortality and LVEF, per 1% increase.

**Figure S8**: Random-effects meta-analysis using the HKSJ method summarizing the association between MACE and LVEF, per 1% increase, with subgroup analysis for AL amyloidosis, ATTR amyloidosis and cohorts with mixed etiologies.

**Figure S9**: Random-effects meta-analysis using the HKSJ method summarizing the association between cardiovascular mortality and TAPSE, per 1mm increase.

**Figure S10**: Random-effects meta-analysis using the HKSJ method summarizing the association between MACE and TAPSE, per 1mm increase.

**Figure S11**: Random-effects meta-analysis using the HKSJ method summarizing the association between cardiovascular mortality and IVSd, per 1mm increase.

**Figure S12**: Random-effects meta-analysis using the HKSJ method summarizing the association between MACE and IVSd, per 1mm increase.

**Figure S13**: Random-effects meta-analysis using the HKSJ method summarizing the association between cardiovascular mortality and LVMi, per 1g/m^2^ increase.

**Figure S14**: Random-effects meta-analysis using the HKSJ method summarizing the association between MACE and LVMi, per 1g/m^2^ increase.

**Figure S15**: Random-effects meta-analysis using the HKSJ method summarizing the association between cardiovascular mortality and the E/e’ ratio, per 1 unit increase.

**Figure S16**: Random-effects meta-analysis using the HKSJ method summarizing the association between MACE and the E/e’ ratio, per 1 unit increase.

___________________________________________________________________________

**Appendix 1**: Search strategy

**Appendix 2**: Mixed-effects meta-regression bubble plots evaluating baseline patient age, proportion of male participants, eGFR, NT-proBNP and LVEF as potential effect modifiers for the associations between echocardiographic variables and all-cause mortality

**Appendix 3**: Sensitivity analyses excluding studies that reported the composite endpoint of all-cause mortality and heart transplantation

**Appendix 4**: Sensitivity analyses excluding studies by Porcari et al (2023/2024), due to potential residual overlap with Chacko et al (2020)

**Appendix 5:** Sensitivity analyses excluding historical cohorts (2009-2016)

**Appendix 6**: Sensitivity analysis including studies with concurrent assessment of LV-GLS, LVMi and E/e’ ratios.

**Appendix 7**: Funnel plots for visual assessment of small-study effects

Table S1: Disease-modifying therapies used in included studies

| First author | Year | Amyloidosis type | ASCT_n | ASCT_% | Bortezomib_n | Borte_% | Daratumumab_n | Dara_% | Tafamidis_n | Tafa_% | Acoramidis_n |
| --- | --- | --- | --- | --- | --- | --- | --- | --- | --- | --- | --- |
| Amano | 2017 | AL | 0 | 0 | 4 | 11.11 | 0 | 0 |  |  |  |
| Apridonidze | 2012 | AL | NA |  | NA |  | NA |  |  |  |  |
| Aurich | 2023 | AL | 1 | 0.57 | 82 | 47.13 | 0 | 0 |  |  |  |
| aus dem Siepen | 2018 | ATTR |  |  |  |  |  |  | NA |  | NA |
| Austin | 2009 | Mixed | NA |  | NA |  | NA |  | NA |  | NA |
| Bak | 2022 | AL | 29 | 24.79 | 65 | 55.56 | 0 | 0 |  |  |  |
| Baroni | 2018 | Mixed | NA |  | NA |  | NA |  | NA |  | NA |
| Barros-Gomes | 2017 | AL | NA |  | NA |  | NA |  |  |  |  |
| Binder | 2019 | ATTR |  |  |  |  |  |  | 5 | 8.06 | 0 |
| Binder | 2019 | AL | NA |  | NA |  | NA |  |  |  |  |
| Bodez | 2015 | Mixed | NA |  | NA |  | NA |  | 20 | 24.39 | 0 |
| Boynton | 2016 | AL | NA |  | NA |  | NA |  |  |  |  |
| Buss | 2012 | AL | 0 | 0 | 0 | 0 | 0 | 0 |  |  |  |
| Cappelli | 2012 | AL | NA |  | NA |  | NA |  |  |  |  |
| Cappelli | 2017 | Mixed | NA |  | NA |  | NA |  | NA |  | NA |
| Chacko | 2020 | ATTR |  |  |  |  |  |  | 0 | 0 | 0 |
| Cipriani | 2022 | ATTR |  |  |  |  |  |  | NA |  | NA |
| Cipriani | 2022 | AL | NA |  | NA |  | NA |  |  |  |  |
| Cohen | 2022 | AL | 0 | 0 | 628 | 100 | 0 | 0 |  |  |  |
| Dongiglio | 2022 | Mixed | NA |  | NA |  | NA |  | NA |  | NA |
| Durante-López | 2021 | AL | NA |  | 47 | 100 | NA |  |  |  |  |
| Escher | 2020 | Mixed | 14 | 12.28 | NA |  | NA |  | 3 | 2.63 | 0 |
| Fagot | 2021 | ATTR |  |  |  |  |  |  | NA |  | NA |
| Fagot | 2021 | AL | NA |  | NA |  | NA |  |  |  |  |
| Fine | 2020 | Mixed | NA |  | NA |  | NA |  | 0 | 0 | 0 |
| Fontana | 2015 | Mixed | NA |  | NA |  | NA |  | NA |  | NA |
| Fumagalli | 2021 | ATTR |  |  |  |  |  |  | 0 | 0 | 0 |
| Galat | 2015 | ATTR |  |  |  |  |  |  | NA |  | NA |
| Hamon | 2016 | Mixed | NA |  | NA |  | NA |  | 7 | 15.56 | 0 |
| Hanson | 2018 | ATTR |  |  |  |  |  |  | 0 | 0 | 0 |
| Hein | 2018 | Mixed | NA |  | NA |  | NA |  | NA |  | NA |
| Hu | 2015 | AL | 12 | 50 | NA |  | NA |  |  |  |  |
| Hwang | 2021 | AL | 19 | 26.39 | 31 | 43.06 | NA |  |  |  |  |
| Ingebrigtsen | 2024 | Mixed | NA |  | NA |  | NA |  | NA |  | NA |
| Jakaitė | 2021 | AL | 25 | 28.74 | 68 | 78.16 | NA |  |  |  |  |
| Kado | 2016 | Mixed | NA |  | NA |  | NA |  | NA |  | NA |
| Kharoubi | 2021 | ATTR |  |  |  |  |  |  | NA |  | NA |
| Kim | 2021 | AL | NA |  | 30 | 44.78 | NA |  |  |  |  |
| Klarskov | 2023 | ATTR |  |  |  |  |  |  | 0 | 0 | 0 |
| Knight | 2019 | AL | NA |  | NA |  | NA |  |  |  |  |
| Koutroumpakis | 2023 | AL | 22 | 51.16 | 31 | 72.09 | 4 | 9.3 |  |  |  |
| Kristen | 2016 | ATTR |  |  |  |  |  |  | NA |  | NA |
| Kristen | 2014 | ATTR |  |  |  |  |  |  | NA |  | NA |
| Ladefoged | 2023 | ATTR |  |  |  |  |  |  | NA |  | NA |
| Lee Chuy | 2020 | AL | 23 | 24.47 | 55 | 58.51 | NA |  |  |  |  |
| Lee | 2014 | AL | 19 | 16.24 | NA |  | NA |  |  |  |  |
| Leedy | 2020 | AL | 26 | 100 | NA |  | NA |  |  |  |  |
| Lei | 2021 | AL | NA |  | NA |  | NA |  |  |  |  |
| Licordari | 2022 | ATTR |  |  |  |  |  |  | NA |  | NA |
| Lin | 2018 | AL | 2 | 2.44 | 9 | 10.98 | NA |  |  |  |  |
| Liu | 2014 | AL | NA |  | NA |  | NA |  |  |  |  |
| Liu | 2017 | AL | NA |  | NA |  | NA |  |  |  |  |
| Liu | 2022 | AL | 5 | 5.38 | 64 | 68.82 | NA |  |  |  |  |
| Longhi | 2015 | Mixed | NA |  | NA |  | NA |  | NA |  | NA |
| Maccallini | 2024 | Mixed | NA |  | NA |  | NA |  | NA |  | NA |
| Migrino | 2009 | AL | 13 | 30.95 | NA |  | NA |  |  |  |  |
| Mohty | 2017 | AL | NA |  | NA |  | NA |  |  |  |  |
| Ozbay | 2024 | ATTR |  |  |  |  |  |  | 87 | 80.56 | 0 |
| Palmiero | 2023 | Mixed |  |  |  |  |  |  | 27 | 29.35 | 0 |
| Pericet-Rodríguez | 2022 | Mixed | NA |  | NA |  | NA |  | NA |  | NA |
| Pislaru | 2019 | Mixed | NA |  | NA |  | NA |  | NA |  | NA |
| Porcari | 2022 | Mixed | 6 | 6.52 | 26 | 28.26 | 1 | 1.09 | NA |  | NA |
| Porcari | 2024 | ATTR |  |  |  |  |  |  | 0 | 0 | 0 |
| Porcari | 2023 | ATTR |  |  |  |  |  |  | 0 | 0 | 0 |
| Pun | 2018 | AL | 82 | 100 | NA |  | NA |  |  |  |  |
| Quarta | 2014 | Mixed | 19 | 11.05 | NA |  | NA |  | NA |  | NA |
| Rapezzi | 2011 | ATTR |  |  |  |  |  |  | NA |  | NA |
| Rosenblum | 2018 | ATTR |  |  |  |  |  |  | 16 | 13.33 | 0 |
| Rosengren | 2024 | Mixed | NA |  | NA |  | NA |  | NA |  | NA |
| Senapati | 2016 | Mixed | NA |  | NA |  | NA |  | NA |  | NA |
| Siddiqi | 2022 | ATTR |  |  |  |  |  |  | 0 | 0 | 0 |
| Singulane | 2024 | AL | NA |  | NA |  | NA |  |  |  |  |
| Singulane | 2024 | ATTR |  |  |  |  |  |  | 6 | 4.58 | 0 |
| Sperry | 2016 | ATTR |  |  |  |  |  |  | NA |  | NA |
| Sperry | 2016 | AL | 16 | 8.38 | NA |  | NA |  |  |  |  |
| Szczygieł | 2017 | AL | NA |  | NA |  | NA |  |  |  |  |
| Tahir | 2019 | AL | 61 | 36.97 | NA |  | NA |  |  |  |  |
| Takashio | 2023 | ATTR |  |  |  |  |  |  | 125 | 100 | 0 |
| Tan | 2022 | AL | 1 | 1.15 | 53 | 60.92 | NA |  |  |  |  |
| Tanaka | 2013 | Mixed | NA |  | NA |  | NA |  | NA |  | NA |
| Tendler | 2015 | Mixed | NA |  | NA |  | NA |  | NA |  | NA |
| Ternacle | 2016 | Mixed | NA |  | NA |  | NA |  | NA |  | NA |
| Tjahadi | 2022 | Mixed | NA |  | NA |  | NA |  | NA |  | NA |
| Tomasoni | 2023 | Mixed | NA |  | NA |  | NA |  | NA |  | NA |
| Tomasoni | 2022 | Mixed | NA |  | NA |  | NA |  | NA |  | NA |
| Usuku | 2022 | ATTR |  |  |  |  |  |  | NA |  | NA |
| Usuku | 2023 | AL | NA |  | 21 | 32.31 | 36 | 55.38 |  |  |  |
| Usuku | 2024 | AL | NA |  | 23 | 31.94 | 38 | 52.78 |  |  |  |
| Uzan | 2018 | AL | NA |  | NA |  | NA |  |  |  |  |
| Vranian | 2018 | ATTR |  |  |  |  |  |  |  |  |  |
| Witteles | 2024 | ATTR |  |  |  |  |  |  | 264 | 59.86 | 0 |
| Wu | 2023 | AL | NA |  | NA |  | NA |  |  |  |  |
| Wu | 2021 | AL | 2 | 1.18 | 39 | 22.94 | 0 | 0 |  |  |  |
| Yamada | 2020 | ATTR |  |  |  |  |  |  | 4 | 3.1 | NA |
| Yang | 2023 | AL | 0 | 0 | 30 | 31.58 | NA |  |  |  |  |
| Zampieri | 2022 | ATTR |  |  |  |  |  |  | 8 | 3.09 | 0 |
| Zampieri | 2022 | AL | NA |  | NA |  | NA |  |  |  |  |
| Zaroui | 2024 | AL | NA |  | 229 | 98.28 | 4 | 1.72 |  |  |  |
| Zhang | 2023 | Mixed | 5 | 6.94 | NA |  | NA |  | NA |  | NA |
| Zhao | 2016 | AL | 0 | 0 | NA |  | NA |  |  |  |  |

Table S2: Risk-of-bias assessment at the study level using the QUIPS tool

| First author | year | Participation | Attrition | Prognostic factor measurement | Outcome Measurement | Confounding | Statistical analysis reporting | OVERALL |
| --- | --- | --- | --- | --- | --- | --- | --- | --- |
| Amano | 2017 | High | Low | Moderate | Low | Moderate | Low | Moderate |
| Apridonidze | 2012 | Low | Moderate | Low | Low | Moderate | Moderate | Moderate |
| Aurich | 2023 | Moderate | Low | Moderate | Low | Low | Low | Low |
| aus dem Siepen | 2018 | Low | Low | High | Low | High | Moderate | Moderate |
| Austin | 2009 | Moderate | Low | Low | Low | Moderate | Moderate | Moderate |
| Bak | 2022 | Low | Low | Low | Low | Moderate | Moderate | Low |
| Baroni | 2018 | High | Low | Moderate | Low | High | Moderate | High |
| Barros-Gomes | 2017 | Low | Moderate | Low | Low | Low | Moderate | Low |
| Binder | 2019 | Low | Low | Low | Moderate | Low | Low | Low |
| Bodez | 2015 | Moderate | Low | Moderate | Moderate | Moderate | High | Moderate |
| Boynton | 2016 | Moderate | Low | Moderate | Low | Moderate | Moderate | Moderate |
| Buss | 2012 | Low | Low | Moderate | Low | Low | Low | Low |
| Cappelli | 2012 | Moderate | Low | Low | Low | Moderate | Low | Moderate |
| Cappelli | 2017 | Moderate | Low | Moderate | Low | Moderate | High | Moderate |
| Chacko | 2020 | Low | Low | Low | Low | Low | Low | Low |
| Cipriani | 2022 | Low | Low | Moderate | Low | Low | Low | Low |
| Cohen | 2022 | Low | Low | Moderate | Low | Moderate | Moderate | Moderate |
| Dongiglio | 2022 | High | Moderate | Low | High | Low | Moderate | High |
| Durante-López | 2021 | High | Low | Moderate | Moderate | Moderate | High | High |
| Escher | 2020 | Low | Low | Low | Low | Moderate | Low | Low |
| Fagot | 2021 | Low | Moderate | Moderate | Low | Moderate | Moderate | Moderate |
| Fine | 2020 | Moderate | Low | Moderate | High | Moderate | High | High |
| Fontana | 2015 | Low | Low | Moderate | Low | Moderate | Moderate | Moderate |
| Fumagalli | 2021 | Low | Moderate | High | Low | Moderate | Moderate | Moderate |
| Galat | 2015 | Moderate | High | Moderate | Moderate | Moderate | Moderate | High |
| Hamon | 2016 | High | Moderate | Moderate | Low | Moderate | Moderate | Moderate |
| Hanson | 2018 | Low | Low | Low | Low | Moderate | Moderate | Moderate |
| Hein | 2018 | High | Moderate | Moderate | Low | High | Moderate | High |
| Hu | 2015 | High | Low | Low | Low | Moderate | Moderate | Moderate |
| Hwang | 2021 | Moderate | Moderate | Low | Moderate | Moderate | Moderate | Moderate |
| Ingebrigtsen | 2024 | Moderate | Moderate | Moderate | Low | Moderate | Moderate | Moderate |
| Jakaitė | 2021 | Moderate | Low | Moderate | Low | Moderate | Moderate | Moderate |
| Kado | 2016 | Moderate | Low | Low | Moderate | Moderate | Moderate | Moderate |
| Kharoubi | 2021 | Moderate | High | Moderate | Moderate | High | Moderate | High |
| Kim | 2021 | Moderate | Low | Moderate | Low | Moderate | Moderate | Moderate |
| Klarskov | 2023 | Low | Low | Moderate | Moderate | Moderate | High | Moderate |
| Knight | 2019 | Low | Low | Low | Low | Low | Low | Low |
| Koutroumpakis | 2023 | High | Low | Low | Low | Moderate | High | High |
| Kristen | 2016 | Low | Low | Moderate | Low | High | Moderate | Moderate |
| Kristen | 2014 | Moderate | Low | Low | Low | Moderate | Low | Low |
| Ladefoged | 2023 | Moderate | Low | Low | Low | Low | Low | Low |
| Lee Chuy | 2020 | Moderate | Low | Low | Low | Low | Low | Low |
| Lee | 2014 | Low | Moderate | Moderate | Low | Low | Moderate | Moderate |
| Leedy | 2020 | High | Low | Low | Low | Moderate | Moderate | Moderate |
| Lei | 2021 | Moderate | Moderate | Low | Low | High | High | High |
| Licordari | 2022 | High | Low | Moderate | Moderate | Moderate | High | High |
| Lin | 2018 | Moderate | Low | High | Low | Moderate | High | High |
| Liu | 2014 | Moderate | Low | Low | Low | Moderate | Moderate | Moderate |
| Liu | 2017 | Moderate | Low | Low | Low | Moderate | Low | Moderate |
| Liu | 2022 | Moderate | Low | Low | Low | Moderate | Moderate | Moderate |
| Longhi | 2015 | Moderate | High | Moderate | Low | Moderate | Moderate | Moderate |
| Maccallini | 2024 | Low | Low | Moderate | Moderate | Moderate | Moderate | Moderate |
| Migrino | 2009 | High | Low | Low | Low | Moderate | High | High |
| Mohty | 2017 | Low | Moderate | Moderate | Low | Moderate | High | Moderate |
| Ozbay | 2024 | Low | Low | Low | Moderate | Low | Low | Low |
| Palmiero | 2023 | Moderate | Low | Low | Moderate | Moderate | Moderate | Moderate |
| Pericet-Rodríguez | 2022 | High | Low | Moderate | Low | Moderate | Moderate | Moderate |
| Pislaru | 2019 | High | Low | Low | Moderate | Moderate | High | High |
| Porcari | 2022 | Moderate | High | Moderate | Low | Moderate | High | High |
| Porcari | 2024 | Low | Low | Low | Low | Low | Low | Low |
| Porcari | 2023 | Low | Low | Moderate | Low | Low | Low | Low |
| Pun | 2018 | Moderate | Low | Low | Low | High | High | High |
| Quarta | 2014 | Low | Low | Low | Low | Low | Moderate | Low |
| Rapezzi | 2011 | Moderate | Low | Moderate | Moderate | Moderate | Moderate | Moderate |
| Rosenblum | 2018 | Low | Low | Moderate | Low | Moderate | Low | Low |
| Rosengren | 2024 | High | Low | Moderate | Low | Moderate | Moderate | Moderate |
| Senapati | 2016 | Moderate | Low | High | Low | High | High | High |
| Siddiqi | 2022 | Low | Moderate | Moderate | Low | Moderate | Moderate | Moderate |
| Singulane | 2024 | Low | Low | Low | Low | Low | Moderate | Low |
| Sperry | 2016 | Low | Moderate | Low | Low | Low | Low | Low |
| Szczygieł | 2017 | High | Low | Moderate | Low | Moderate | High | High |
| Tahir | 2019 | Low | Low | Low | Low | Moderate | Moderate | Moderate |
| Takashio | 2023 | Low | Low | Moderate | Moderate | Low | High | Moderate |
| Tan | 2022 | Moderate | Moderate | Moderate | Low | Low | Low | Moderate |
| Tanaka | 2013 | Low | Low | Low | Low | Moderate | Moderate | Low |
| Tendler | 2015 | Moderate | Low | Low | Low | Moderate | Moderate | Moderate |
| Ternacle | 2016 | Low | Low | Low | Moderate | Low | Moderate | Low |
| Tjahadi | 2022 | Moderate | Low | Moderate | Low | Moderate | High | Moderate |
| Tomasoni | 2023 | Low | Low | Moderate | Moderate | Low | Moderate | Moderate |
| Tomasoni | 2022 | Low | Low | Moderate | Low | Low | Moderate | Low |
| Usuku | 2022 | Low | Low | Moderate | Moderate | Low | Moderate | Moderate |
| Usuku | 2023 | Moderate | Low | Moderate | Low | Low | Moderate | Moderate |
| Usuku | 2024 | Moderate | Low | Moderate | Low | Moderate | Moderate | Moderate |
| Uzan | 2018 | Low | Low | Low | Low | Moderate | Moderate | Moderate |
| Vranian | 2018 | High | Low | Moderate | Moderate | Low | Moderate | Moderate |
| Witteles | 2024 | Low | Low | Low | Low | Low | Low | Low |
| Wu | 2023 | Moderate | Low | Moderate | Low | Moderate | Moderate | Moderate |
| Wu | 2021 | Low | High | Moderate | Low | Moderate | Moderate | Moderate |
| Yamada | 2020 | Low | Low | Moderate | Moderate | Moderate | Moderate | Moderate |
| Yang | 2023 | Moderate | High | Moderate | Low | Low | Moderate | Moderate |
| Zampieri | 2022 | Low | Low | Low | Low | Moderate | Moderate | Low |
| Zaroui | 2024 | Low | Low | Moderate | Low | Moderate | High | High |
| Zhang | 2023 | Moderate | Low | Moderate | Low | Moderate | Moderate | Moderate |
| Zhao | 2016 | Low | Low | Low | High | Moderate | Moderate | Moderate |

Table S3: Summary of pooled hazard ratios and associated 95% confidence intervals, statistical heterogeneity and small-study effect assessment for individual echocardiographic parameters and clinical outcomes.

| **Echocardiographic parameter** | **k* (n^†^)** | **Pooled HR (95% CI)** | **P for overall effect** | **I2** | **P for small study effects (Egger test)** |
| --- | --- | --- | --- | --- | --- |
| **LV-GLS**  All-cause mortality  AL  ATTR  Cardiovascular mortality  MACE  AL  ATTR | 35 (7746)  20 (2429)  7 (4413)  4 (304)  13 (1420)  1 (60)  6 (611) | 1.10 (1.08 – 1.12)  1.11 (1.07 – 1.14)  1.08 (1.04 – 1.12)  1.22 (1.03 – 1.43)  1.14 (1.07 – 1.22)  k<3  1.12 (1.03 – 1.21) | <0.001  <0.001  0.003  0.03  <0.001  k<3  0.02 | 51%  51%  56%  36%  73%  k<3  34% | <0.001  NA  NA  k<10  0.01  NA  NA |
| **RV-FWS**  All-cause mortality  AL  ATTR  Cardiovascular mortality  MACE | 10 (877)  8 (653)  1 (131)  2 (166)  5 (378) | 1.11 (1.03 – 1.20)  1.13 (1.02 – 1.25)  k<3  k<3  1.10 (1.05 – 1.15) | 0.009  0.02  k<3  k<3  0.005 | 75%  79%  k<3  k<3  0% | 0.02  NA  NA  k<10  k<10 |
| **LVEF**  All-cause mortality  AL  ATTR  Cardiovascular mortality  MACE  AL  ATTR | 53 (9885)  26 (3244)  12 (4846)  6 (715)  14 (1706)  1 (60)  7 (667) | 0.98 (0.98 – 0.98)  0.98 (0.97 – 0.98)  0.98 (0.97 – 1.00)  0.97 (0.95 – 0.99)  0.97 (0.96 – 0.99)  k<3  0.97 (0.94 – 0.99) | <0.001  <0.001  0.002  0.005  0.004  k<3  0.007 | 42%  19%  57%  0%  62%  k<3  45% | 0.12  NA  NA  k<10  0.24  NA  NA |
| **TAPSE**  All-cause mortality  AL  ATTR  Cardiovascular mortality  MACE | 18 (3042)  10 (829)  3 (1613)  4 (572)  4 (487) | 0.94 (0.93 – 0.95)  0.93 (0.91 – 0.96)  0.94 (0.93 – 0.95)  0.91 (0.90 – 0.91)  0.93 (0.90 – 0.97) | <0.001  <0.001  <0.001  <0.001  0.008 | 0%  0%  0%  0%  0% | 0.55  NA  NA  k<10  k<10 |
| **IVSd**  All-cause mortality  AL  ATTR  Cardiovascular mortality  MACE | 28 (6003)  15 (1668)  6 (3682)  4 (568)  6 (616) | 1.04 (1.00 – 1.07)  1.07 (1.02 – 1.13)  1.02 (0.99 – 1.05)  1.10 (0.85 – 1.42)  1.03 (0.98 – 1.09) | 0.04  0.01  0.15  0.32  0.16 | 59%  57%  0%  54%  0% | 0.63  NA  NA  k<10  k<10 |
| **LVMi**  All-cause mortality  AL  ATTR  Cardiovascular mortality  MACE | 23 (3778)  15 (1611)  3 (1722)  4 (558)  3 (386) | 1.000 (0.998 – 1.002)  1.001 (0.998 – 1.004)  0.999 (0.981 – 1.017)  1.00 (0.99 – 1.02)  1.00 (0.99 – 1.01) | 0.88  0.54  0.85  0.32  0.57 | 56%  66%  67%  44%  0% | 0.83  NA  NA  k<10  k<10 |
| **E/e’**  All-cause mortality  AL  ATTR  Cardiovascular mortality  MACE | 37 (6376)  21 (2054)  6 (3151)  6 (715)  8 (819) | 1.02 (1.02 – 1.03)  1.02 (1.01 – 1.03)  1.02 (1.01 – 1.03)  1.04 (1.01 – 1.08)  1.04 (1.02 – 1.06) | <0.001  <0.001  0.009  0.02  <0.001 | 20%  29%  23%  43%  1% | 0.24  NA  NA  k<10  k<10 |

*’k’ denotes the number of studies

†’n’ denotes the number of patients

Table S4: Results of random-effects meta-regression assessing the strength of observed associations between echocardiographic measures and all-cause mortality adjusting for baseline age, LVEF, eGFR, NT-proBNP, proportion of male participants and statistical adjustment (unadjusted vs adjusted analysis)

|  | **Age** | **Gender** | **eGFR** | **NT-proBNP** | **LVEF** | **Adjustment** |
| --- | --- | --- | --- | --- | --- | --- |
| **LVGLS** | AL = 0.0127  ATTR k<10 | AL = 0.1152  ATTR k<10 | 0.1198 | 0.5807 | 0.6960 | 0.0261 |
| **RVFWS** | k<10 | k<10 | k<10 | k<10 | 0.7910 | 0.0094 |
| **LVEF** | AL = 0.5079  ATTR = 0.7597 | AL = 0.5206  ATTR = 0.4086 | 0.0816 | 0.0981 | N/A | 0.2254 |
| **TAPSE** | AL = 0.6255  ATTR k<10 | AL = 0.1744  ATTR k<10 | 0.7947 | 0.5348 | 0.4989 | 0.4453 |
| **LVMi** | AL = 0.1554  ATTR k<10 | AL = 0.7274  ATTR k<10 | 0.8147 | 0.1085 | 0.2683 | 0.9339 |
| **IVSd** | AL = 0.9525  ATTR k<10 | AL = 0.7105  ATTR k<10 | 0.8402 | 0.7727 | 0.0613 | 0.7218 |
| **E/e’** | AL = 0.0922  ATTR k<10 | AL = 0.1288  ATTR k<10 | 0.8681 | 0.8126 | 0.9413 | 0.9694 |

Figure S1: PRISMA flow-chart outlining the study selection process


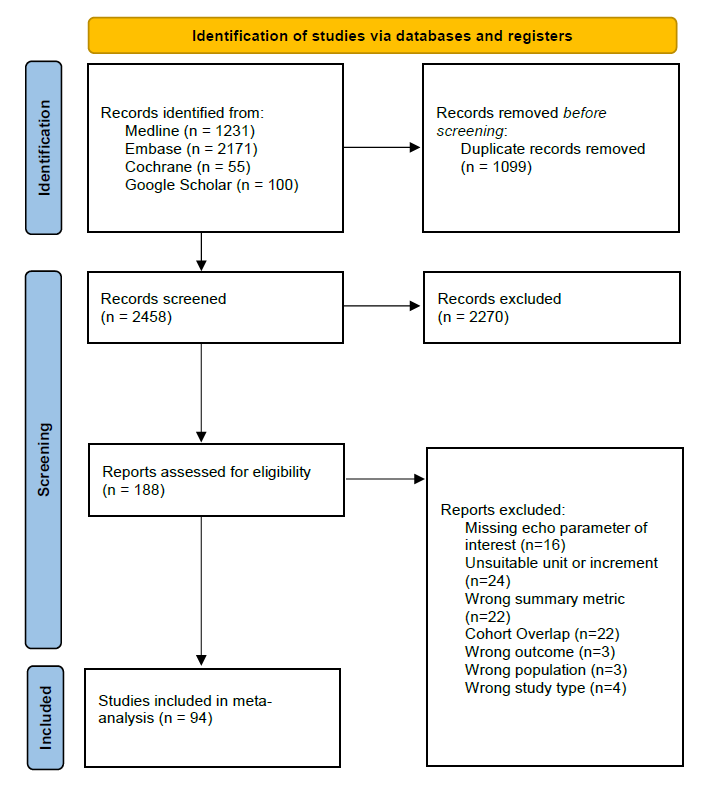


Figure S2: Random-effects meta-analysis using the HKSJ method summarizing the association between all-cause mortality and LV-GLS, per 1% decrement, with subgroup analysis according to strain software used (EchoPAC, TomTec Imaging, others)


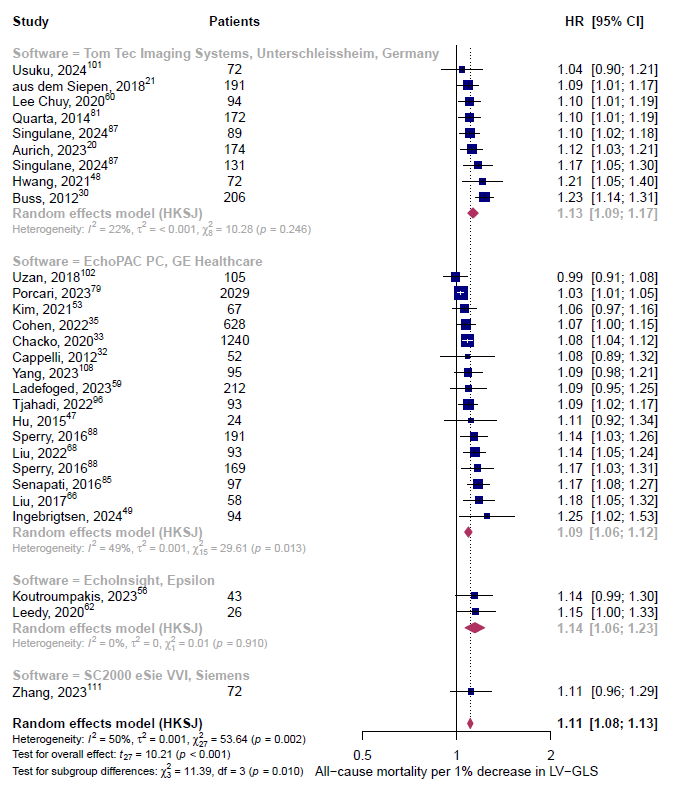


Figure S3: Random-effects meta-analysis using the HKSJ method summarizing the association between cardiovascular mortality and LV-GLS, per 1% decrement


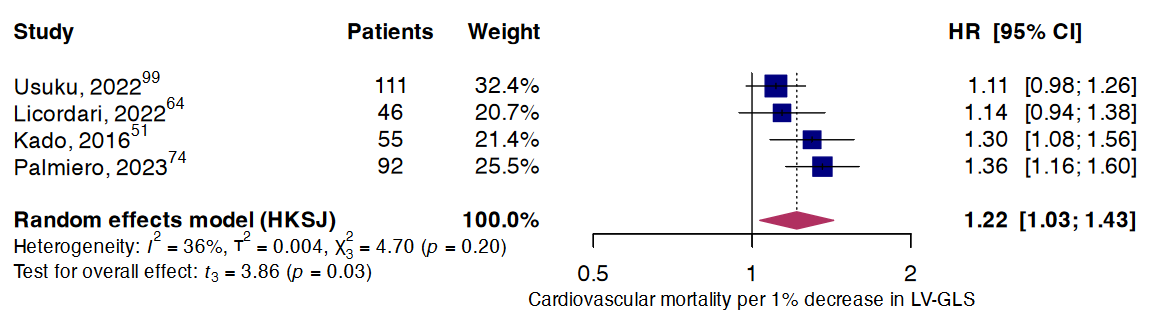


Figure S4: Random-effects meta-analysis using the HKSJ method summarizing the association between MACE and LV-GLS, per 1% decrement, including subgroup analysis for AL amyloidosis, ATTR amyloidosis and cohorts with mixed etiologies.


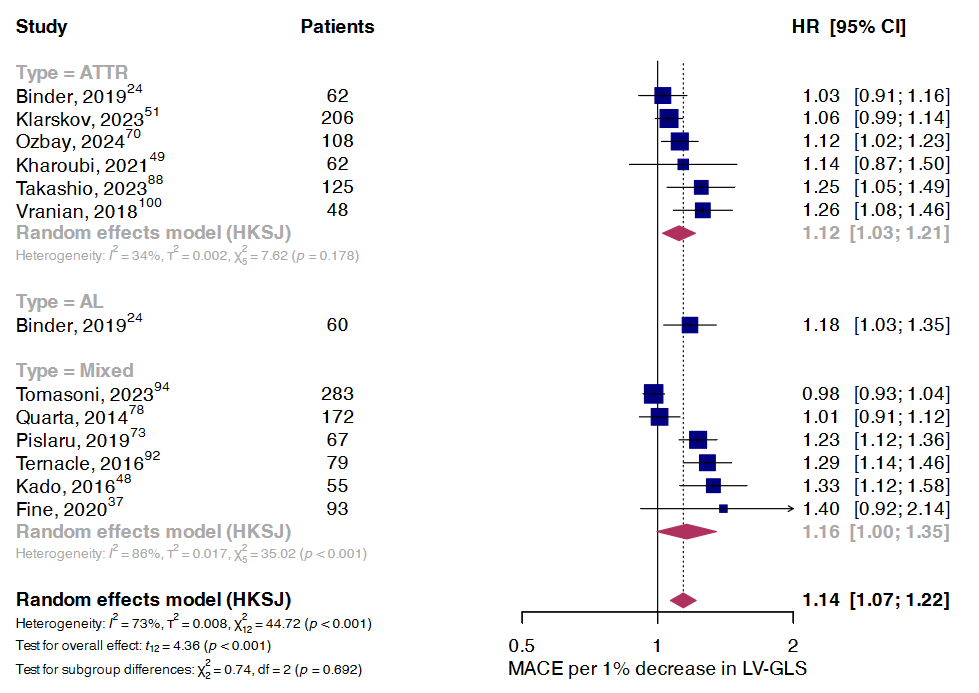


Figure S5: Random-effects meta-analysis using the HKSJ method summarizing the association between all-cause mortality and RV-FWS, per 1% decrement.


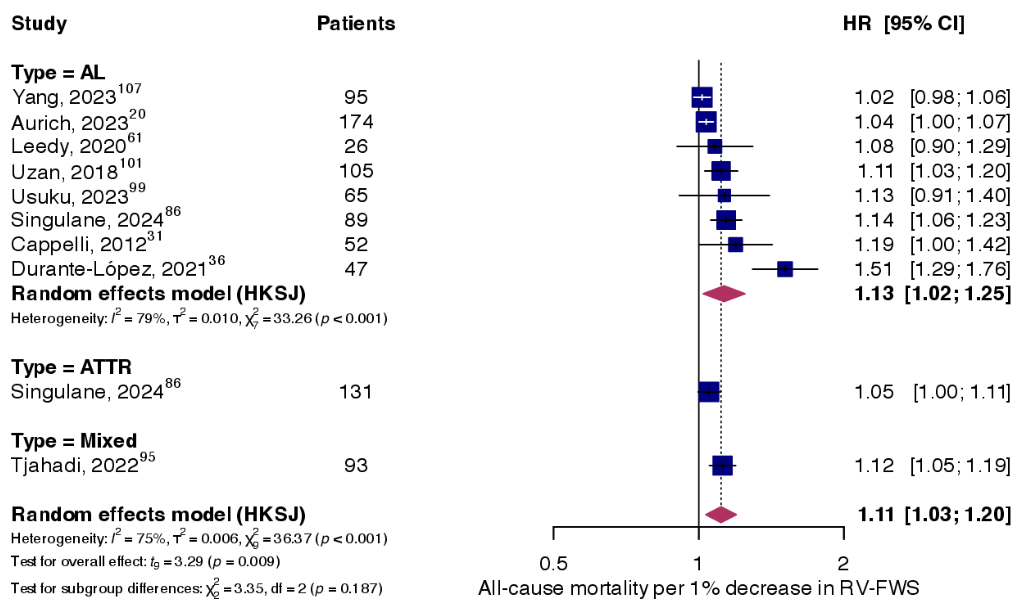


Figure S6: Random-effects meta-analysis using the HKSJ method summarizing the association between MACE and RV-FWS, per 1% decrement.


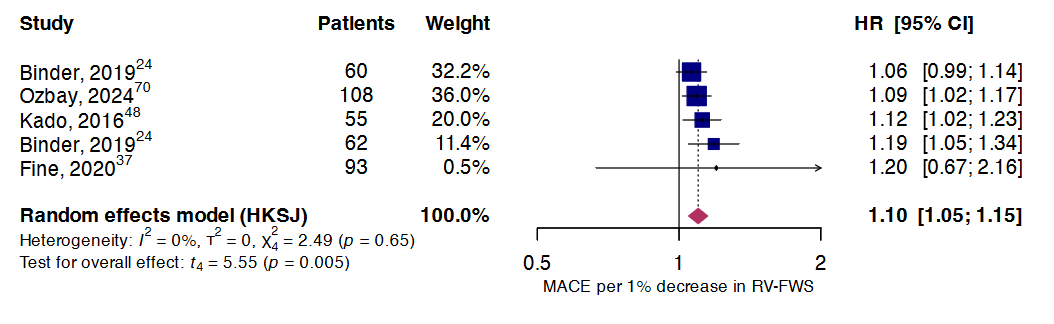


Figure S7: Random-effects meta-analysis using the HKSJ method summarizing the association between cardiovascular mortality and LVEF, per 1% increase.


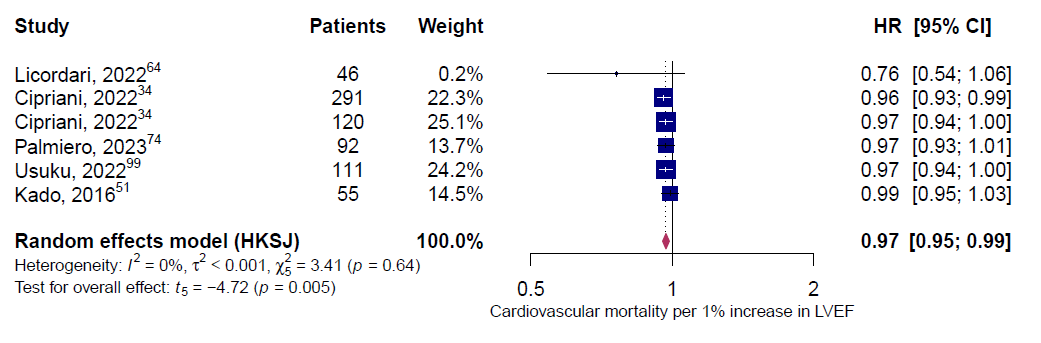


Figure S8: Random-effects meta-analysis using the HKSJ method summarizing the association between MACE and LVEF, per 1% increase, with subgroup analysis for AL amyloidosis, ATTR amyloidosis and cohorts with mixed etiologies.


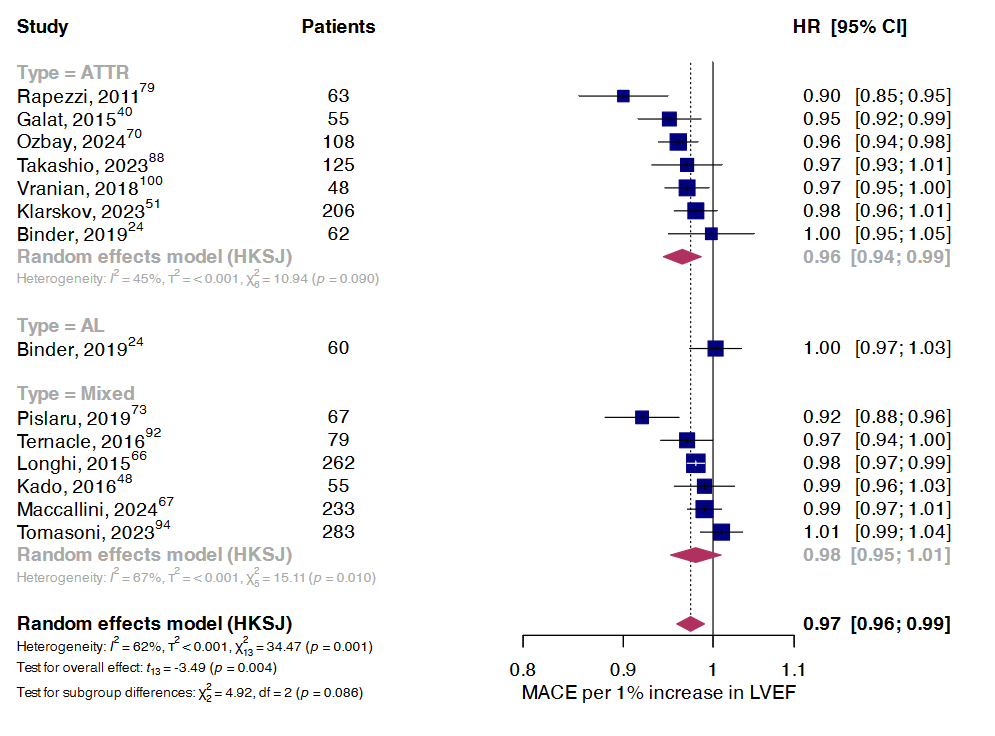


Figure S9: Random-effects meta-analysis using the HKSJ method summarizing the association between cardiovascular mortality and TAPSE, per 1mm increase.


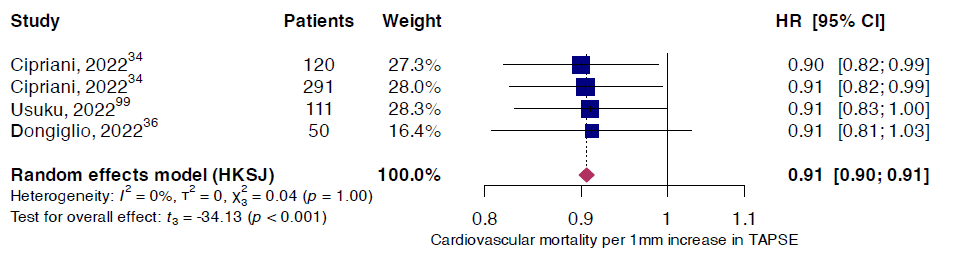


Figure S10: Random-effects meta-analysis using the HKSJ method summarizing the association between MACE and TAPSE, per 1mm increase.


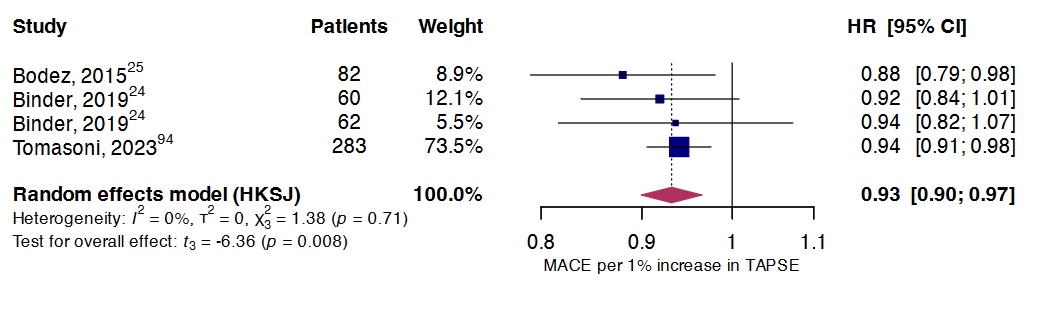


Figure S11: Random-effects meta-analysis using the HKSJ method summarizing the association between cardiovascular mortality and IVSd, per 1mm increase.


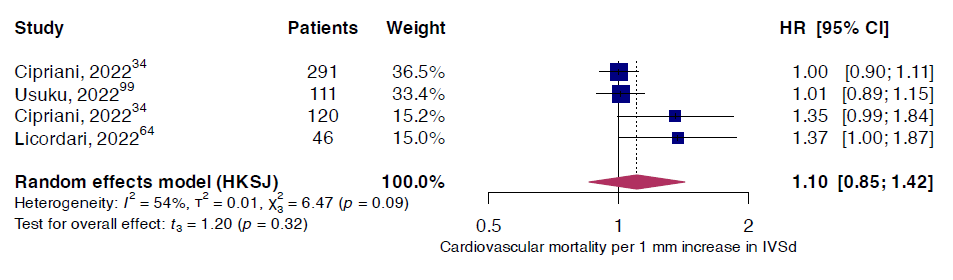


Figure S12: Random-effects meta-analysis using the HKSJ method summarizing the association between MACE and IVSd, per 1mm increase.


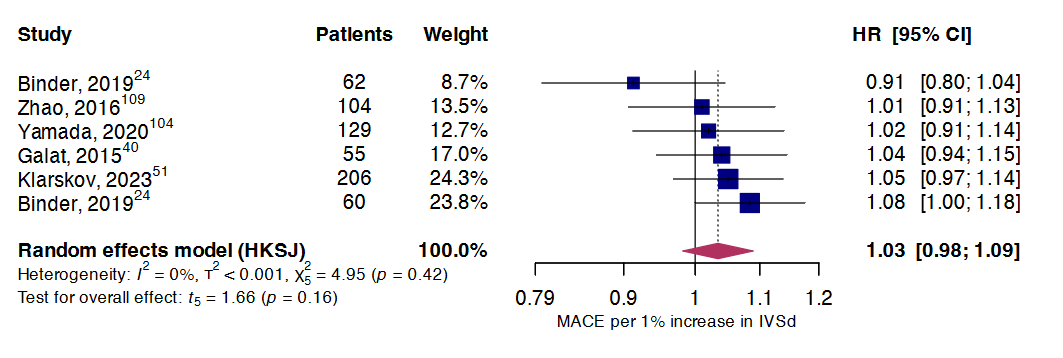


Figure S13: Random-effects meta-analysis using the HKSJ method summarizing the association between cardiovascular mortality and LVMi, per 1g/m2 increase.


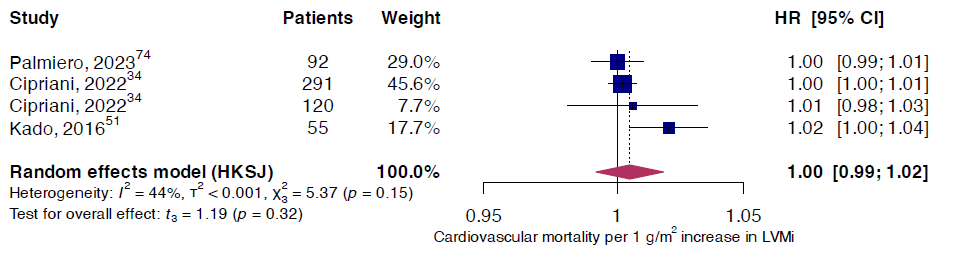


Figure S14: Random-effects meta-analysis using the HKSJ method summarizing the association between MACE and LVMi, per 1g/m2 increase.


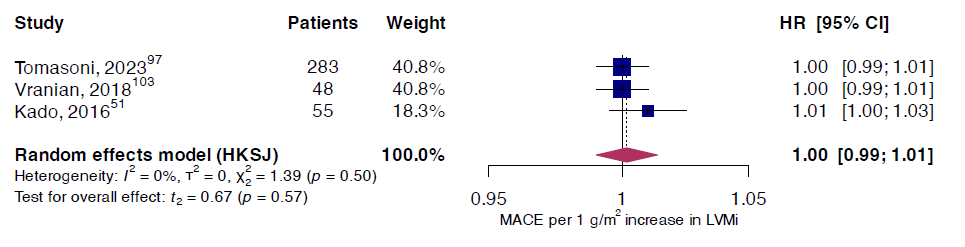


Figure S15: Random-effects meta-analysis using the HKSJ method summarizing the association between cardiovascular mortality and the E/e’ ratio, per 1 unit increase.


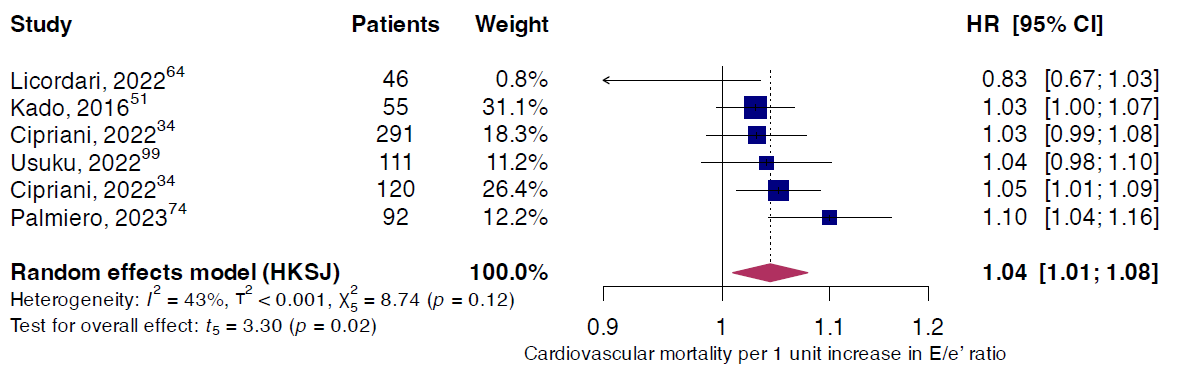


Figure S16: Random-effects meta-analysis using the HKSJ method summarizing the association between MACE and the E/e’ ratio, per 1 unit increase.


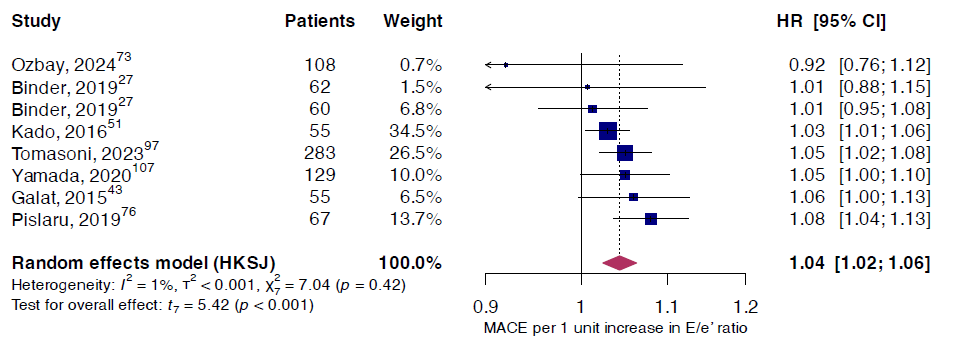


Appendix 1: Search strategy

**Limitations**

-

**Key words**

A1: amyloidosis, cardiac amyloidosis (CA), transthyretin (TTR) amyloidosis, amyloid light chain (AL) amyloidosis

A2: echocardiography, echo, left ventricular ejection fraction (LVEF), ejection fraction, left/right ventricular function, left/right ventricular dysfunction, diastolic function, strain, global longitudinal strain (GLS), right ventricular free wall strain, left ventricular mass, deceleration time

A3: prognosis, mortality, all-cause mortality, death, all-cause death, survival, adverse outcomes, adverse events, major adverse cardiovascular events (MACE)

**Search strings**

A1 AND A2 AND A3

**PubMed or Ovid Medline**

11.7.24, 1231

| (  "Amyloidosis"[Mesh] OR  Amyloidosis[tiab]  )  AND | A1 Amyloidosis  Eng: "cardiac Amyloidosis"[tiab] OR  ((light-chain[tiab] OR AL[tiab] OR Transthyretin[tiab] OR ATTR[tiab] OR TTR[tiab]) **AND** Amyloidosis[tiab]) |
| --- | --- |
| (  "Echocardiography"[Mesh] OR  Echocardiograph*[tiab] OR  Echo[tiab] OR  "Ventricular Dysfunction, Left"[Mesh] OR  "Ventricular Function, Left"[Mesh] OR  "left ventric*"[tiab] OR  "lv mass"[tiab] OR  "ejection fraction"[tiab] OR  LVEF[tiab] OR  "Ventricular Dysfunction, Right"[Mesh] OR  "Ventricular Function, Right"[Mesh] OR  "right ventric*"[tiab] OR  "Global Longitudinal Strain"[Mesh] OR  GLS[tiab] OR  Strain[tiab] OR  "deceleration time"[tiab] OR  diastolic[tiab] OR  "tricuspid annular plane systolic excursion"[tiab:~2] OR  TAPSE[tiab]  )  AND | A2 echocardiography, … |
| (  "Prognosis"[Mesh:NoExp] OR  Prognos*[tiab] OR  "Mortality"[Mesh] OR  "mortality"[Subheading] OR  Mortality[tiab] OR  Death*[tiab] OR  survival[tiab] OR  "adverse effects"[Subheading] OR  Adverse[tiab] OR  "Side effect*"[tiab]  ) | A3  "Survival Rate"[Mesh] < Mortality[Mesh], therefore not necessary |

**Embase**

11.7.24, 2171 (eng: 1773)

| (  "Amyloidosis"/exp OR  Amyloidosis:ti,ab,kw  )  AND | Eng:  'AL amyloidosis'/exp OR  'ATTR amyloidosis'/exp OR  'heart amyloidosis'/exp OR  "cardiac Amyloidosis":ti,ab,kw OR  ((light-chain OR AL OR Transthyretin OR ATTR OR TTR) **AND** Amyloidosis):ti,ab,kw |
| --- | --- |
| (  "Echocardiography"/exp OR  Echocardiograph*:ti,ab,kw OR  Echo:ti,ab,kw OR  'heart left ventricle failure'/exp OR  'heart left ventricle function'/exp OR  "left ventric*":ti,ab,kw OR  "lv mass":ti,ab,kw OR  "ejection fraction":ti,ab,kw OR  LVEF:ti,ab,kw OR  'heart right ventricle function'/exp OR  'heart right ventricle failure'/exp OR  "right ventric*":ti,ab,kw OR  "Global Longitudinal Strain"/exp OR  GLS:ti,ab,kw OR  Strain:ti,ab,kw OR  "deceleration time":ti,ab,kw OR  diastolic:ti,ab,kw OR  (tricuspid NEAR/3 annular NEAR/3 plane NEAR/3 systolic NEAR/3 excursion):ti,ab,kw OR  TAPSE:ti,ab,kw  )  AND |  |
| (  "Prognosis"/exp OR  Prognos*:ti,ab,kw OR  "Mortality"/exp OR  Mortality:ti,ab,kw OR  Death*:ti,ab,kw OR  'survival rate'/exp OR  survival:ti,ab,kw OR  "side effect"**:lnk** OR  "side effect*":ti,ab,kw OR  Adverse:ti,ab,kw  ) | :lnk = subheading, see [refhunter](https://refhunter.org/database_sheets/embase-via-elsevier/) |
| NOT "conference abstract"/it |  |

**Cochrane Library**

11.7.24, 55

| (  Amyloidosis:ti,ab,kw  )  AND |
| --- |
| (  Echocardiograph*:ti,ab,kw OR  Echo:ti,ab,kw OR  left NEXT ventric*:ti,ab,kw OR  "lv mass":ti,ab,kw OR  "ejection fraction":ti,ab,kw OR  LVEF:ti,ab,kw OR  right NEXT ventric*:ti,ab,kw OR  GLS:ti,ab,kw OR  Strain:ti,ab,kw OR  "deceleration time":ti,ab,kw OR  diastolic:ti,ab,kw OR  (tricuspid NEAR/3 annular NEAR/3 plane NEAR/3 systolic NEAR/3 excursion):ti,ab,kw OR  TAPSE:ti,ab,kw  )  AND |
| (  Prognos*:ti,ab,kw OR  Mortality:ti,ab,kw OR  Death*:ti,ab,kw OR  survival:ti,ab,kw OR  side NEXT effect*:ti,ab,kw OR  Adverse:ti,ab,kw  ) |
| NOT "conference abstract":kw |

**Google Scholar**

11.7.24, Top 100 via Publish or Perish Software

| (Amyloidosis) AND (Echocardiography OR Echo OR "left ventricle" OR "left ventricular" OR "lv mass" OR "ejection fraction" OR LVEF OR "right ventricle" OR "right ventricular" OR GLS OR Strain OR "deceleration time" OR diastolic OR "tricuspid annular plane systolic excursion" OR TAPSE) AND (Prognosis OR Mortality OR Death OR survival OR "side effect" OR Adverse) |
| --- |

**Deletion of duplicates**

1099 duplicate records were deleted through semi-automated detection of duplicates in Endnote.

Appendix 2: Mixed-effects meta-regression bubble plots evaluating baseline patient age, proportion of male participants, eGFR, NT-proBNP, LVEF and statistical adjustment (unadjusted vs adjusted analysis) as potential effect modifiers for the associations between echocardiographic variables and all-cause mortality


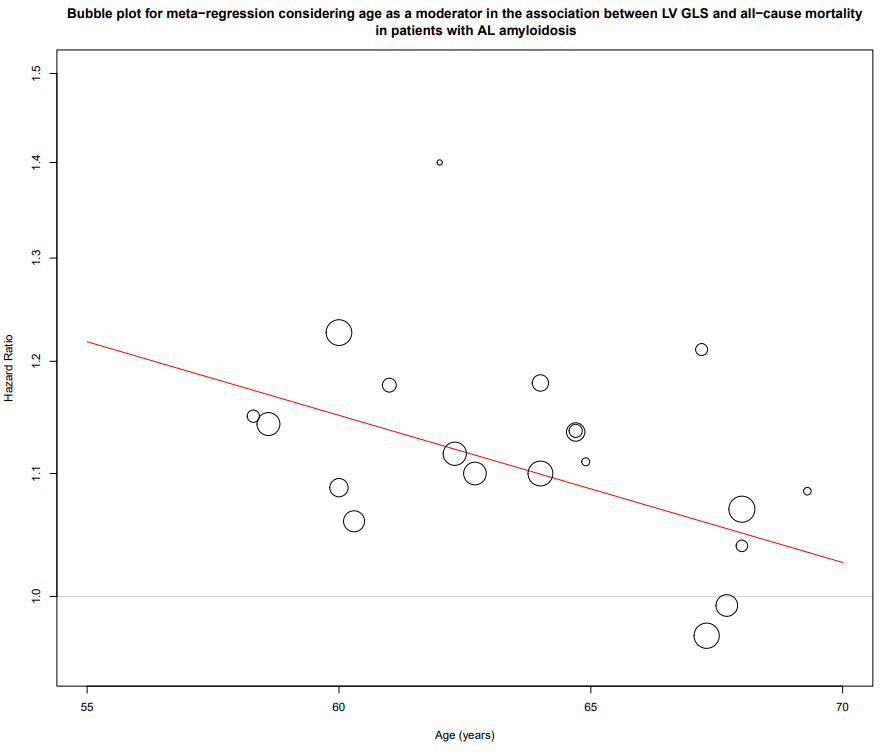


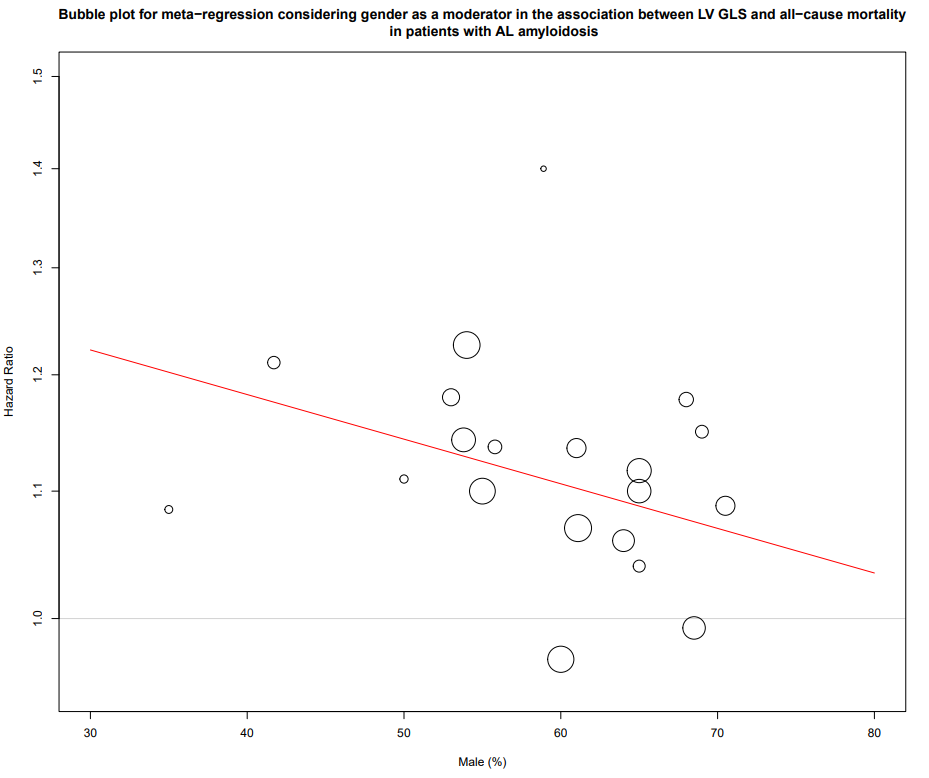


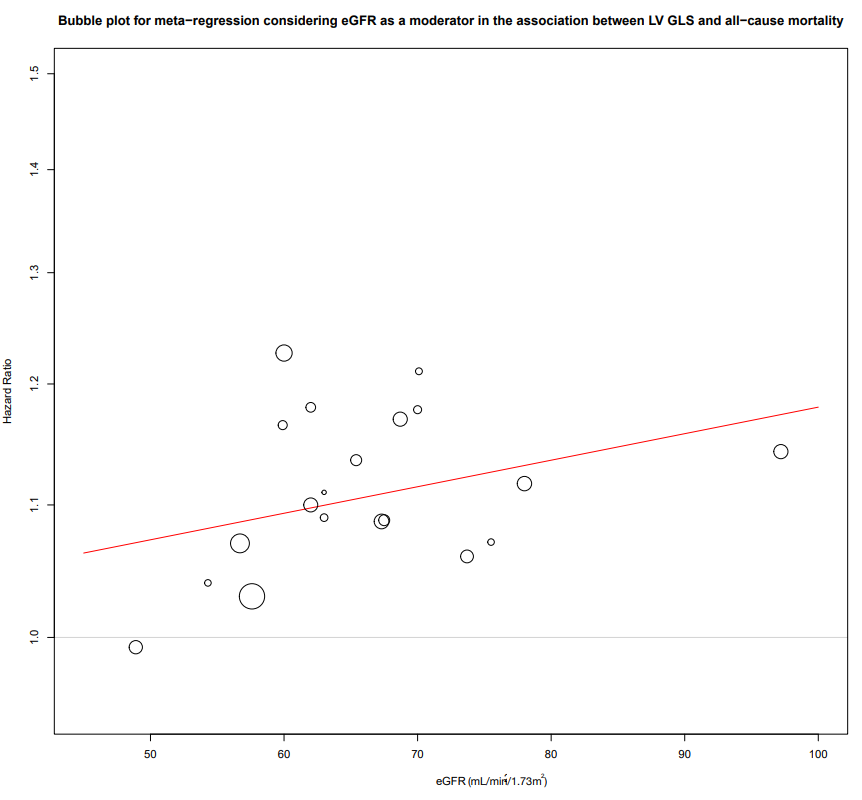


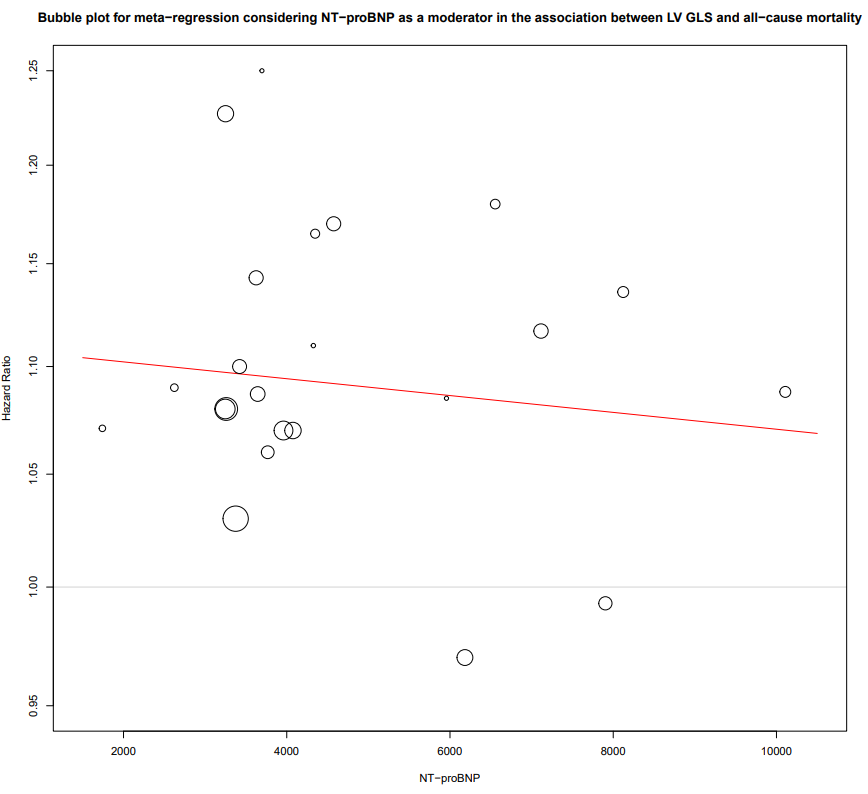


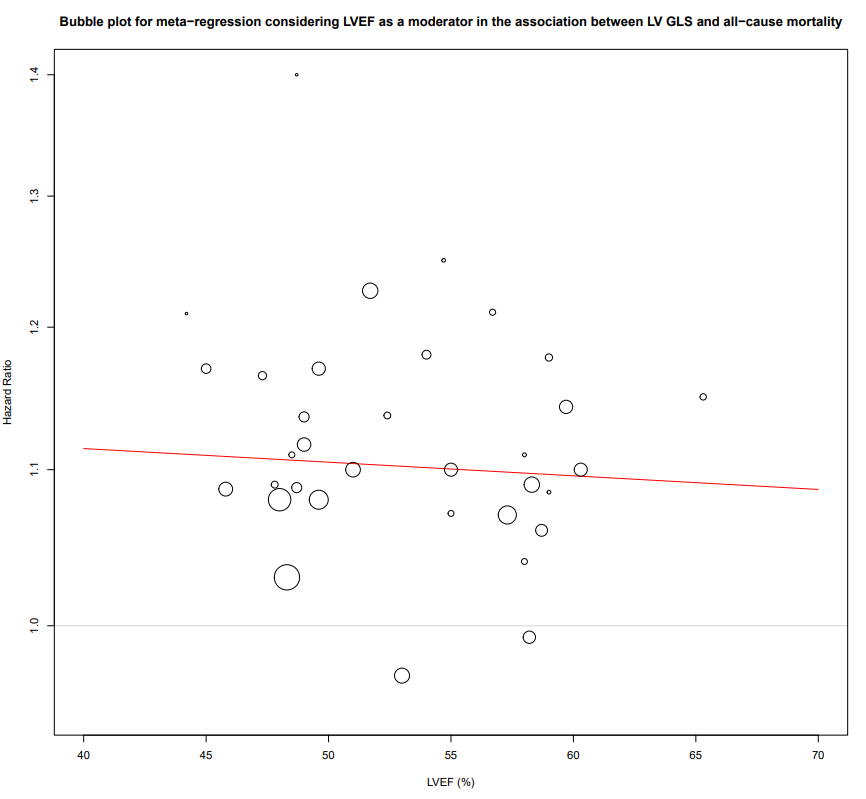


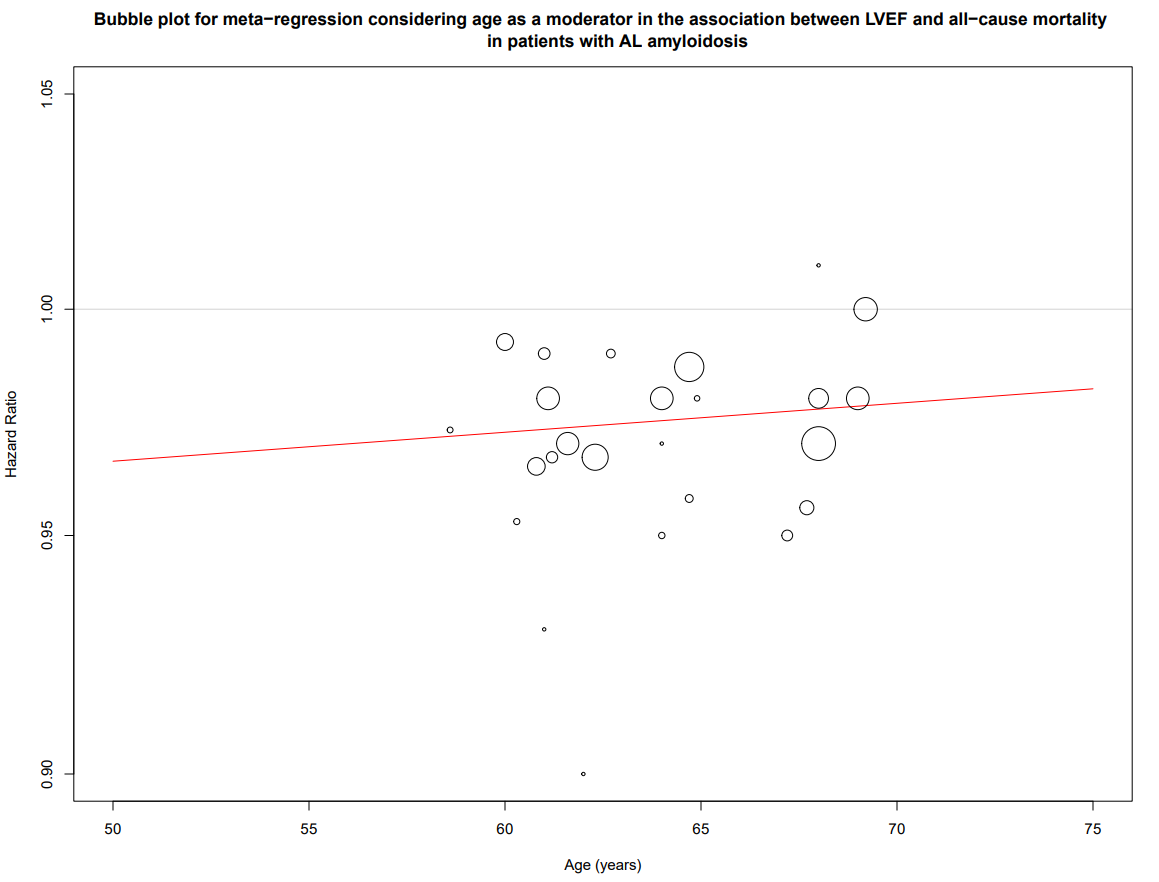


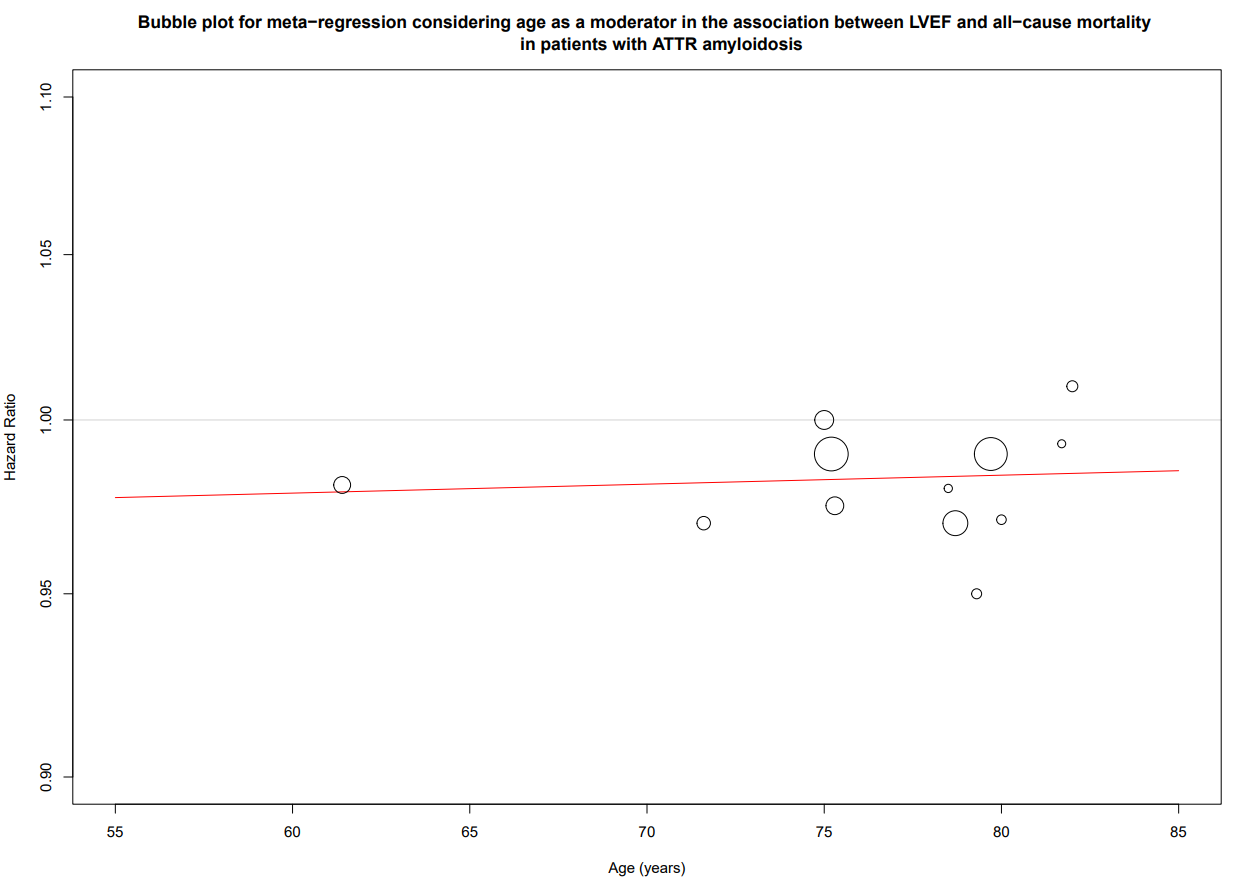


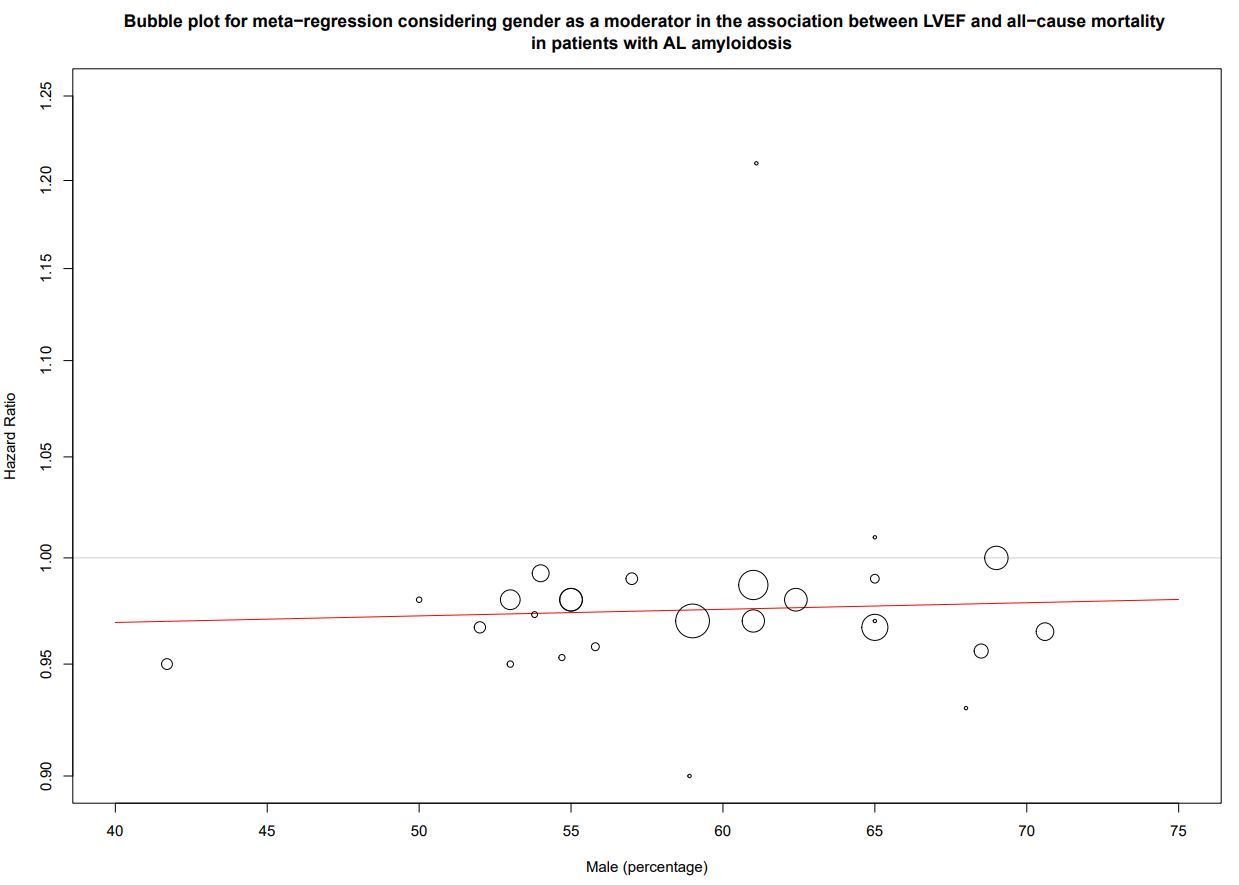


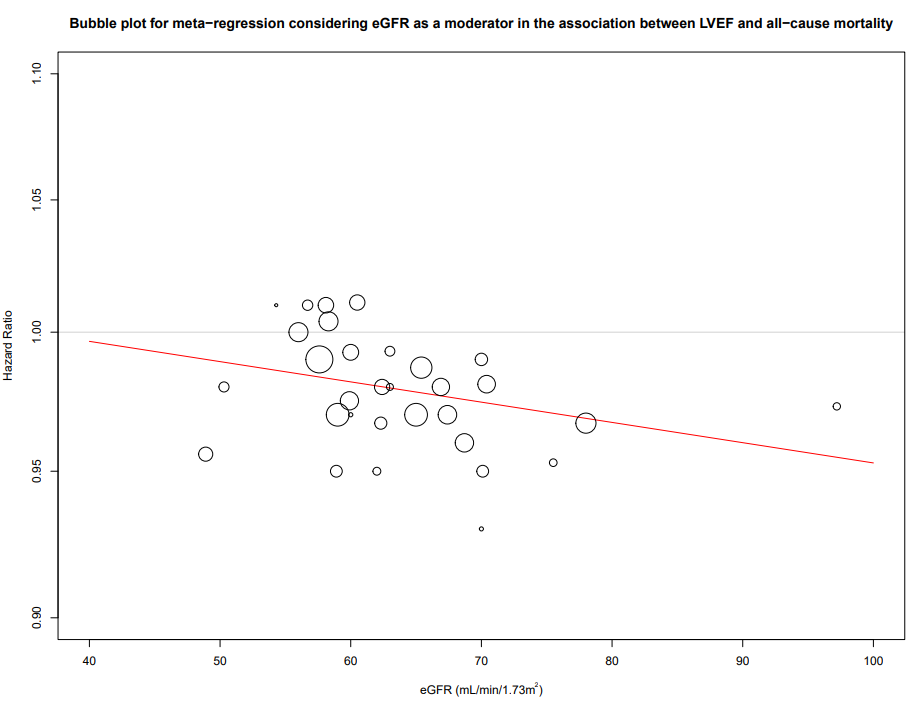


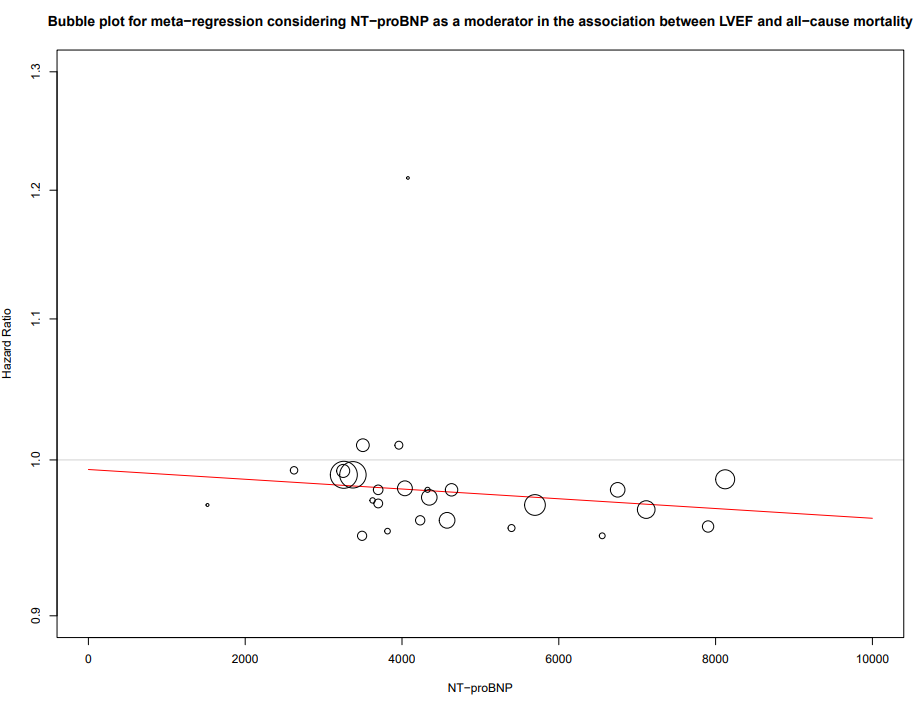


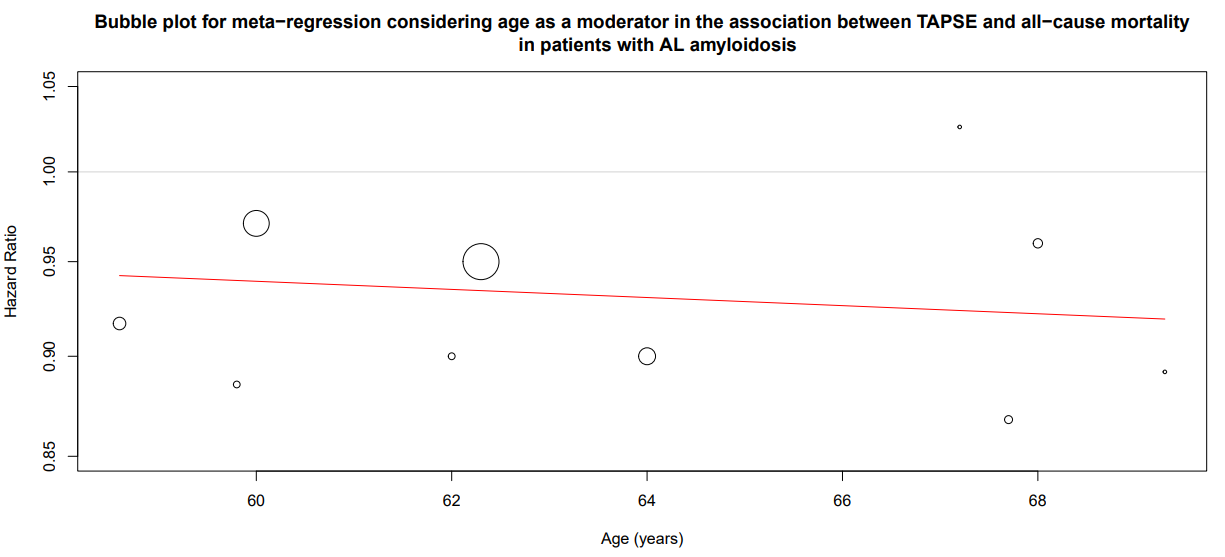


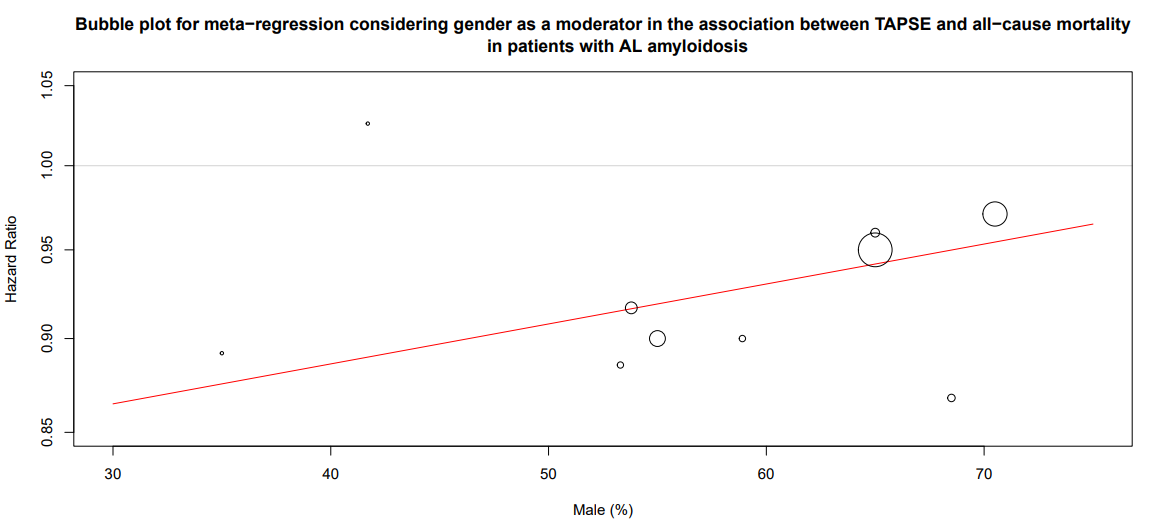


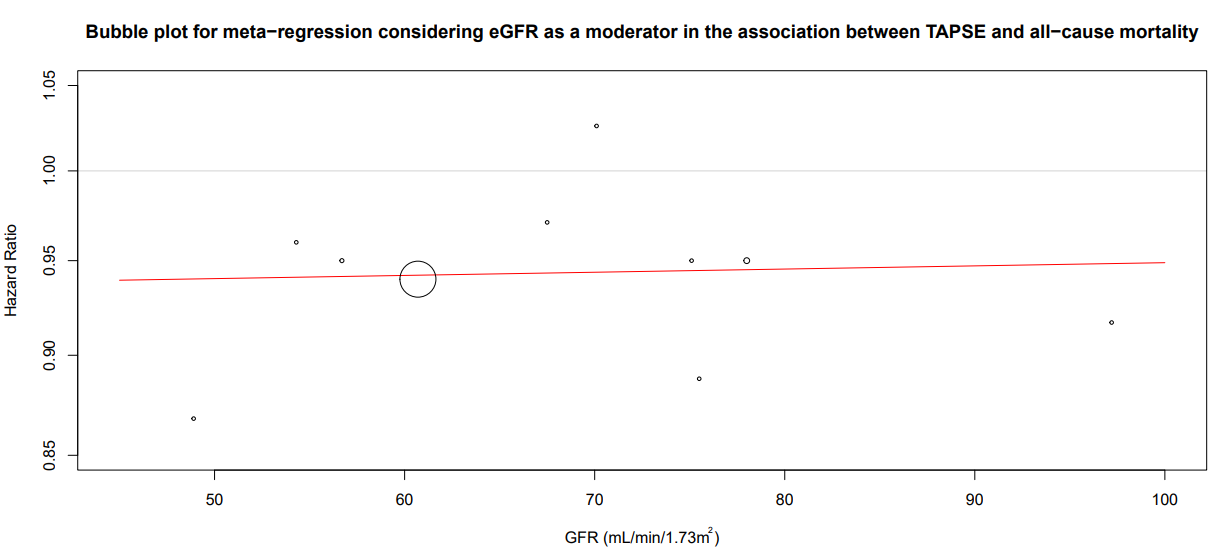


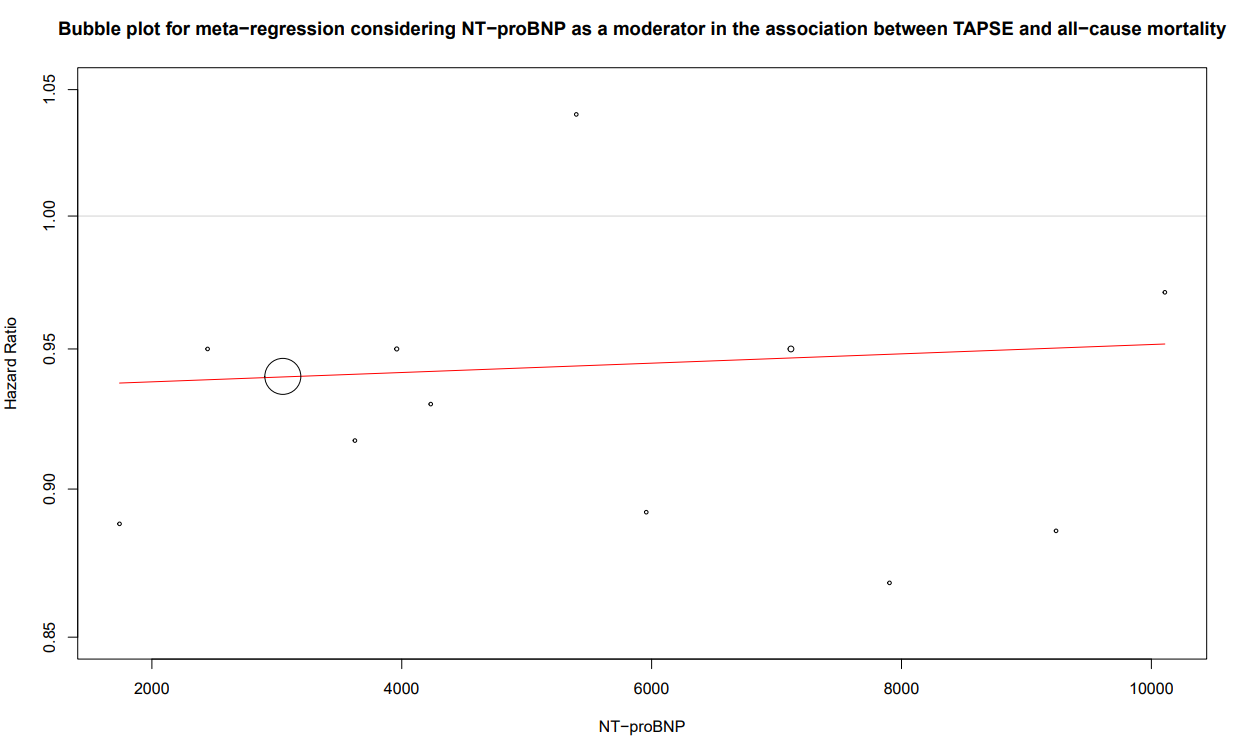


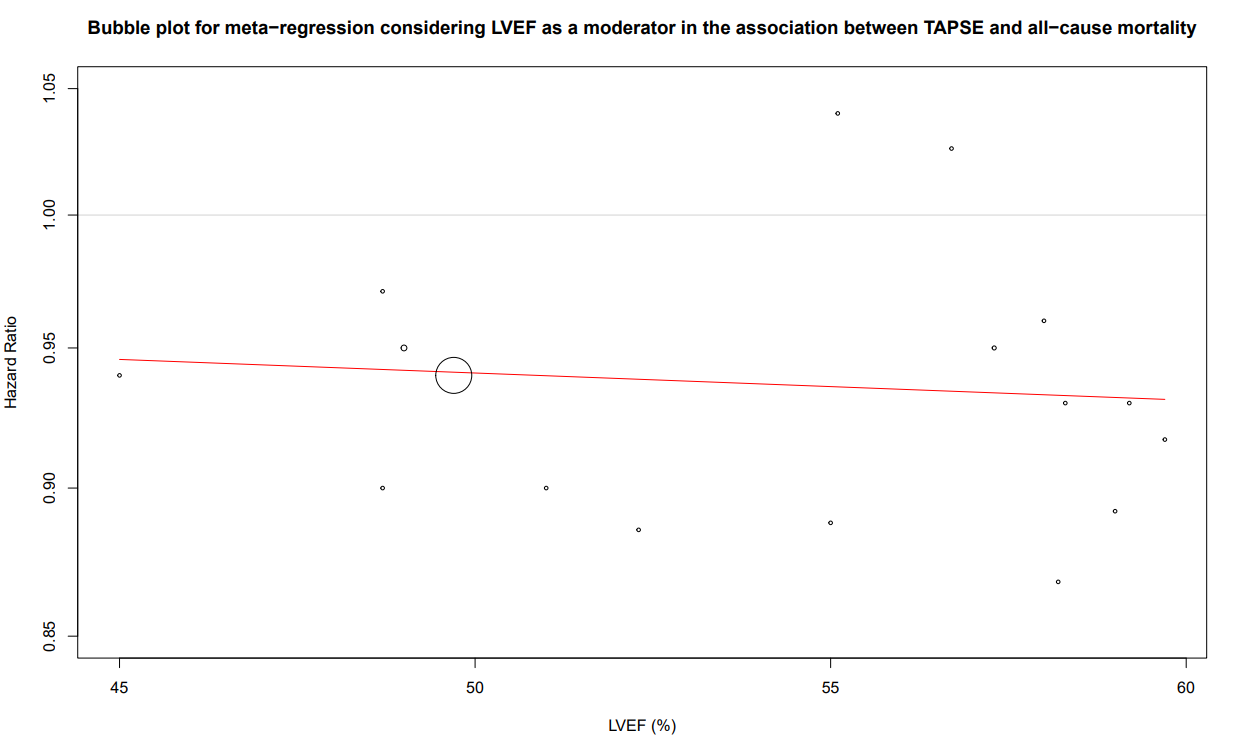


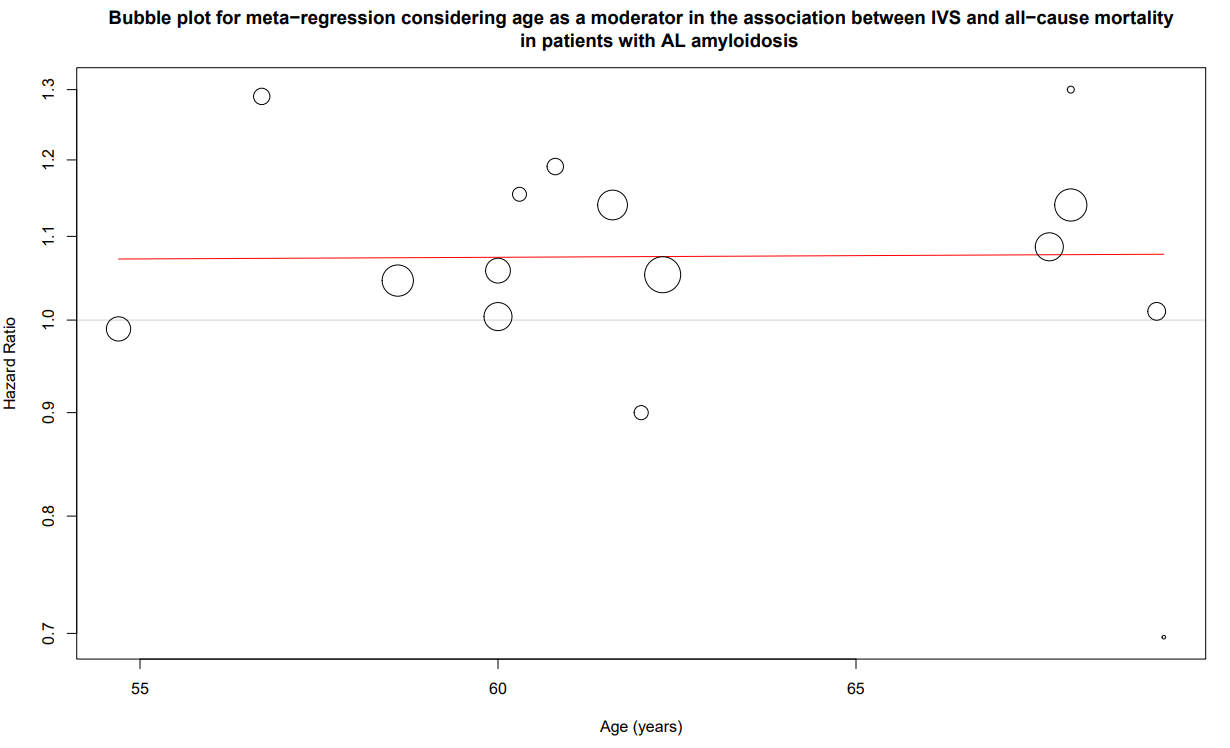


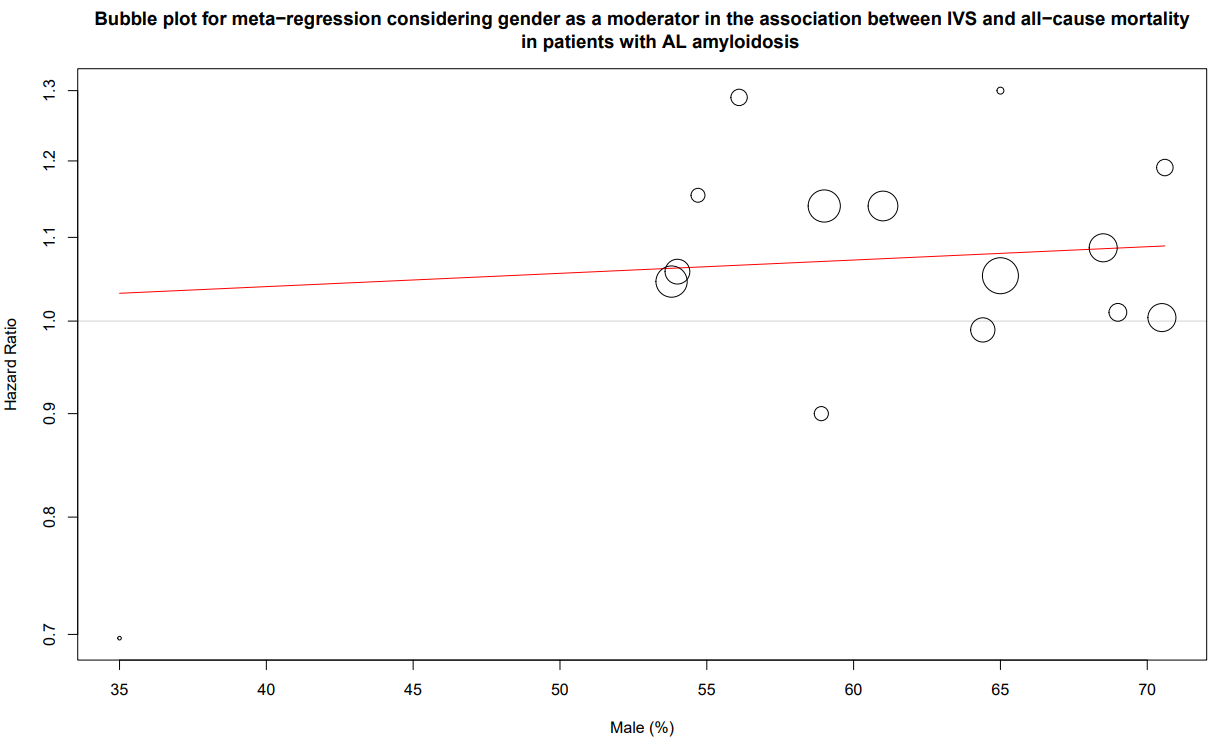


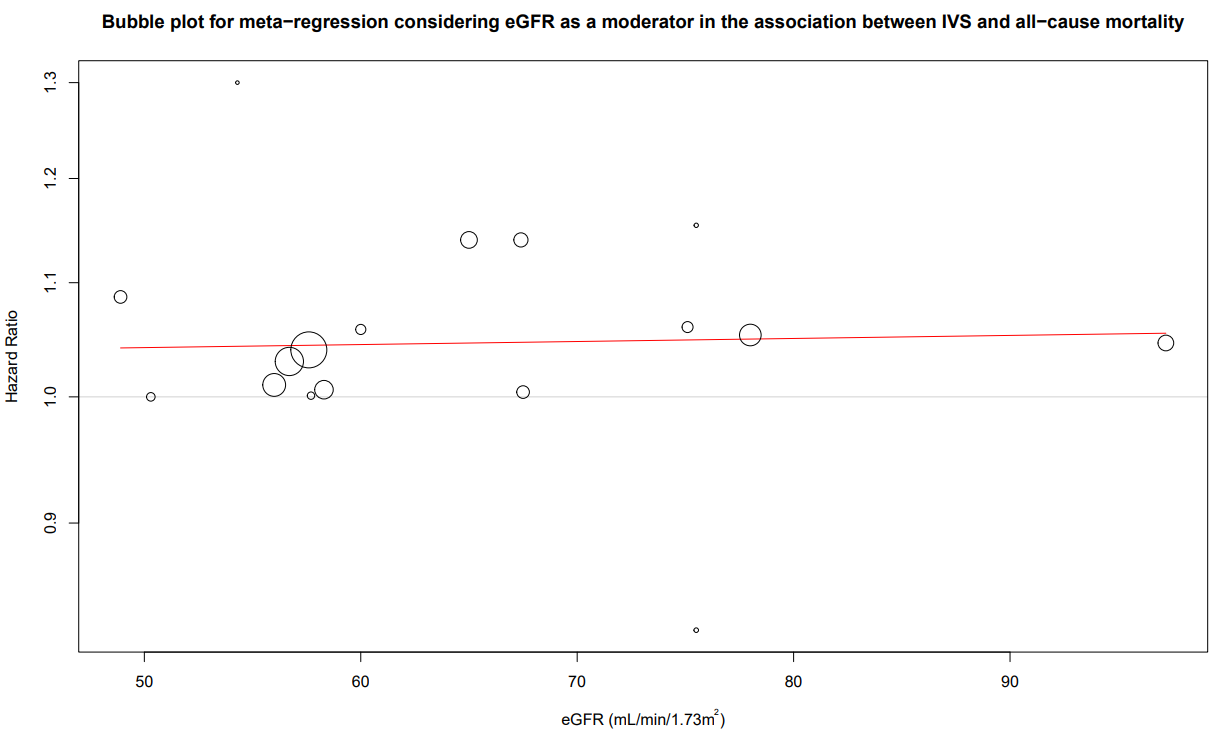


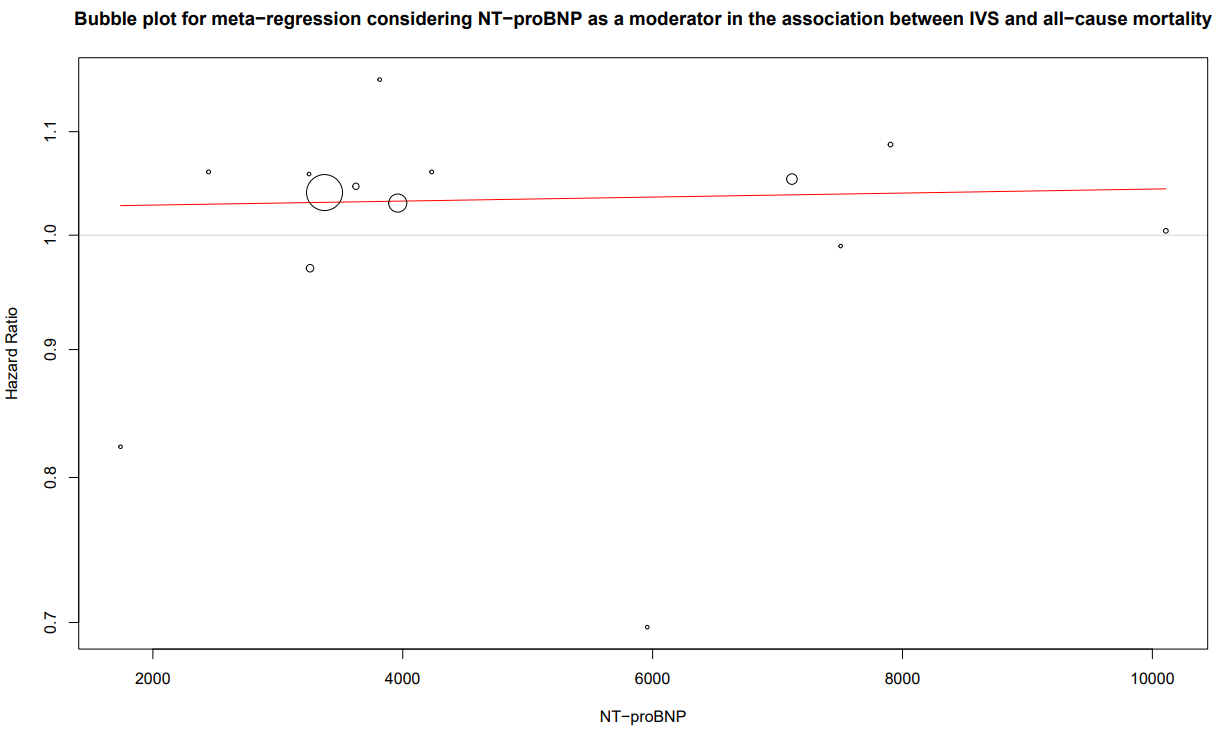


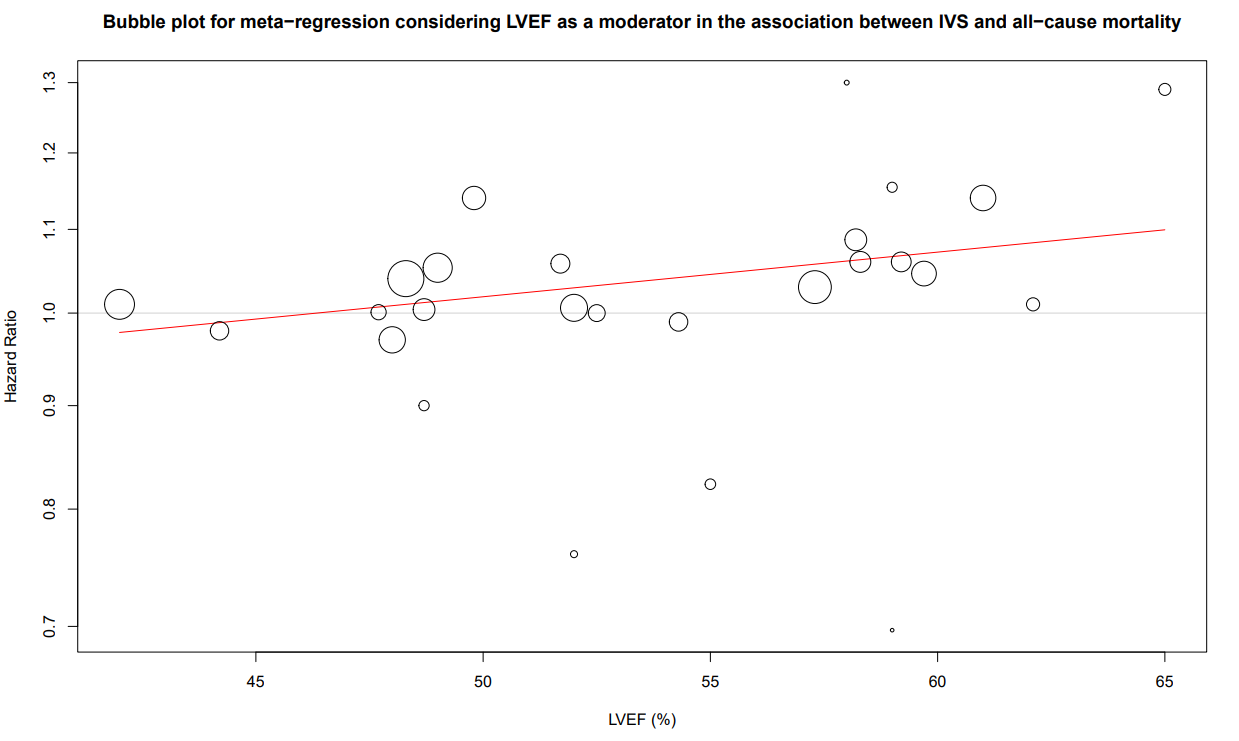


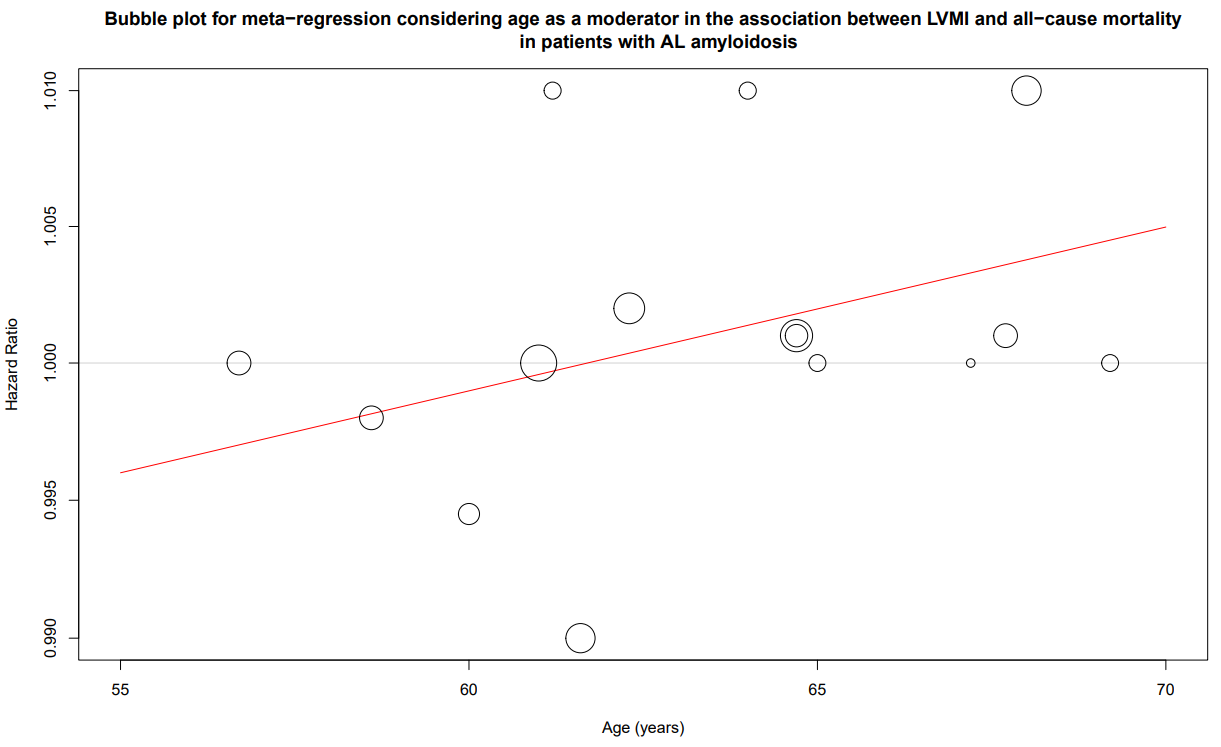


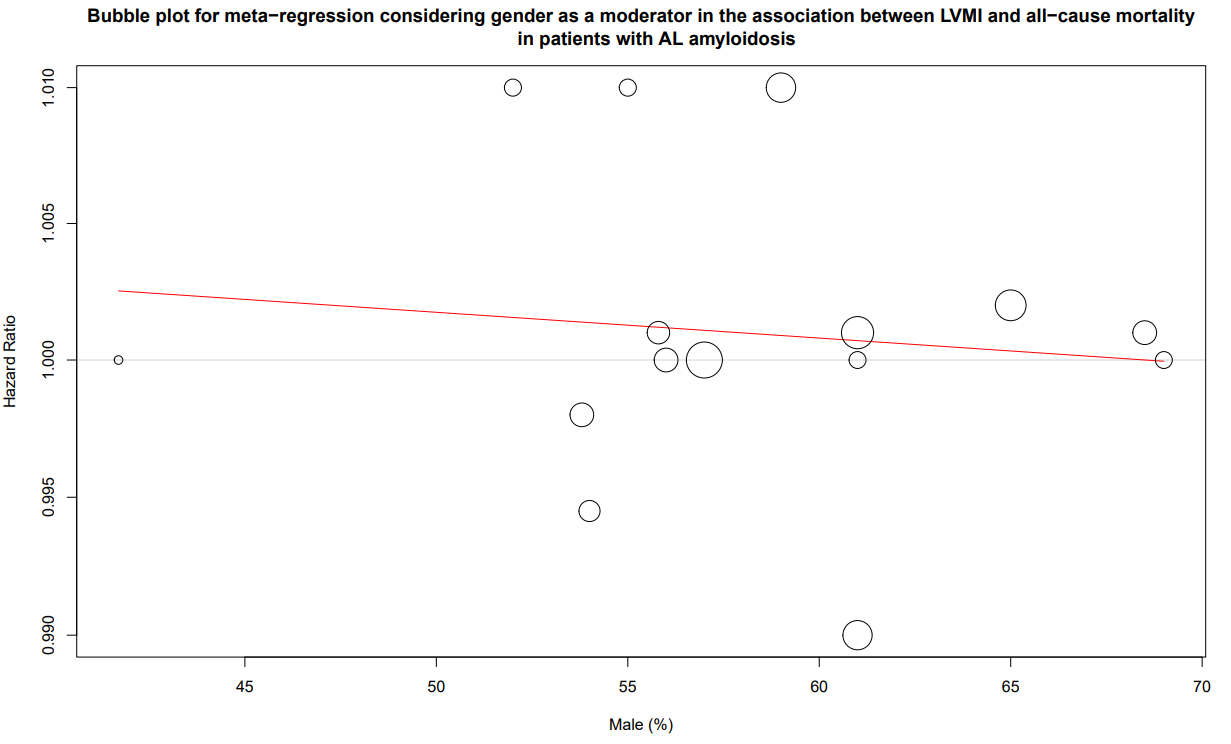


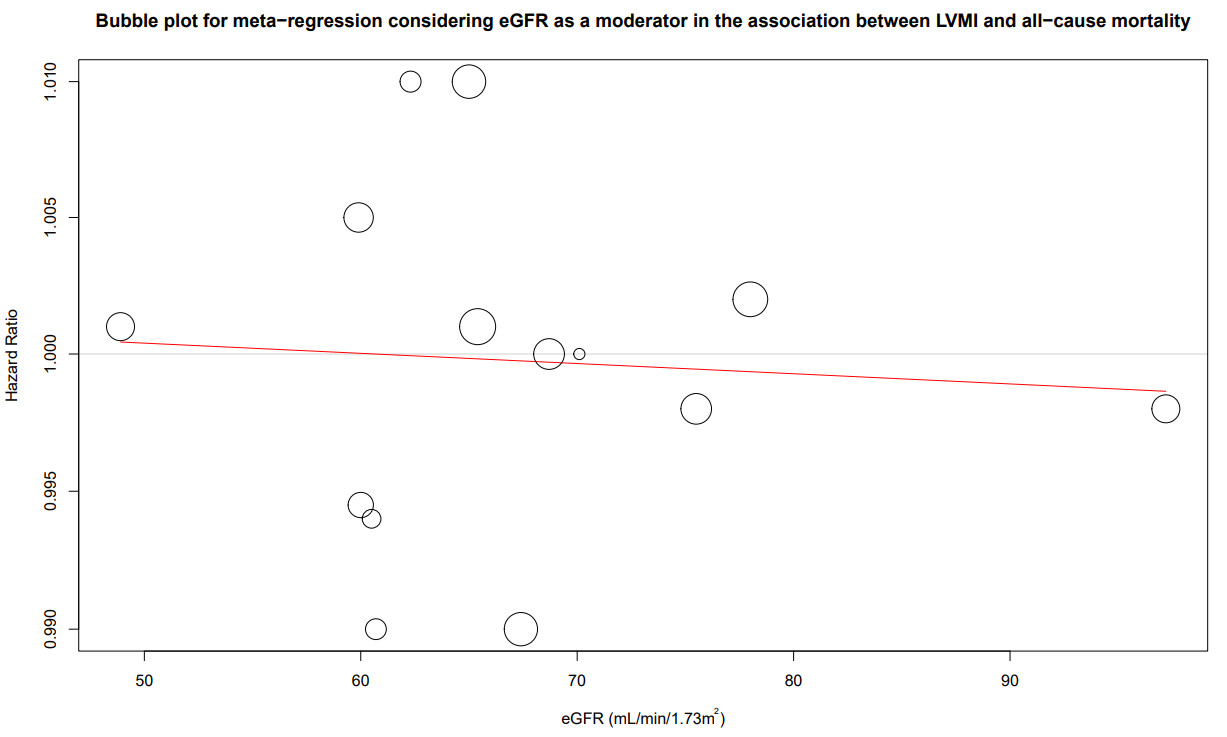


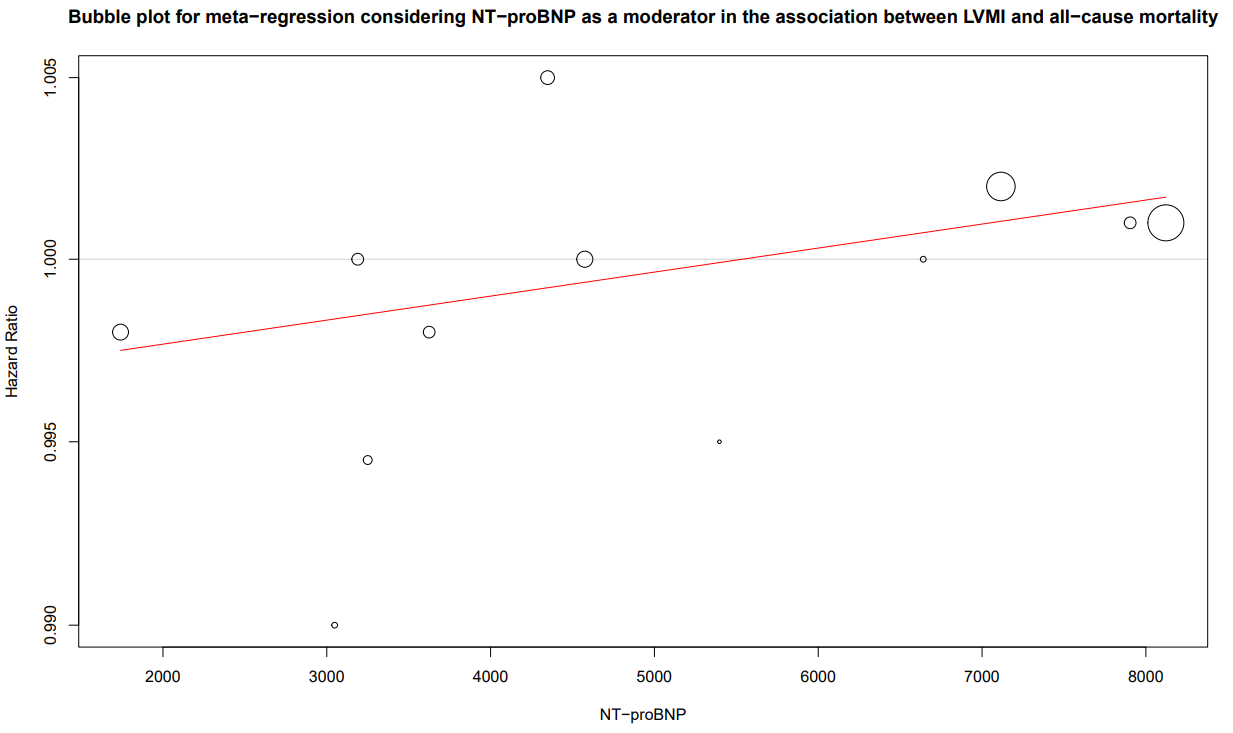


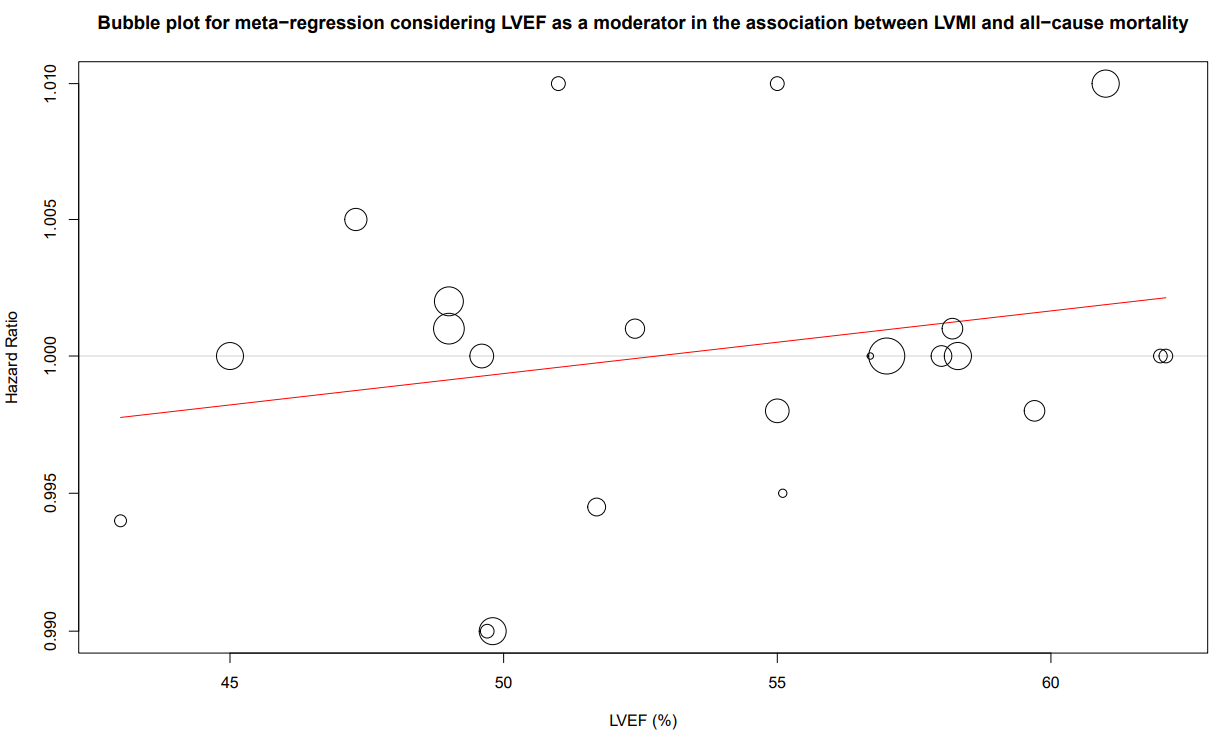


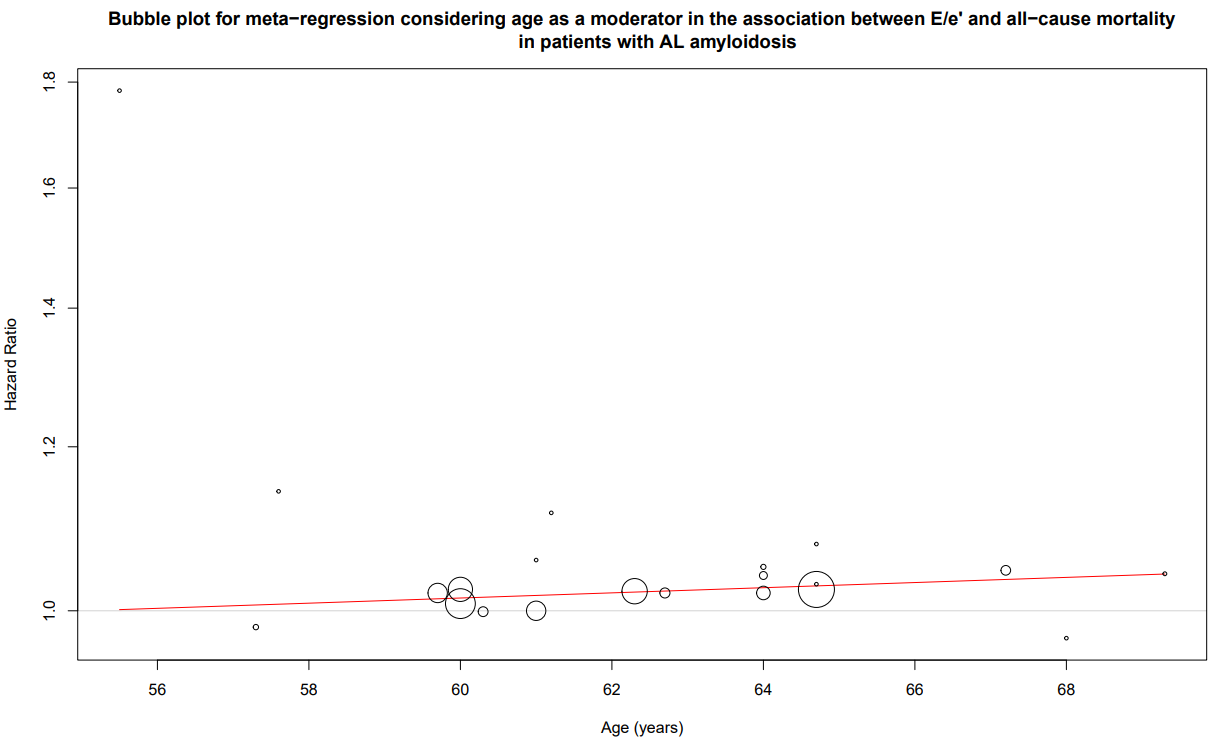


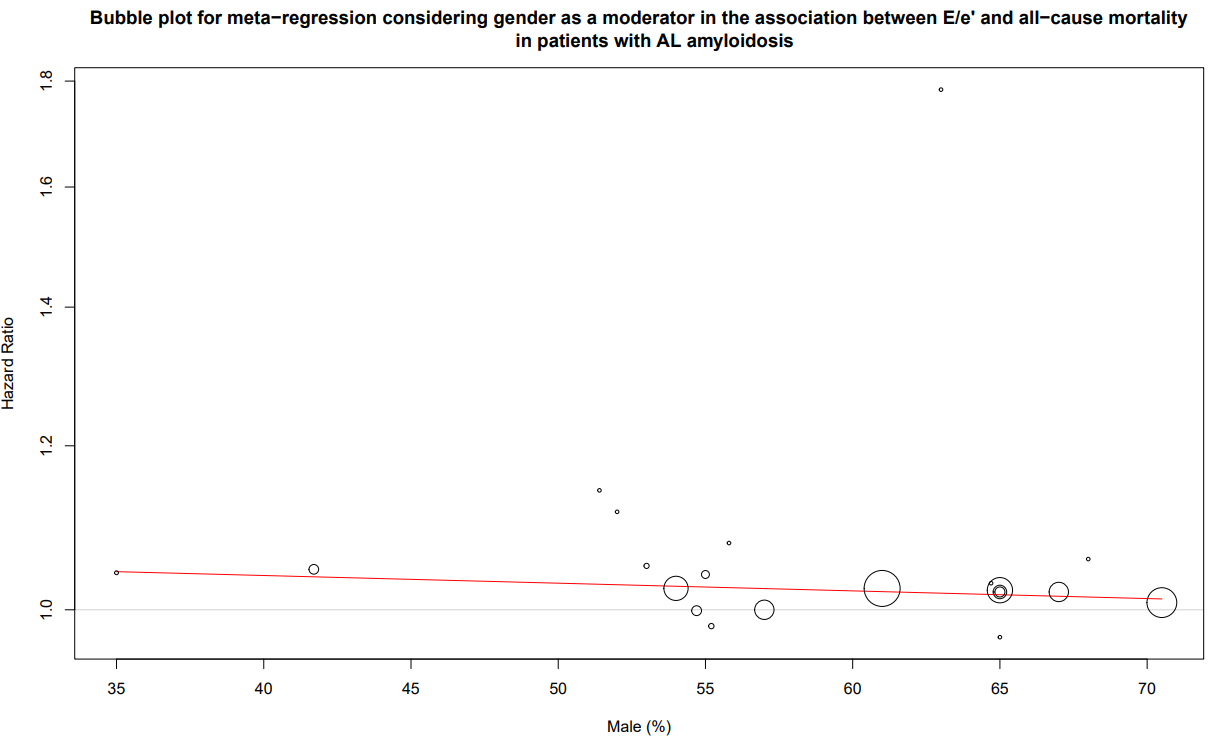


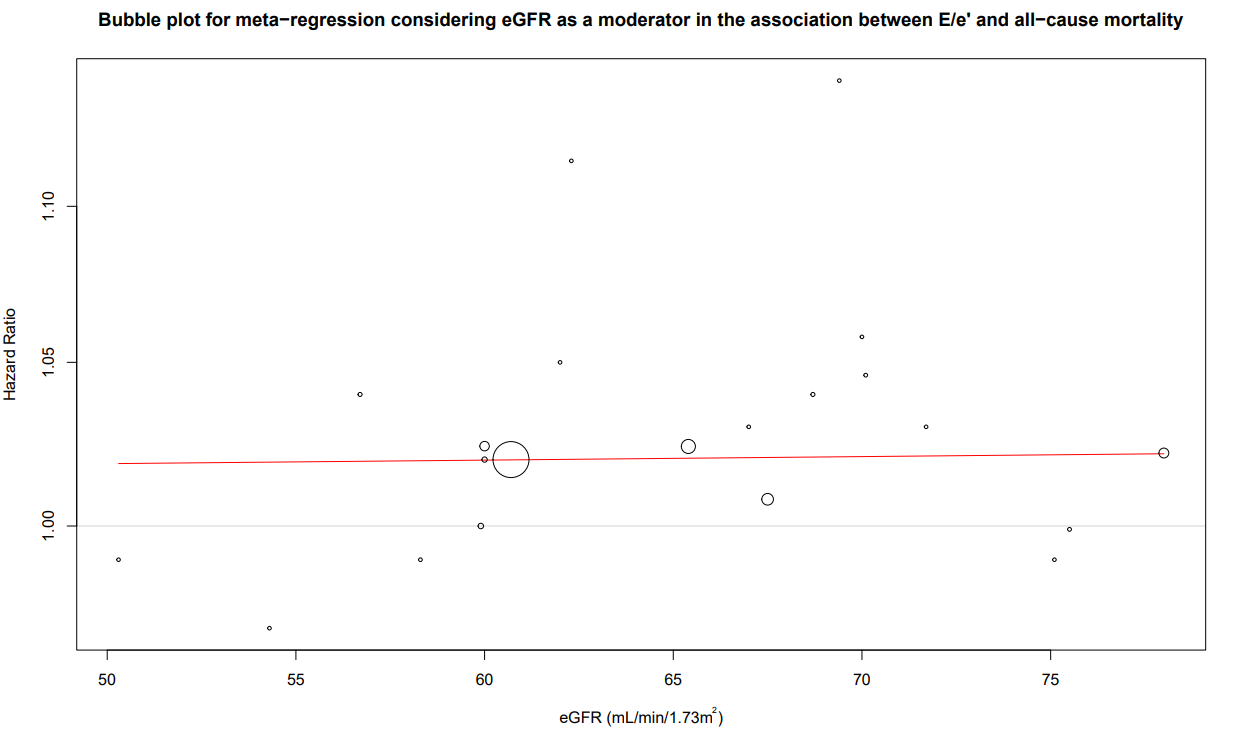


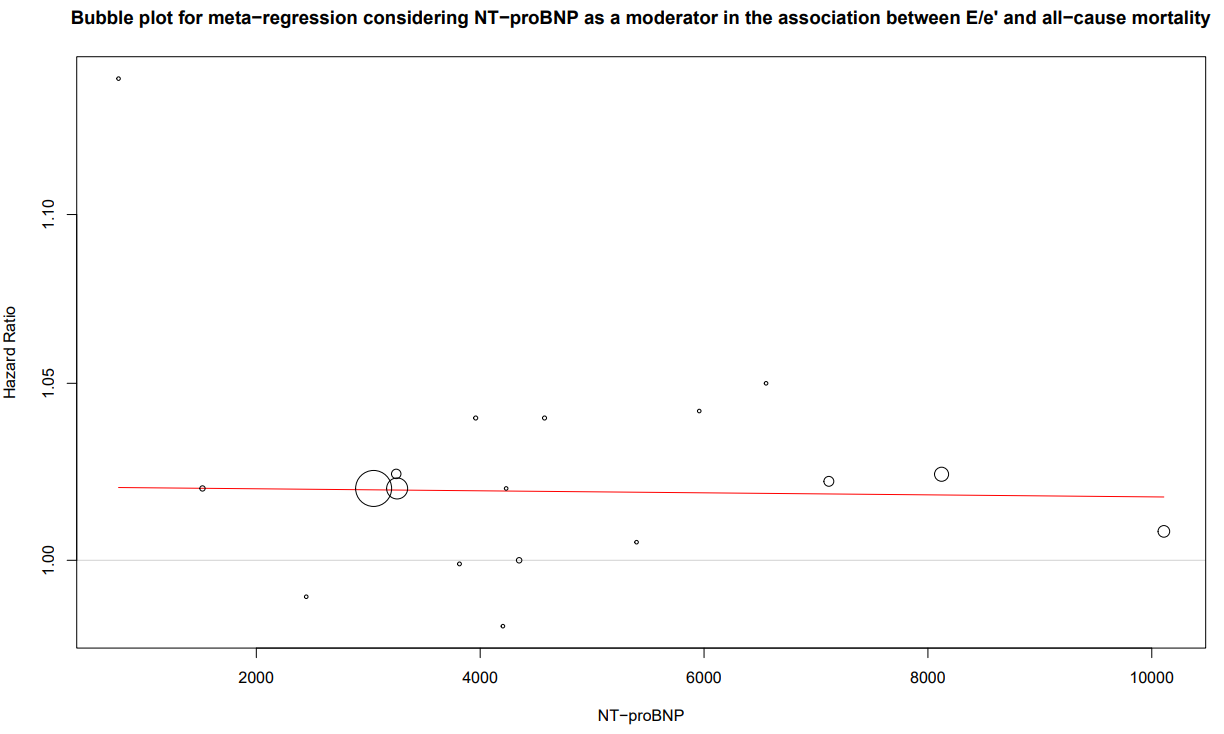


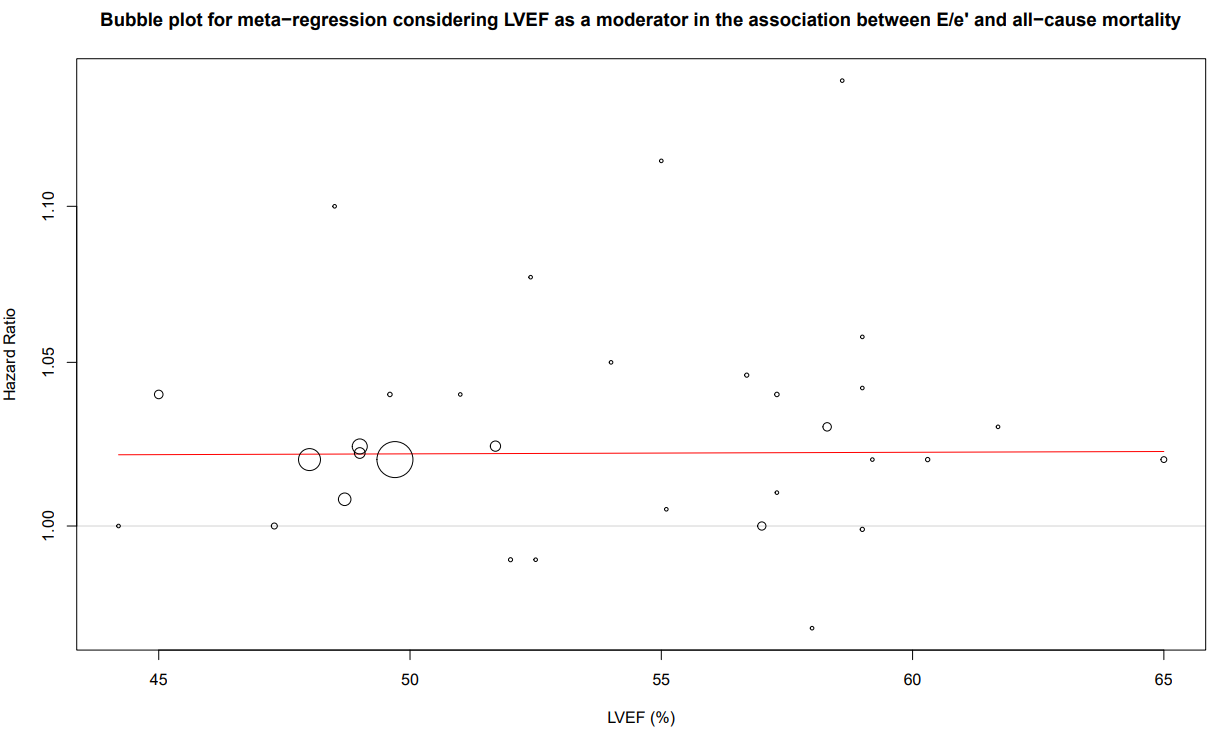


Appendix 3: Sensitivity analyses excluding studies that reported the composite endpoint of all-cause mortality and heart transplantation. Forest plots summarize the risk of all-cause mortality per A) 1% decrease in LV-GLS, B) per 1% decrease in RV-FWS, C) per 1% increase in LVEF, D) per 1mm increase in TAPSE, E) per 1mm increase in IVSd, F) per 1g/m2 increase in LVMi, and G) per 1 unit increase in the E/e’ ratio

A)


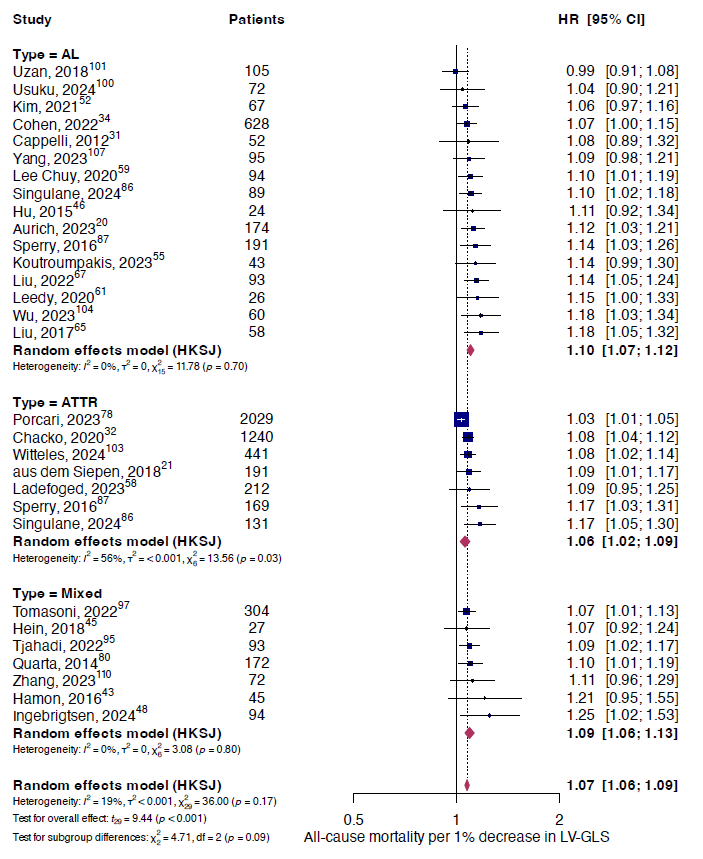


B)


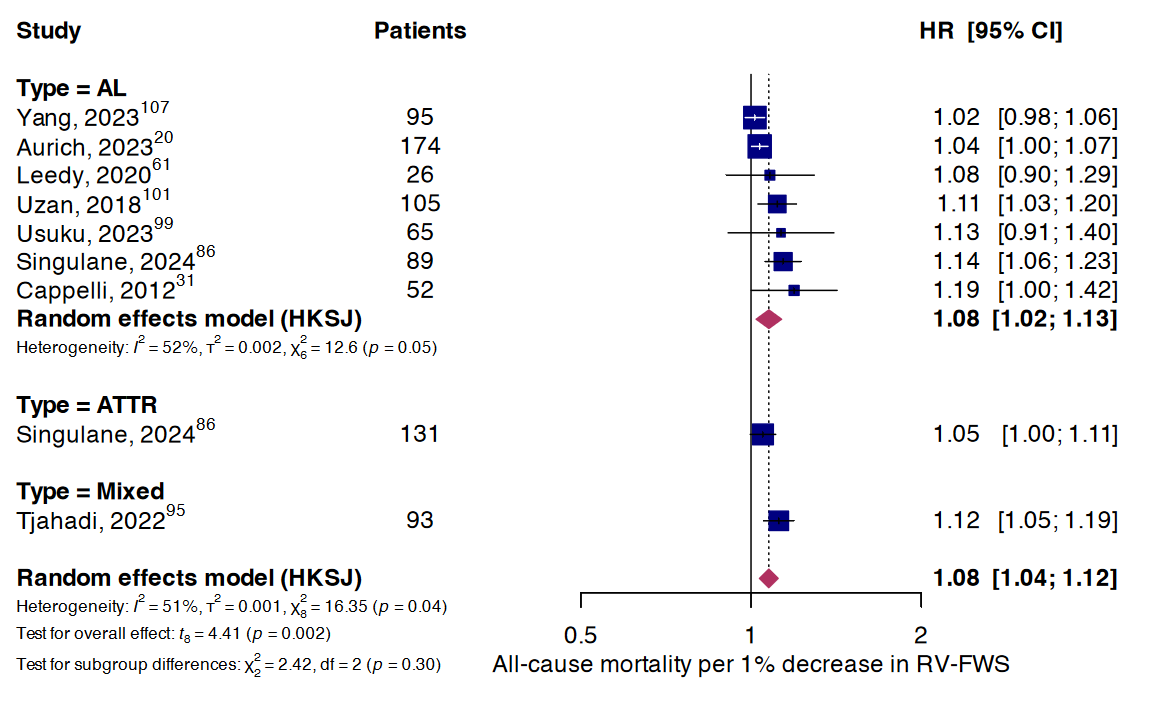


C)


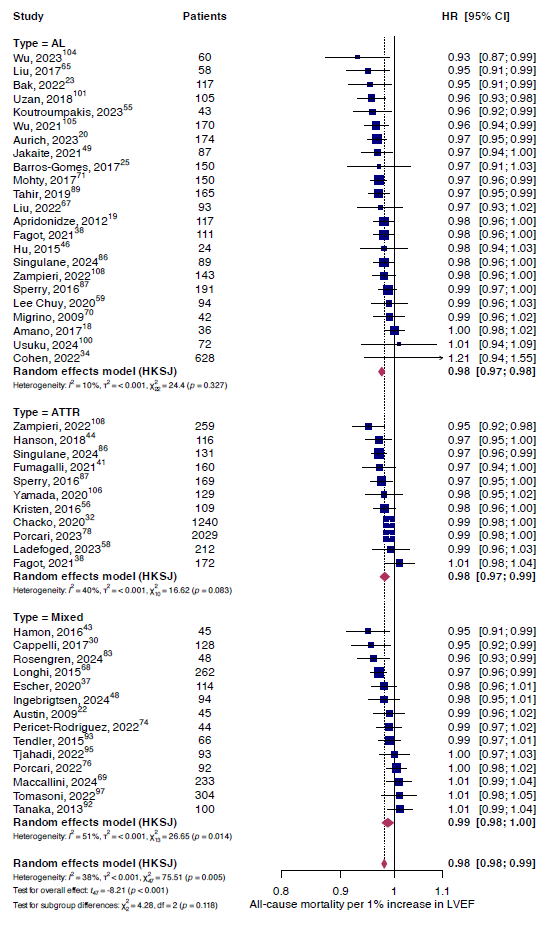


D)


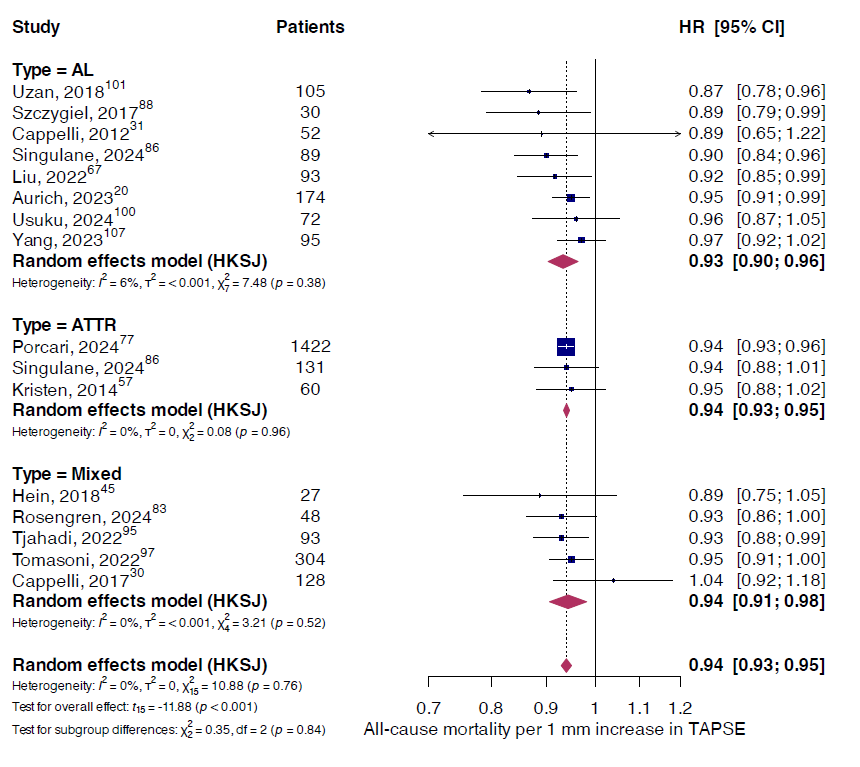


E)


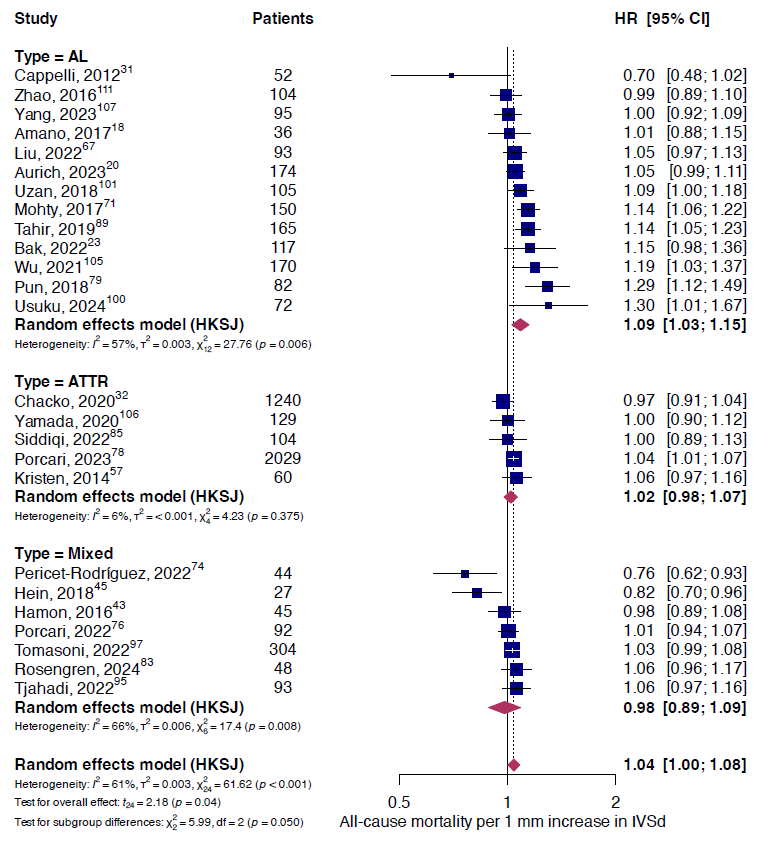


F)


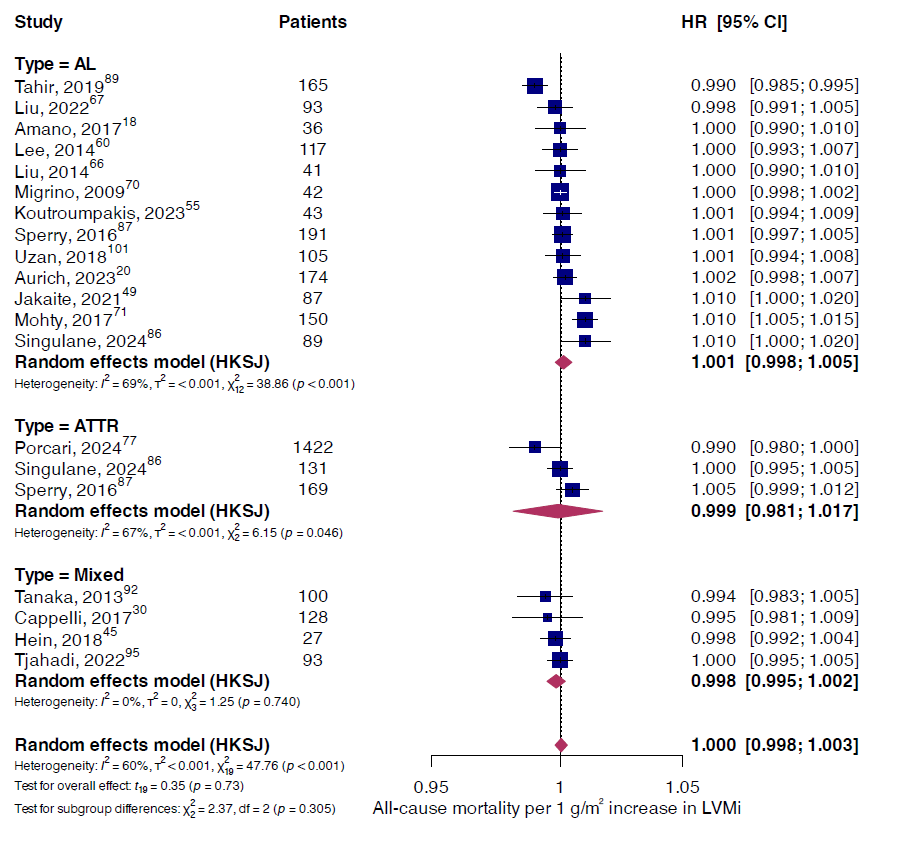


G)


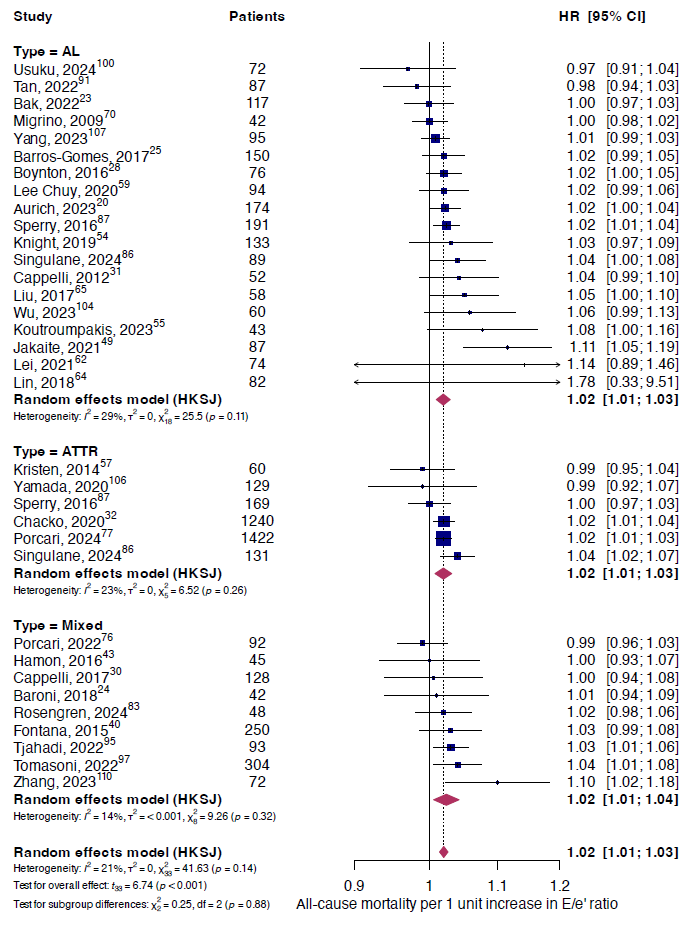


Appendix 4: Sensitivity analyses excluding the study by Porcari et al (2024), due to potential residual cohort overlap with Chacko et al (2020). Forest plots summarize the risk of all-cause mortality per A) 1% decrease in LV-GLS, B) per 1% increase in LVEF, C) per 1mm increase in IVSd, and D) per 1 unit increase in the E/e’ ratio

A)


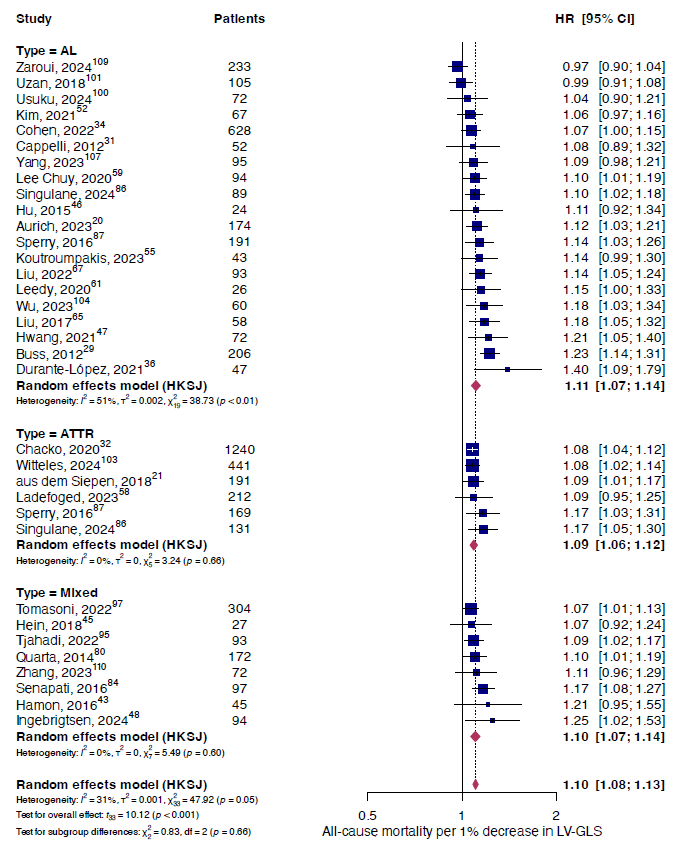


B)


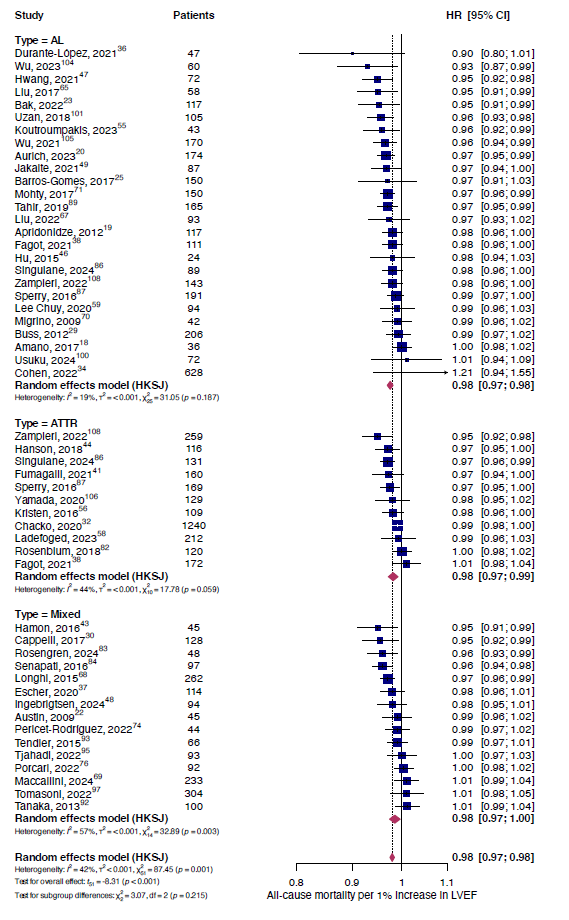


C)


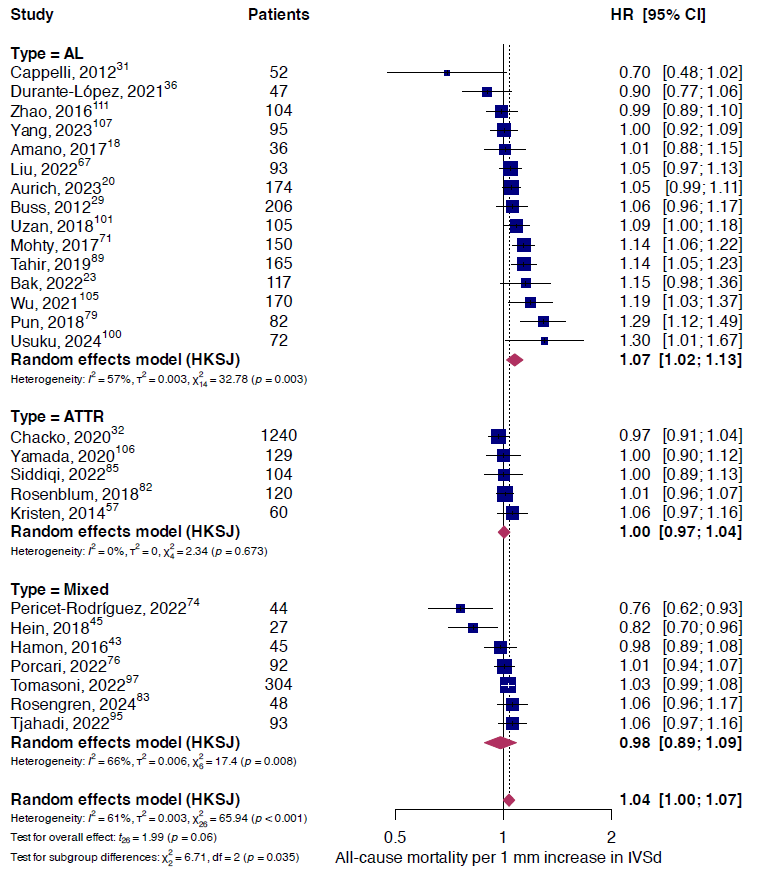


D)


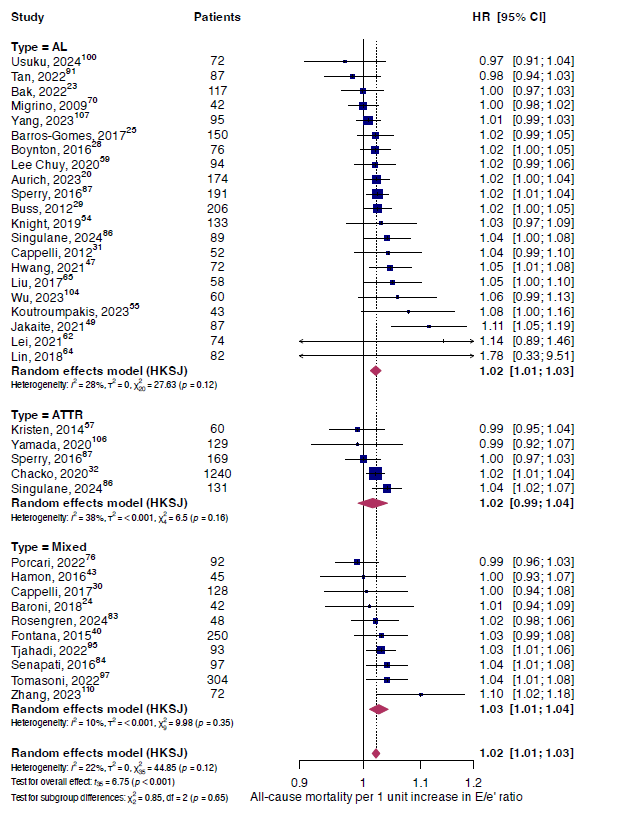


Appendix 5: Sensitivity analyses excluding historical cohorts (2009-2016). Forest plots summarize the risk of all-cause mortality per A) 1% decrease in LV-GLS, B) per 1% decrease in RV-FWS, C) per 1% increase in LVEF, D) per 1mm increase in TAPSE, E) per 1mm increase in IVSd, F) per 1g/m2 increase in LVMi, and G) per 1 unit increase in the E/e’ ratio

A)


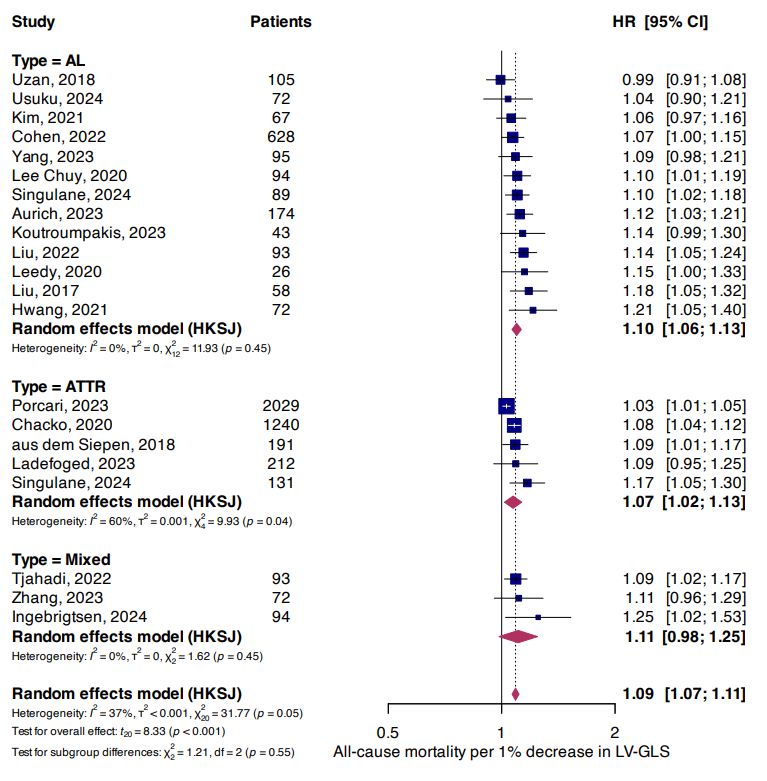


B)


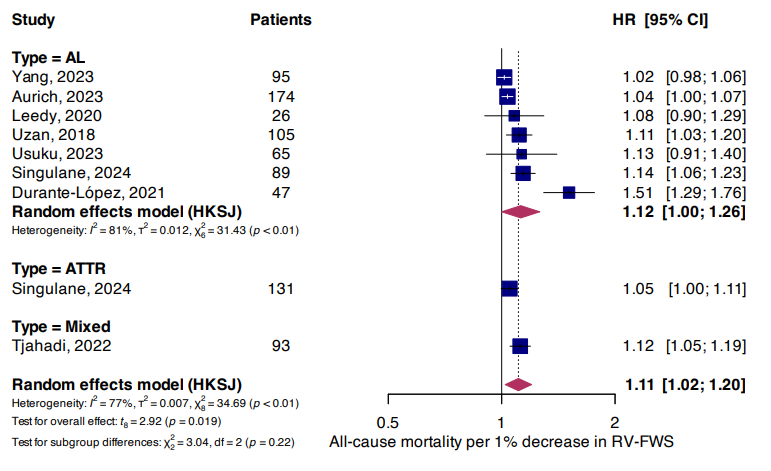


C)


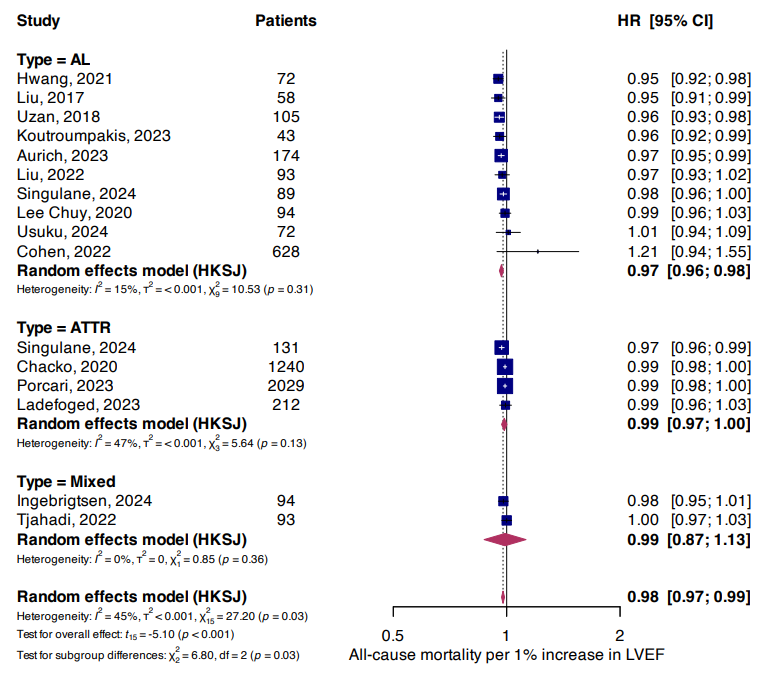


D)


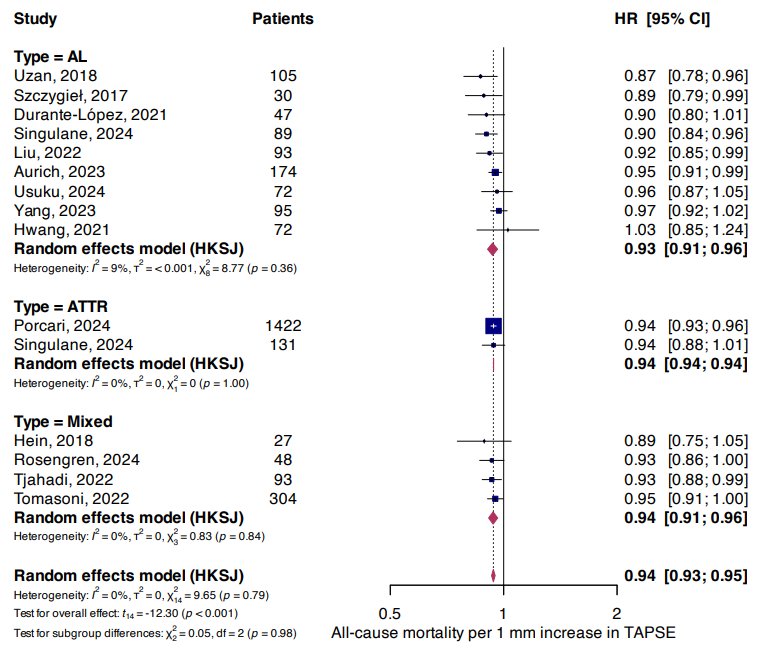


E)


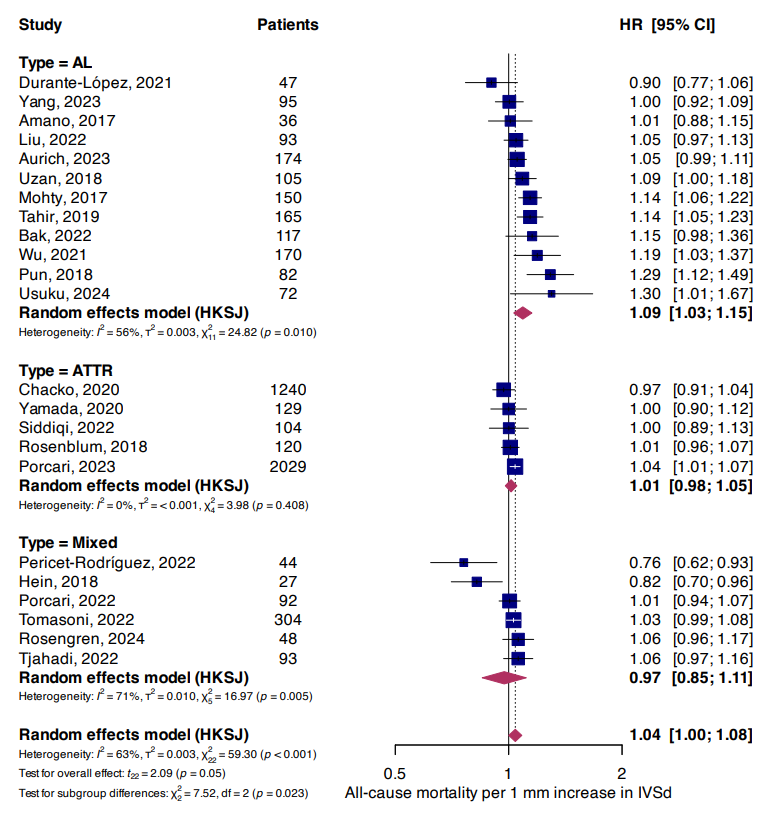


F)


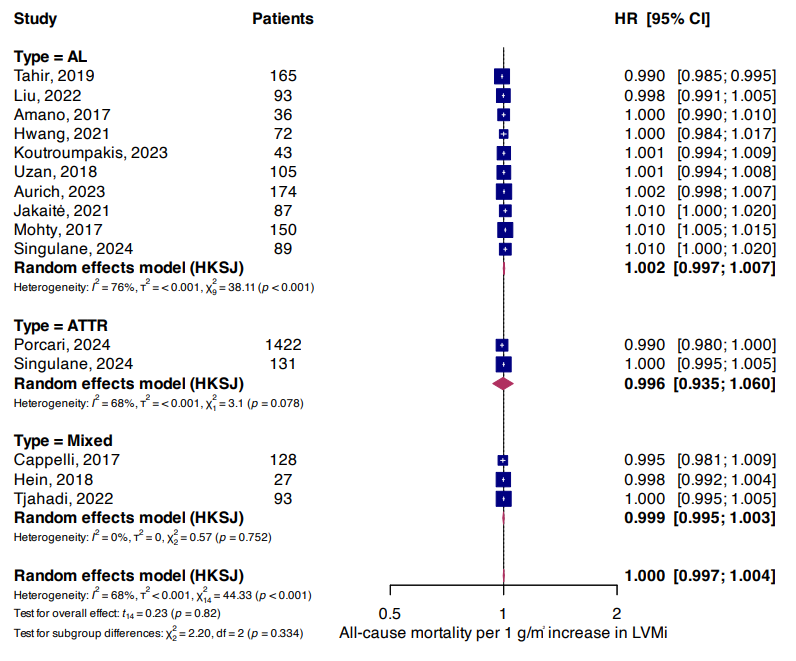


G)


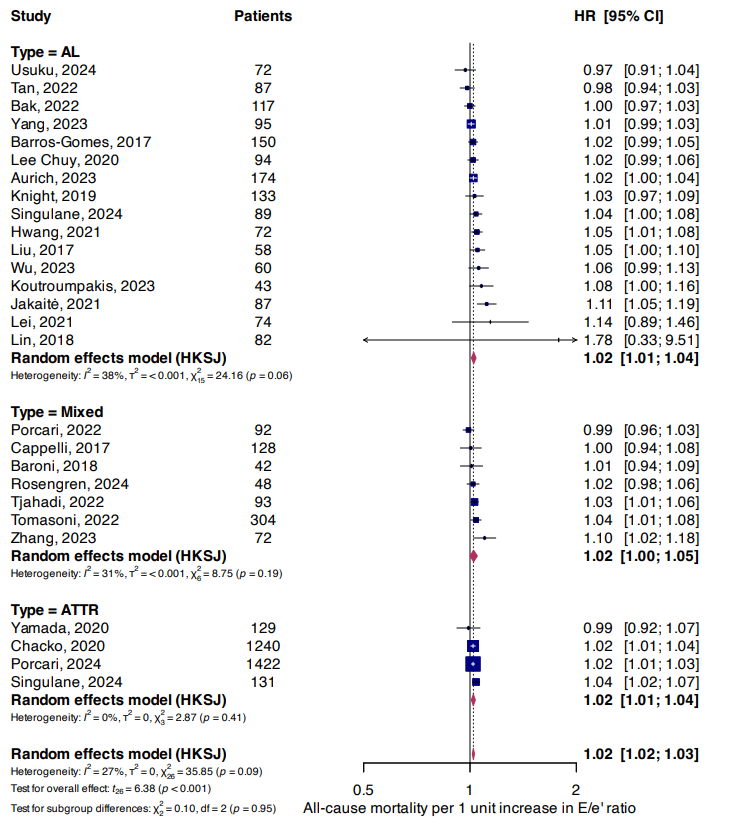


Appendix 6: Sensitivity analysis including studies with concurrent assessment of LV-GLS, LVMi and E/e’ ratios. Forest plots summarize the risk of all-cause mortality per A) 1% decrease in LV-GLS, B) per 1g/m2 increase in LVMi, and C) per 1 unit increase in the E/e’ ratio

A)**
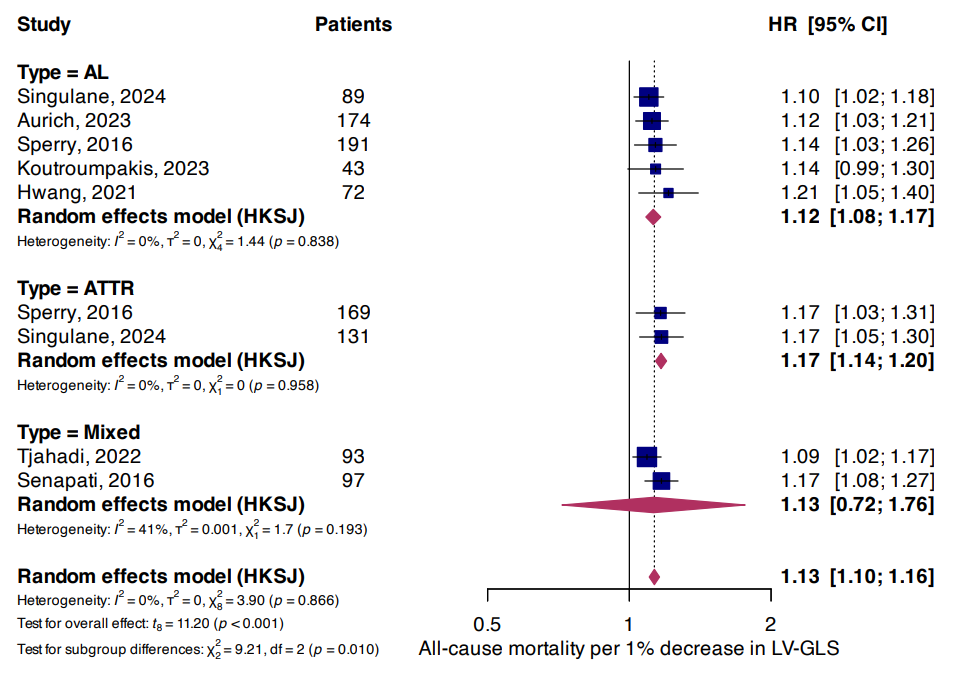
**

B)


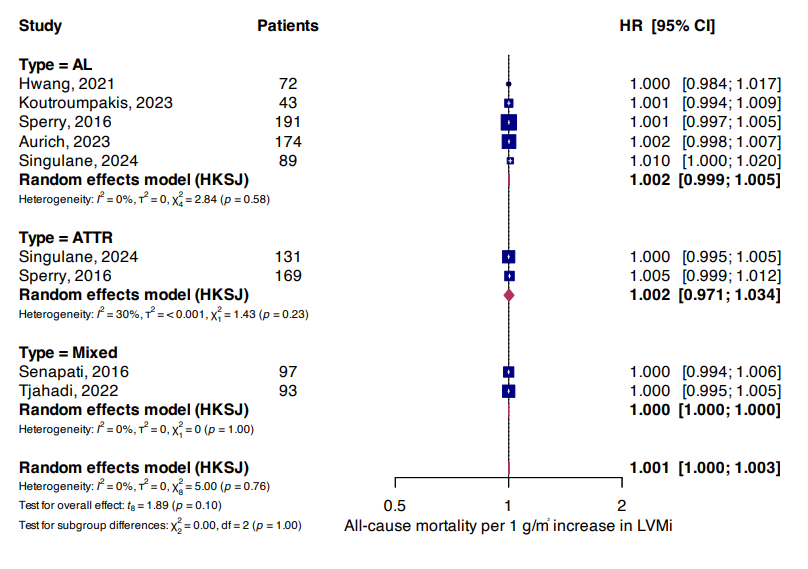


C)


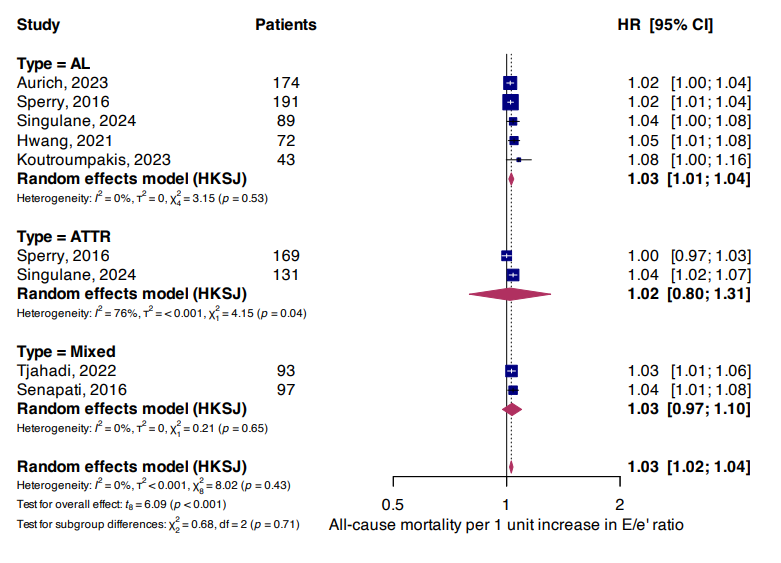


Appendix 7: Funnel plots for visual assessment of small-study effects


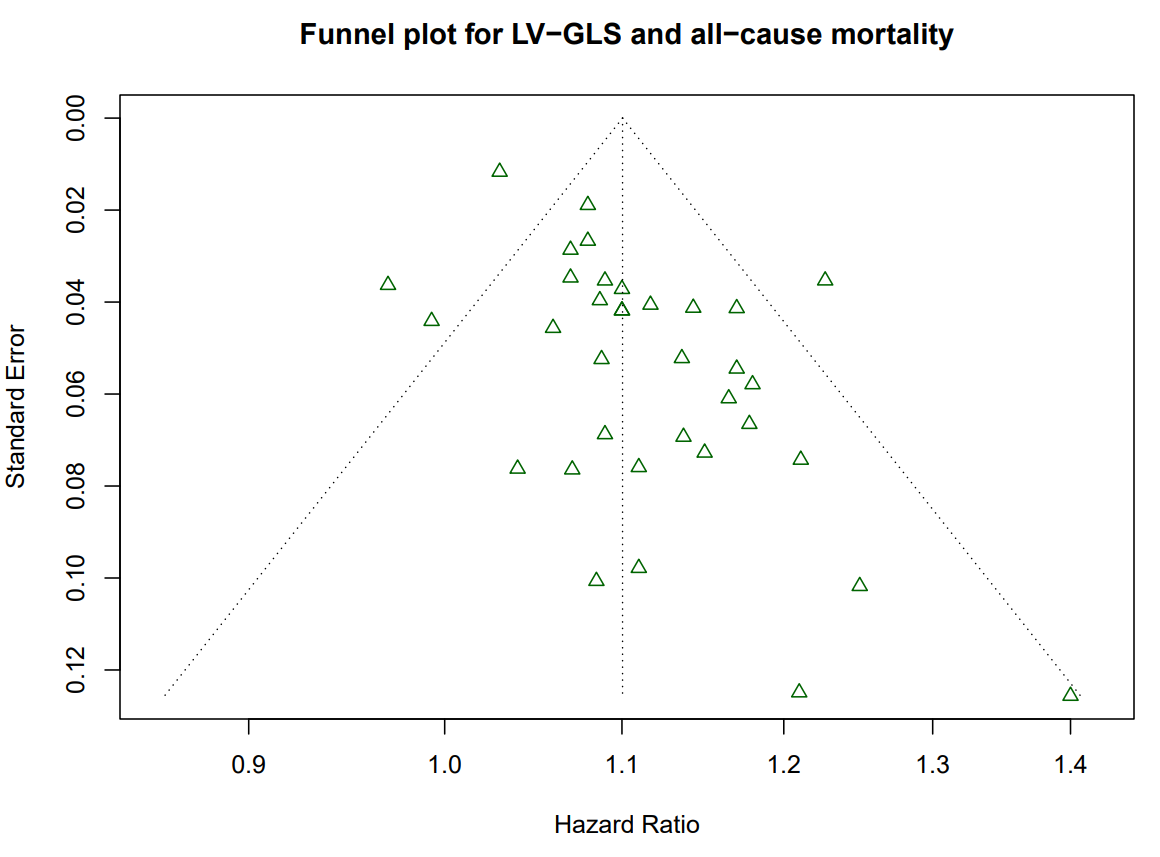


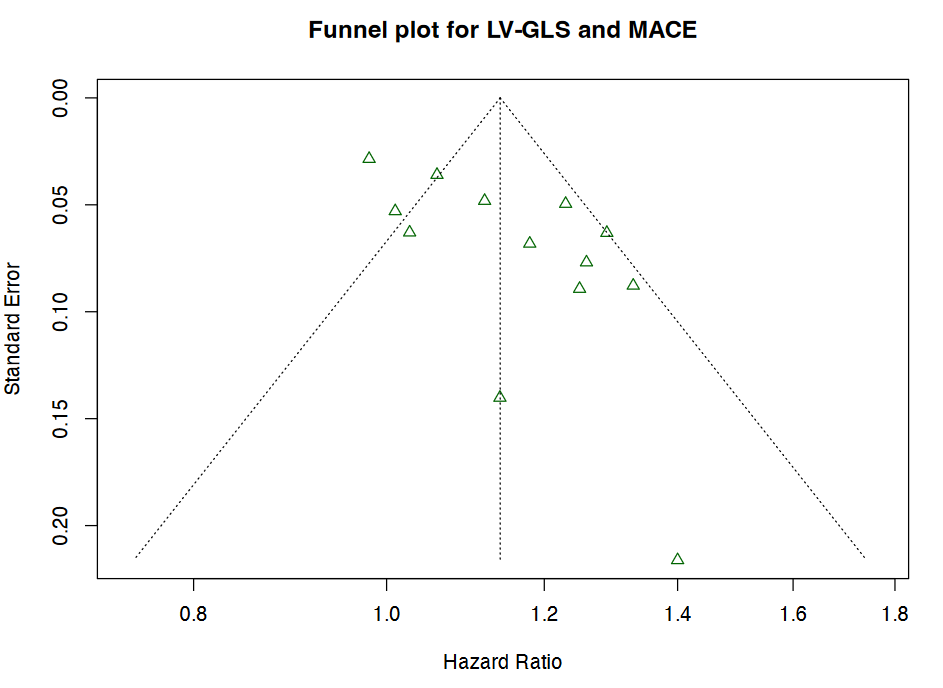


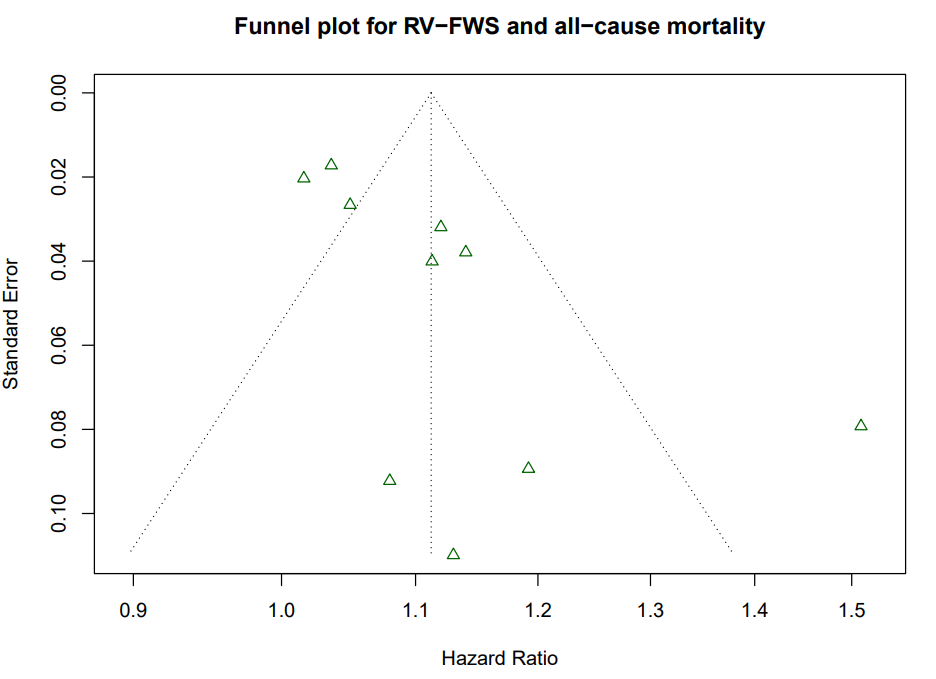


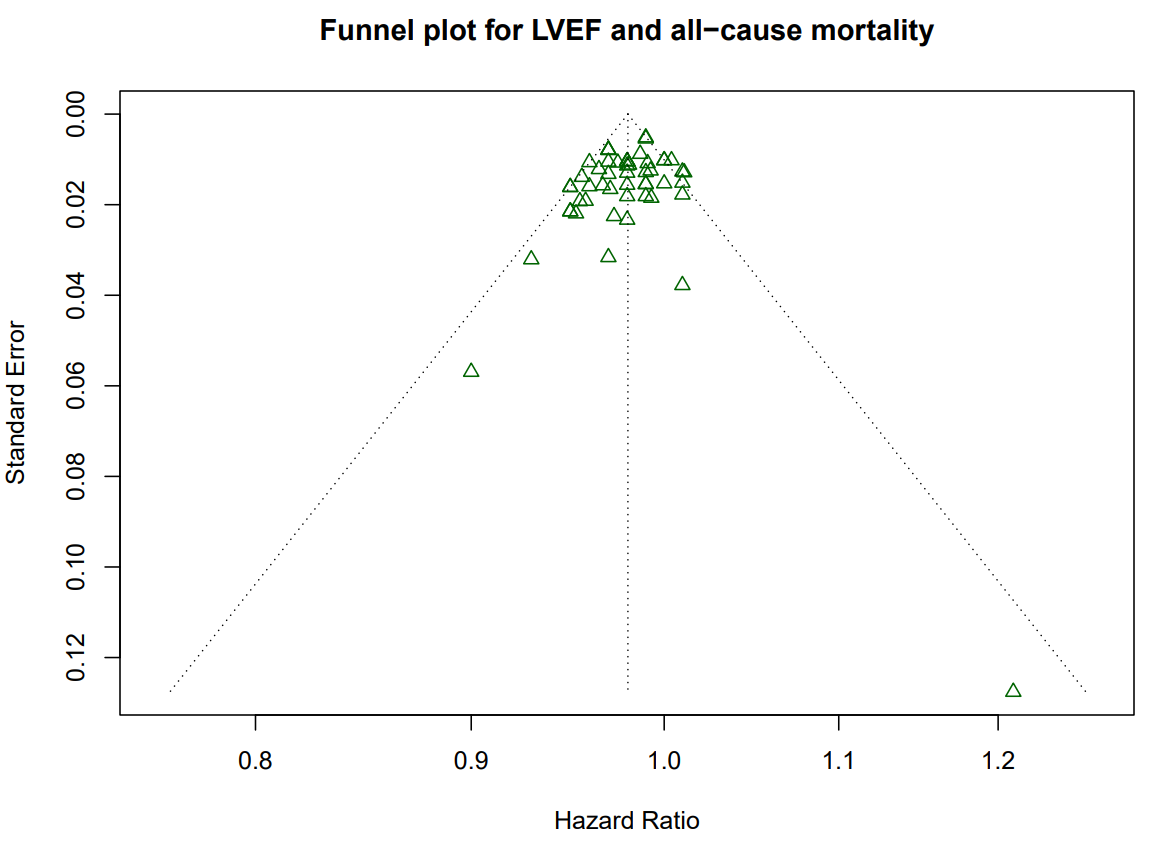


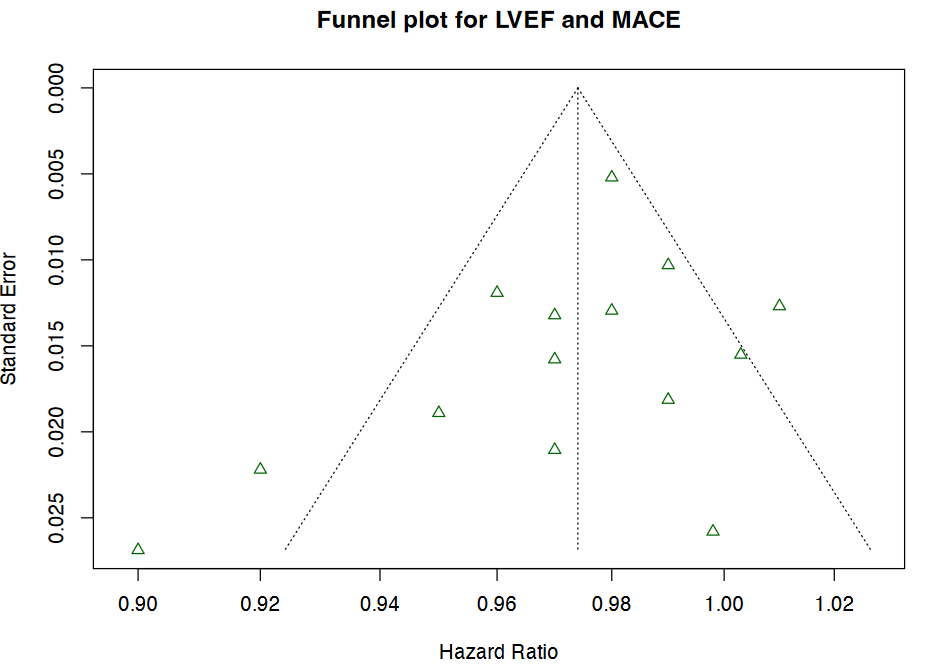


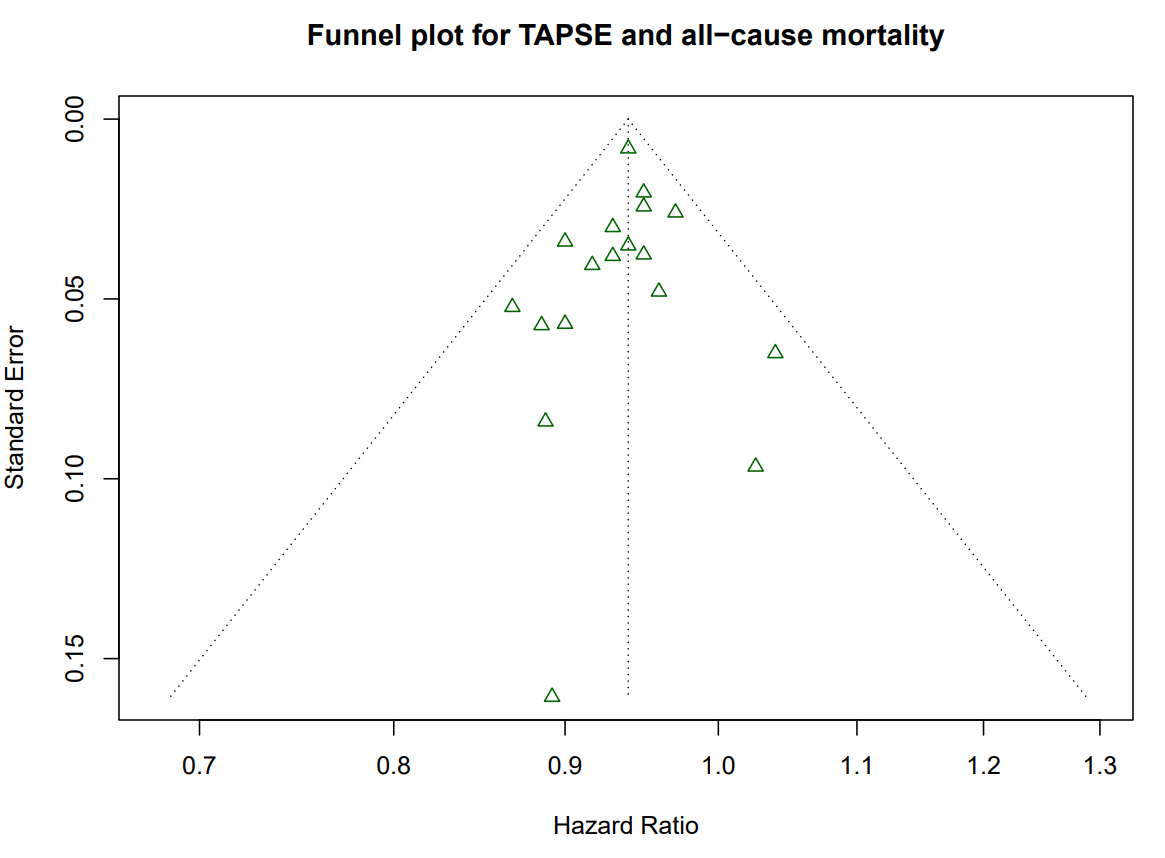


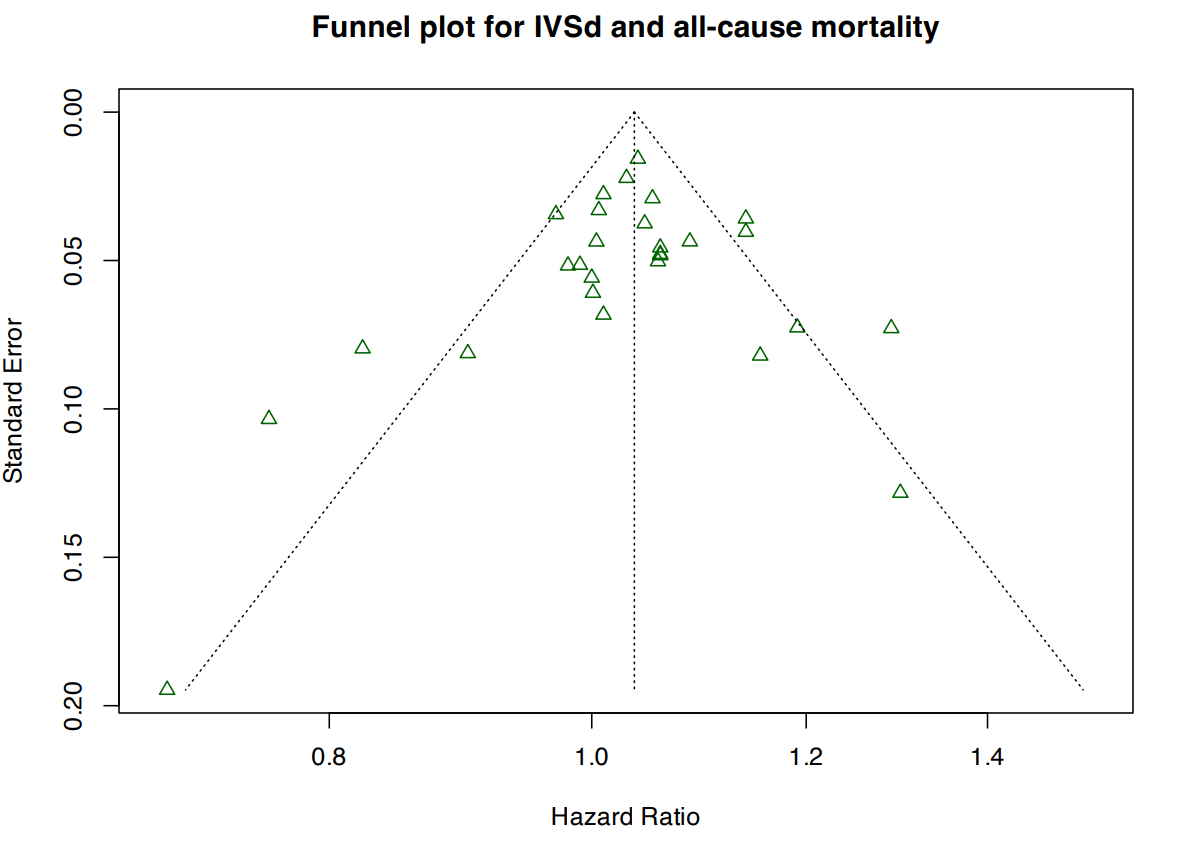


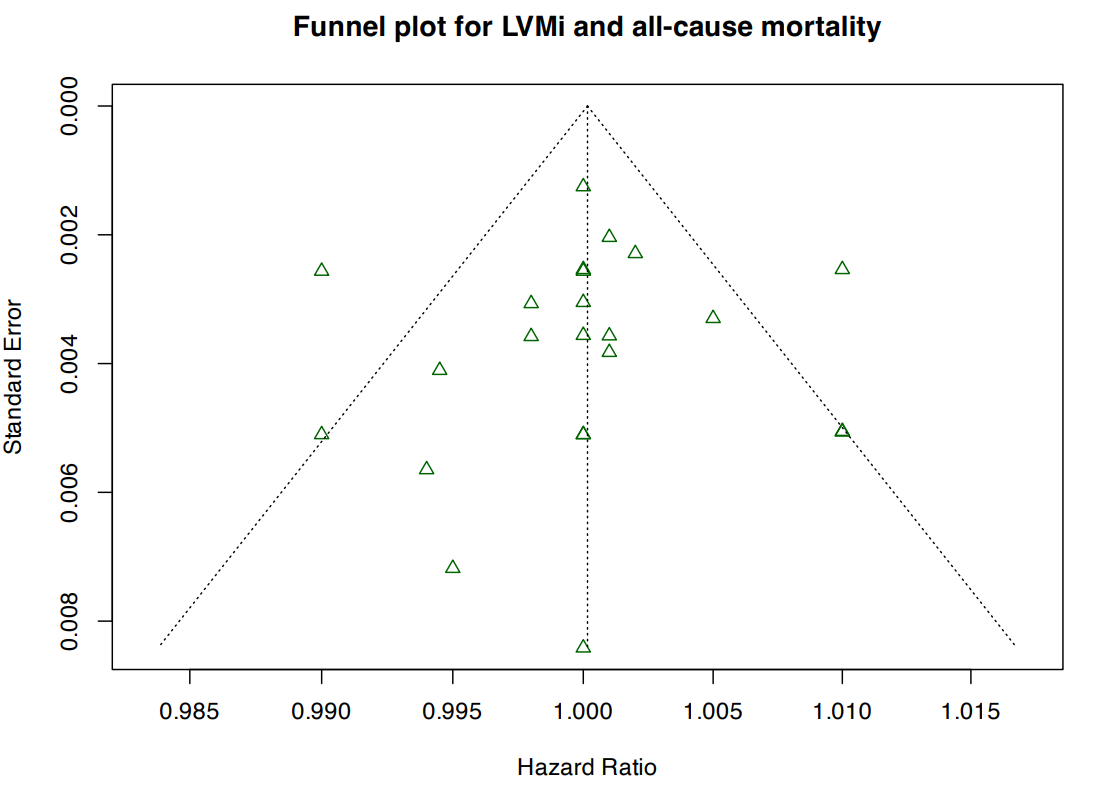


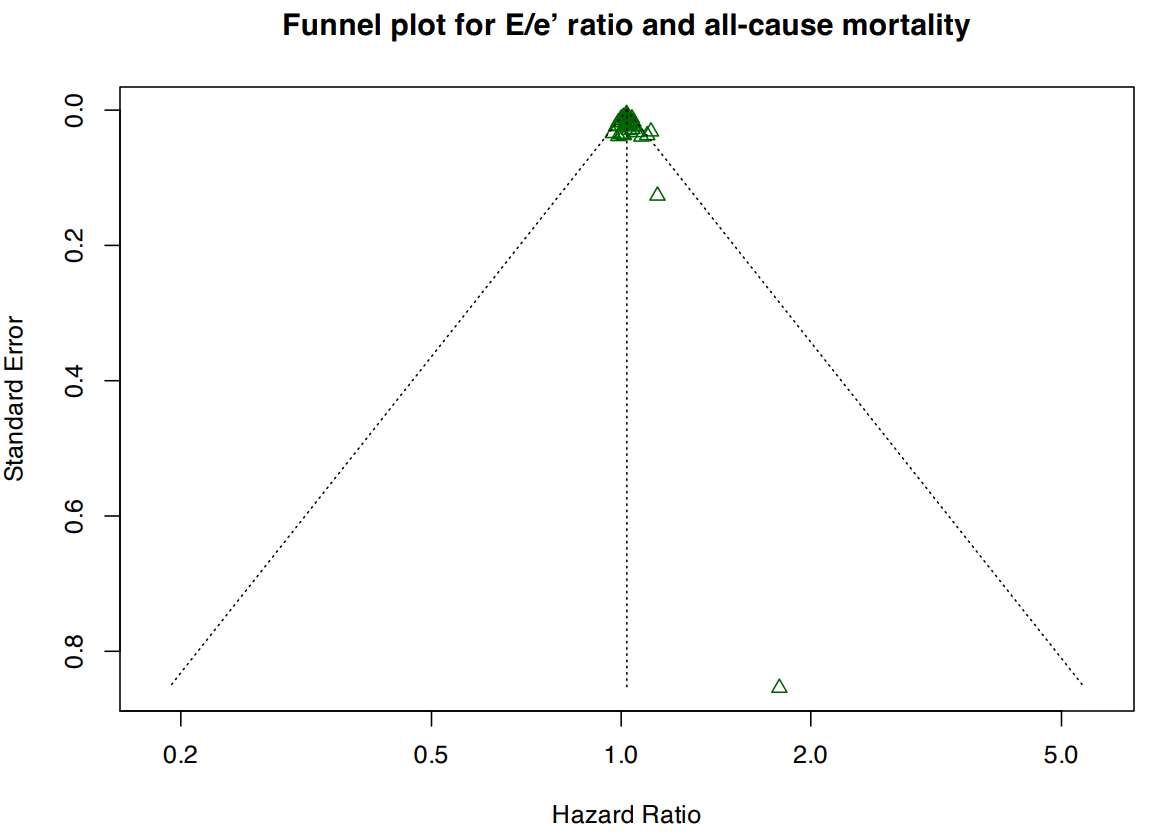

Supplement: oeaf078_Supplementary_Data [file oeaf078_supplementary_data.docx]
